# Dog–human vocal interactions match dogs’ sensory-motor tuning

**DOI:** 10.1371/journal.pbio.3002789

**Published:** 2024-10-01

**Authors:** Eloïse C. Déaux, Théophane Piette, Florence Gaunet, Thierry Legou, Luc Arnal, Anne-Lise Giraud

**Affiliations:** 1 Department of Basic Neurosciences, Faculty of Medicine, University of Geneva, Geneva, Switzerland; 2 Aix-Marseille University and CNRS, Laboratoire de Psychologie Cognitive (UMR 7290), Marseille, France; 3 Aix Marseille University and CNRS, Laboratoire Parole et Langage (UMR 6057), Aix-en-Provence, France; 4 Université Paris Cité, Institut Pasteur, AP-HP, Inserm, Fondation Pour l’Audition, Institut de l’Audition, IHU reConnect, F-75012 Paris, France; Eotvos Lorand University, HUNGARY

## Abstract

Within species, vocal and auditory systems presumably coevolved to converge on a critical temporal acoustic structure that can be best produced and perceived. While dogs cannot produce articulated sounds, they respond to speech, raising the question as to whether this heterospecific receptive ability could be shaped by exposure to speech or remains bounded by their own sensorimotor capacity. Using acoustic analyses of dog vocalisations, we show that their main production rhythm is slower than the dominant (syllabic) speech rate, and that human–dog-directed speech falls halfway in between. Comparative exploration of neural (electroencephalography) and behavioural responses to speech reveals that comprehension in dogs relies on a slower speech rhythm tracking (delta) than humans’ (theta), even though dogs are equally sensitive to speech content and prosody. Thus, the dog audio-motor tuning differs from humans’, and we hypothesise that humans may adjust their speech rate to this shared temporal channel as means to improve communication efficacy.

## Abstract 2

Human-to-pet communication requires speech processing by the animal and adjustments of the human speaking rate to match their pet’s receptive abilities. This study shows that dogs and humans share similar but not identical speech processing mechanisms and that dog-human vocal interactions match dogs’ sensory-motor tuning.

## Introduction

Acoustic communication dynamically evolves as auditory systems are tuned to vocal signals, while in turn vocal production adapts to exploit the capacity of sensory systems [1–4]. In this fine audio-vocal tuning, temporal acoustic features have a universal ecological relevance, being essential, for example, vocal recognition [5,6], predator avoidance [7], or mate choice [8–10].

Production and perception systems can also evolve as a result of interspecific interactions, e.g., in the prey–predator arm race [11] or during interspecific eavesdropping [12]. One of the most prominent and long-term examples of interspecific interactions is that of dogs, *Canis familiaris*, and humans. It is likely that the dog–human cooperation needed to achieve working tasks, i.e., hunting, hauling, and sentinel work required the development of effective interspecific communication skills [13,14]. In terms of vocal production, quantitative and qualitative hypertrophy of bark vocalisations, for example, is believed to be a reflection of the necessity to develop a human-targeted repertoire [15,16]. Concurrently, dog owners speaking to their pet naturally employ accented speech modulations, known as dog-directed speech [17–19]. Notably, dog-directed speech differs from other forms of animal-directed speech, suggesting that humans adjust their speech based on their target audience, instead of having a universal animal-directed speech register [20]. Such an adaptation is also the basis of infant-directed speech that optimises communication with infants’ immature auditory system whose speech processing relies predominantly on the reception of prosodic rhythms that progressively shape faster rhythms [21,22].

Perceptually, dogs exhibit remarkable receptivity to human speech cues [23–26], can learn extensive word repertoires [27], demonstrate fast mapping [28] and statistical learning abilities [29], and possibly word-elicited mental representations [30]. Moreover, behavioural evidence suggests that humans possess a unique sensitivity to dogs’ acoustic signals, as sightless individuals without prior experience with dogs can accurately assess emotional valence from dog vocalisations [31], and humans generally demonstrate greater proficiency in evaluating the emotional valence of dog vocalisations compared to those of other species [32,33]. Collectively, these findings suggest that humans’ accurate perception of dogs’ acoustic signals, even without ontogenic experience, cannot be solely explained by cross-species rules of emotional information transfer, but may reflect interspecific communicative adaptation.

However, one question that remains open is the extent to which dog–human vocal interactions may have been shaped by either species’ production/perception constraints in the temporal domain. Indeed, exploration of the speech system has provided capital insight into the neural bases of the temporal perception/production tuning required for successful intraspecific vocal communication. Speech rhythms are mechanically constrained by the motor effectors, but also operated within a certain dynamic range to best match perception-action neural rhythms. Thus, the dominant speech rhythm, the syllable rate, is cross-culturally stable [34] because it both arises from the interplay of the different articulators [35,36] and corresponds to neural theta oscillations, involved in active sensing across species [37]. In speech perception, the auditory theta rhythm serves to actively interface the acoustics with endogenous neural processes, and the closer the acoustics to this rhythm the more efficient the information transfer. Crucially, the neural theta rhythm can flexibly adapt to speech quasiperiodicity via a mechanism referred to as “speech tracking” [38], and comprehension critically depends on its precision [39–44]. Thus speech production and reception tuning has led to a common temporal window of analysis centred on the 4 to 8 Hz range [45].

However, dogs lack the vocal/neural system necessary to produce articulated communication [46–48], such that they may not have developed the neural machinery needed to perceive theta-based speech signals. While there may have been no specific dog–human adaptation, it is also possible to hypothesise that either the dog’s neural system has adapted to human speech or conversely that humans have adjusted their vocal production to exploit the dogs’ neural (auditory) capacity.

To address these questions, we first analysed dog vocalisations, as well as adult- (ADS) and dog-directed speech (DDS), to probe whether dogs vocalise at the same or at a different rate than humans, and whether the temporal properties of DDS differ from those of ADS. Second, we compared speech neural processing in dogs and humans using noninvasive electroencephalography (EEG), to investigate (1) how dogs track speech modulations; and (2) if, like in humans, dogs’ speech tracking accuracy predicts comprehension. Unlike previous studies, e.g., [24,30,49–51], we selected command words as speech stimuli, which allowed us to use dogs’ behavioural responses as an index of “intelligibility,” while remaining within the structural definition of the DDS register, i.e., short (3 words on average), mostly one-node, imperative utterances [18].

## Results

### Natural vocal rate in dogs and humans

**Fig 1:**
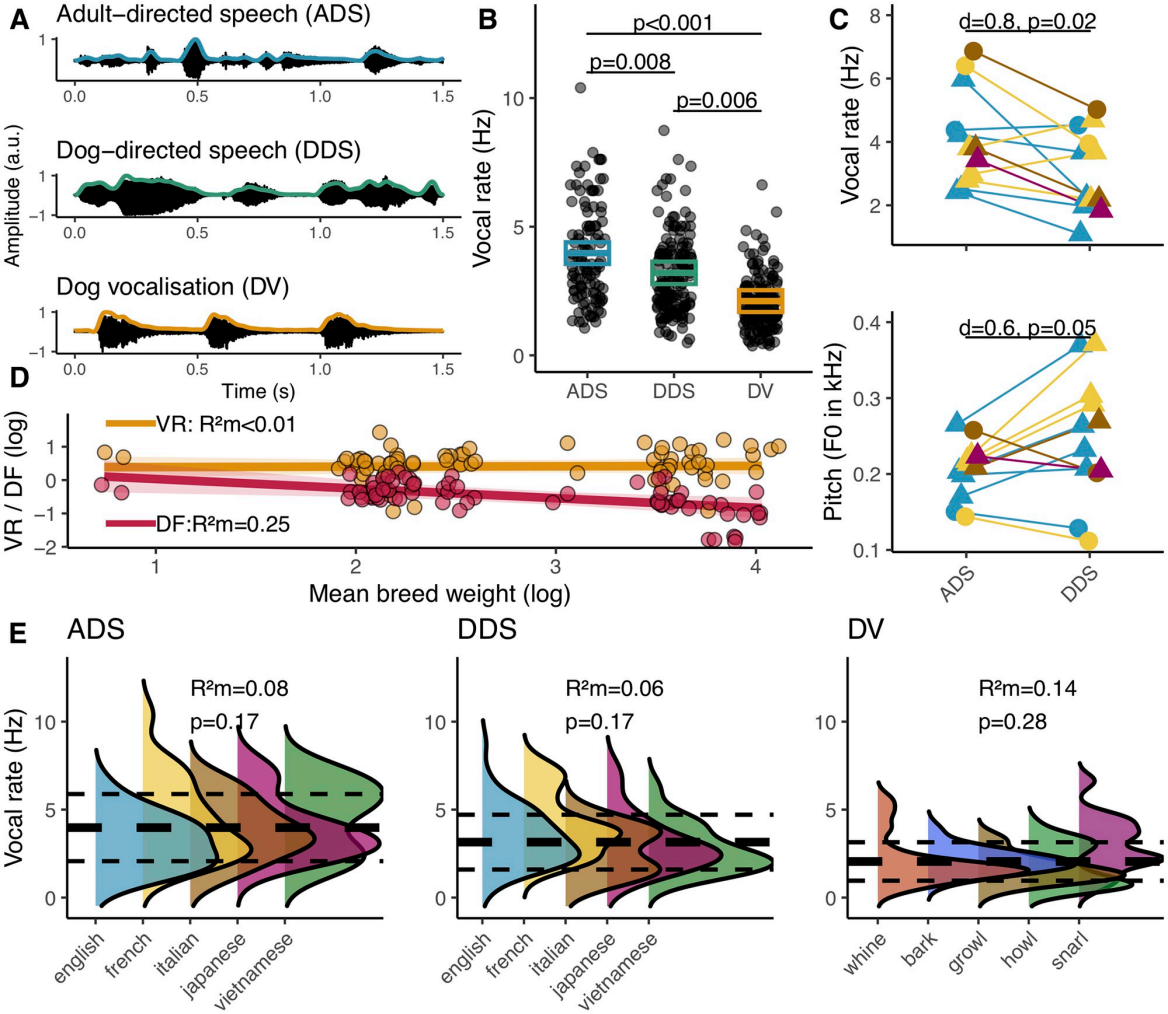
Comparison of dog/human vocal production. (A) Oscillograms and, overlaid, envelopes used to compute the vocal rate. (B) Model estimates and their 95% CI of vocal rate in dog and human sequences. Black dots are the original observations. (C) Vocal rate (Hz) and mean F0 (Hz) for matched ADS and DDS speech sentences. (D) Model slope and 95% CI of weight effect on dog VR and DF. (E) Density distribution of vocal rate according to vocal classes for dogs and languages for humans. Overall mean (thick dashed line) and SD (thin dashed lines) statistics are displayed. See S1 Data for the underlying data. ADS, adult-directed speech; DDS, dog-directed speech; DF, dominant acoustic frequency; VR, vocal rate.

Using 143 vocal sequences (30 dogs) including all major vocal classes (barks, growls, howls, snarls, and whines [15]), 106 adult-directed (27 individuals, 10 women) and 149 dog-directed speech sequences (22 individuals, 16 women) spanning 5 different languages, we found that dogs vocalise at a slower rate than humans (dogs mean ± SD: 2 ± 1.1 vocalisations/s, ADS: 4 ± 1.9 syllables/s; Tukey-corrected post hoc pairwise comparison: t = 6.8, *p* < 0.001, Fig 1A and 1B). We also found that DDS has a slower rate (3 ± 1.6 Hz) than ADS (t = 3.1, *p* = 0.008), but faster rate than the average dog vocal rate (t = 3.9, *p* = 0.006). For a subset of speakers, we found duration-matched DDS and ADS sentences, allowing us to confirm that pet owners slow their speech rate when talking to their dogs (paired *t* test: t = 2.7, df = 11, *p* = 0.02, Cohen’s d = 0.8, Fig 1C). DDS also has higher mean F0 than ADS (t = −2.2, df = 11, *p* = 0.05, Cohen’s d = 0.6) confirming previous results [17,19]. Further analyses of vocal sequences returned no significant differences in vocal rate among vocal classes in dogs (F_4,16.1_ = 1.4, *p* = 0.28) nor among languages in both speech types (F_4,31.8_ = 2.1, *p* = 0.11, Fig 1E). Thus, the dog’s vocal rate is overall slower than human speech and importantly, pet owners modify not only the spectral but also the temporal feature of their output when speaking to their dogs, in a direction that brings them closer to the natural vocal rate of the latter.

Furthermore, when exploring other factors known to influence the structure of animal vocal signals [2], we found no evidence of large inter-individual differences in vocal rate unlike for the dominant acoustic frequency (S1 Table) confirming the latter’s functional significance in individual discrimination [52,53] and speaking against such selection effects in the former. Concurrently, body weight had no explanatory effect on vocal rate variation (F_1,11.41_ = 0.04, *p* = 0.8) while it was inversely related to dominant acoustic frequency (F_1,12.07_ = 6.03, *p* = 0.03) confirming the known acoustic allometric relationship between body weight and spectral parameters [54] and speaking to other types of constraints on vocal rate (Fig 1D) [1].

### Neural tracking and speech “intelligibility”

**Fig 2:**
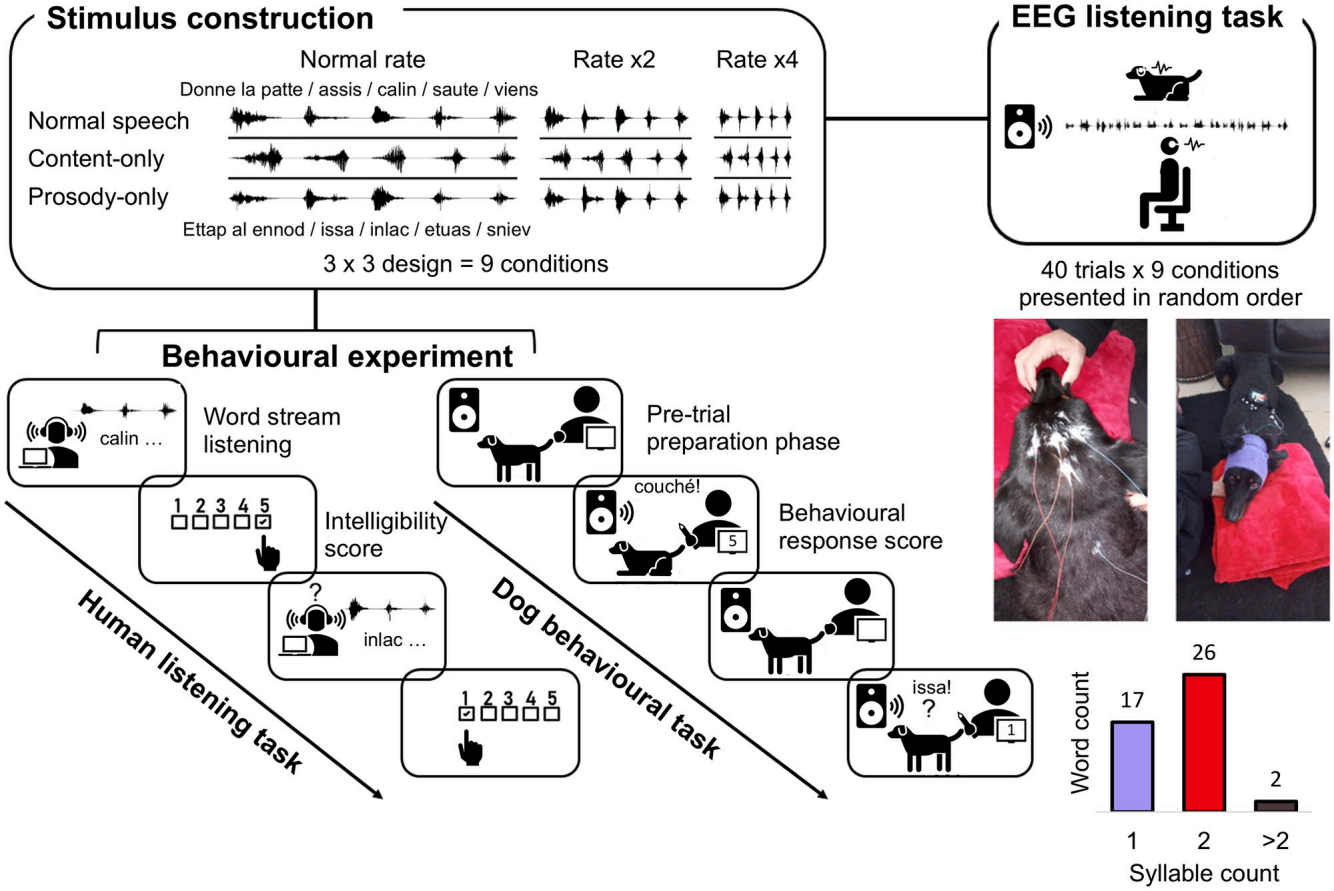
Schematic of the perception study. Word stream stimuli were first constructed by recording dog-specific command words (mostly disyllabic and monosyllabic, cf. small insert) that were appended into a 5-word stream with approximately 300 ± 50 ms silence intervals. These word streams were altered with regards to (1) speech type: by either removing content (reversed words) or prosodic information (flattened pitch modulation and reversed energy contour); and (2) speech rate: compression by a factor of 2 or 4; amounting to 9 word-stream conditions in total. The behavioural experiment consisted of an intelligibility scoring task for humans who listened to the full word stream, and of a playback task for dogs, who heard each word command separately (45 in total) a maximum of 3 times each, while the experimenter and the owner agreed on a behavioural response score. For the EEG experiment, dogs were first fitted with 1 to 4 electrodes covered by a headband and linked to an amplifier strapped on their back (photo inserts). They were then instructed to lie down and passively listen to an audio track (broadcasted via a speaker) containing 40 repetitions of each word stream condition. For comparability purposes, human EEG recordings were made under the same experimental conditions (see also S2 Data). Photo credit: E. Déaux. EEG, electroencephalography.

To investigate auditory neural processes in dogs, we adapted typical human protocols, e.g., [39,41,43], where speech intelligibility is altered using spectral and temporal modifications of speech stimuli and neural tracking strength is correlated to behavioural measures of intelligibility (Fig 2). Speech streams were composed of words that the dogs had learned to respond to, i.e., command words (e.g., “sit,” “come”). We used command DDS rather than praising DDS (e.g., “oh that’s a good boy!”) to be able to obtain an objective index of “intelligibility” in the sense of a successful stimulus–action relationship, assessed during the behavioural task. In humans, comprehension was measured by asking participants to rate word streams on an intelligibility scale. We performed EEG and behavioural experiments on 12 dogs (1 to 13 years old, 7 females) and 12 paired human participants (18 to 65 years old, 6 women) with no self-reported hearing deficits. Four dogs and 1 human participant were excluded from analyses due to poor EEG signal quality.

**Fig 3:**
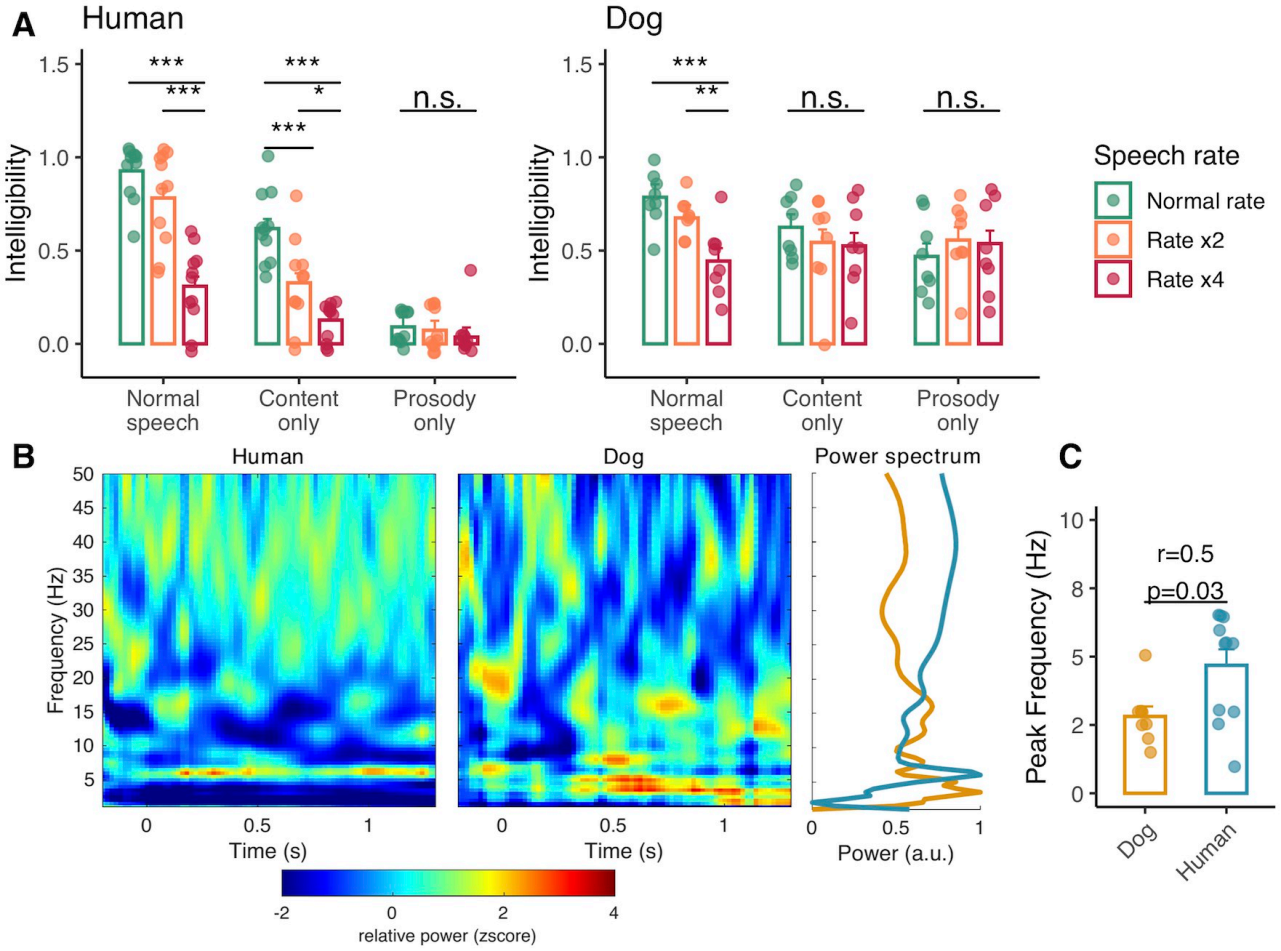
Speech stimulus alteration effects on intelligibility and characterisation of neural responses. (A) Mean (± SE) behavioural responses according to speech type and rate in humans and dogs. Tukey-corrected, post hoc pairwise comparisons are shown. *** *p* < 0.001, ** *p* < 0.01, * *p* < 0.05. (B) Time-frequency plots averaged across all conditions and individuals within species. Z-score transformed relative power is plotted to ease visual comparison across species. (C) Power spectra (peak normalised and averaged between 0 and 1.3 s) and unpaired *t* test between species on frequency of highest power (range = 1–7 Hz). For the underlying data, see S2 Data.

We first confirmed that modifying speech spectral and temporal features altered both species’ perceptual performances. Modifying speech rate (main effect: F_2,80_ = 46.6, *p* < 0.001) and type (main effect: F_2,80_ = 112, *p* < 0.001) affected speech intelligibility in humans, in an interactive way (speech rate by speech type interaction: F_4,80_ = 10.5, *p* < 0.001). When content was removed (i.e., the prosody-only condition), participants failed to understand the speech sentence at all speech rates. In the other 2 conditions, increasing speech rate decreased speech intelligibility (see Fig 3A for post hoc significance pairwise tests). In dogs, speech rate (main effect: F_2,64_ = 4.9, *p* = 0.01) and type (main effect: F_2,64_ = 6.4, *p* = 0.003) also impacted speech intelligibility, again interactively (speech rate by speech type interaction: F_4,64_ = 6.9; *p* < 0.001), with intelligibility dropping as speech rate increased, but only in the normal speech type condition (Fig 3A).

We then quantified the 2 species’ neural responses, restricting the EEG analyses to the FCz electrode in humans as it showed the strongest response to the acoustic stimulation (S1 Fig) and Cz in dogs, known to capture auditory stimulus-locked activity [55,56]. Both dogs and humans showed increased power activity (relative to the pre-stimulus baseline period) in the low frequency range (<10 Hz, Fig 3B), confirming and characterising the auditory cortex activity reported in fMRI studies of dog speech processing [49,50,57]. However, we noted a first difference between the 2 species’ neural responses in this frequency range. Dogs showed a predominant power increase in the delta band (1 to 3 Hz), as opposed to the theta band (4 to 7 Hz) in humans (Mann–Whitney U test: U = 70, df = 17, *p* = 0.03, *r* = 0.5, Fig 3C), speaking to possible divergent auditory processes.

**Fig 4:**
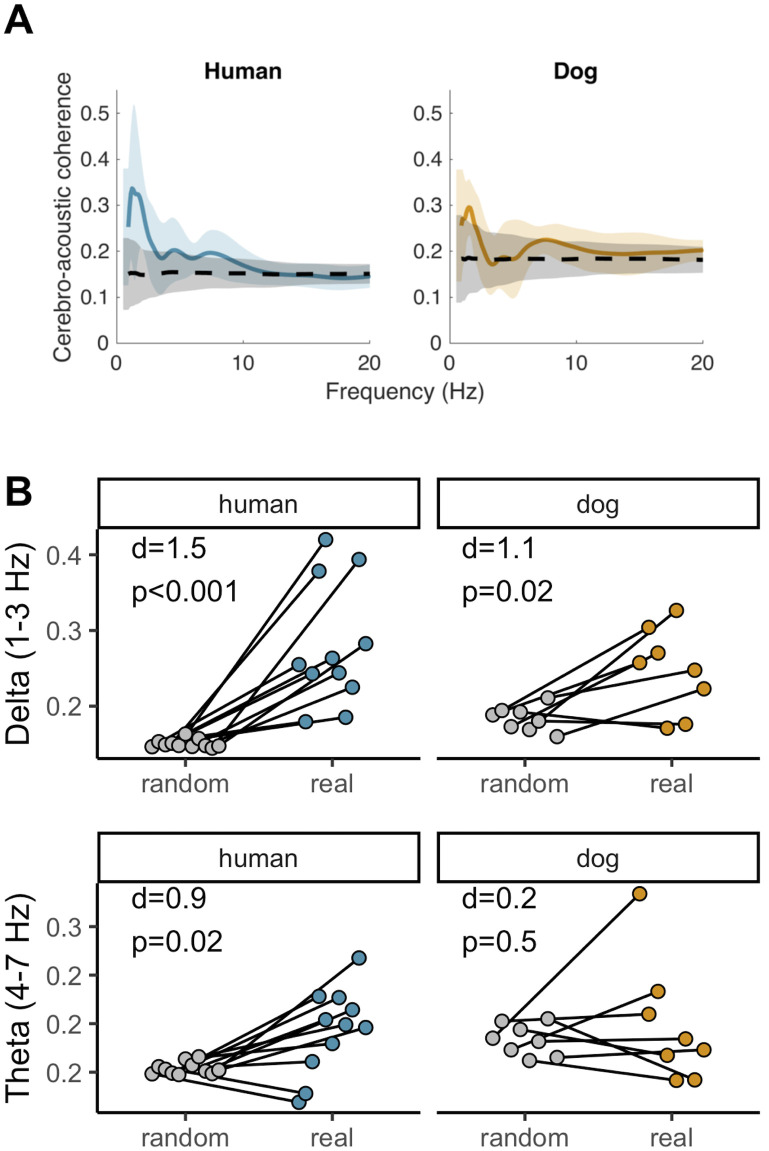
Speech tracking in both species and both the delta and the theta bands. (A) Mean (± SD) cerebro-acoustic coherence over the 1–20 Hz range, calculated from the normal speech condition. Black dashed line shows mean (± SD) random coherence values for pairings of neural signals with randomised acoustic envelopes. (B) Paired *t* test of coherence in the delta and theta range between the real cerebro-acoustic and cerebro-randomised acoustic pairings (see also S3 Data).

Given the presence of a stimulus-related and sustained neural response, we then probed whether dogs display evidence of a speech tracking response under normal speech conditions. Cerebro-acoustic coherence, a measure that quantifies the phase-locking of neural signals to speech envelope [41,42], was above the mean random coherence value throughout the 1 to 10 Hz range in humans, but restricted to a 1 to 3 Hz peak in dogs (Fig 4A). Averaged values in the delta band were significantly higher in the real cerebro-acoustic than in the cerebro-randomised acoustic pairings in humans (paired *t* test: t = −5, df = 10, *p* < 0.001, d = 1.5) and in dogs (t = −3, df = 7, *p* = 0.02, d = 1.1). However, theta cerebro-acoustic coherence was significantly higher than in the cerebro-randomised acoustic pairings in humans (t = −2.87, df = 10, *p* = 0.02, d = 0.9) but not in dogs (t = −0.6, df = 7, *p* = 0.5, d = 0.2). In other words, dogs show evidence of auditory tracking capabilities, as do other species [58–60]; however, in the context of speech stimulation, and unlike in humans, such tracking is restricted to the delta band (Fig 4B).

Having found evidence for a general speech tracking process in both species, albeit at differing oscillation bands, we then focused on determining how flexible tracking was within these frequency ranges and whether, similar to humans, dogs’ cortical tracking was linked to speech intelligibility. As word streams differed among individuals, the specific peak rhythm within each of these frequency ranges (i.e., delta and theta) also differed. As these peak rhythms acoustically represent specific granularity levels (i.e., word versus syllable levels [45]), we first extracted these word and syllable rates and then computed the corresponding cerebro-acoustic coherence value specific to each participant (hereafter referred as to “word cerebro-acoustic coherence” and “syllable cerebro-acoustic coherence”). Indeed, if speech comprehension is linked to tracking strength within these bounded ranges, then as speech rate increases, both cortical tracking and speech comprehension should be negatively impacted. We first confirmed that increasing speech rate had a negative effect on cerebro-acoustic coherence in both species and at both granularity levels (S2 Fig). In humans, both syllable and word cerebro-acoustic coherence decreased as syllable rate (F_1,90.5_ = 9.2, *p* = 0.003, S2A Fig) and word rate (F_1,84_ = 4.2, *p* = 0.04, S2B Fig) increased respectively, but speech type had no effect in either model (Syllable model: F_2,83_ = 0.27, *p* = 0.8; Word model: F_2,83_ = 0.5, *p* = 0.6). The same pattern was found in dogs, with both syllable and word cerebro-acoustic coherence dropping with increasing syllable rate (F_1,65.5_ = 5, *p* = 0.03, S2C Fig) and word rate (F_1,61_ = 9.1, *p* = 0.003, S2D Fig) respectively, while speech type (Syllable model: F_2,59_ = 1.5, *p* = 0.2; Word model: F_2,59_ = 2.9, *p* = 0.07) had no effect.

**Fig 5:**
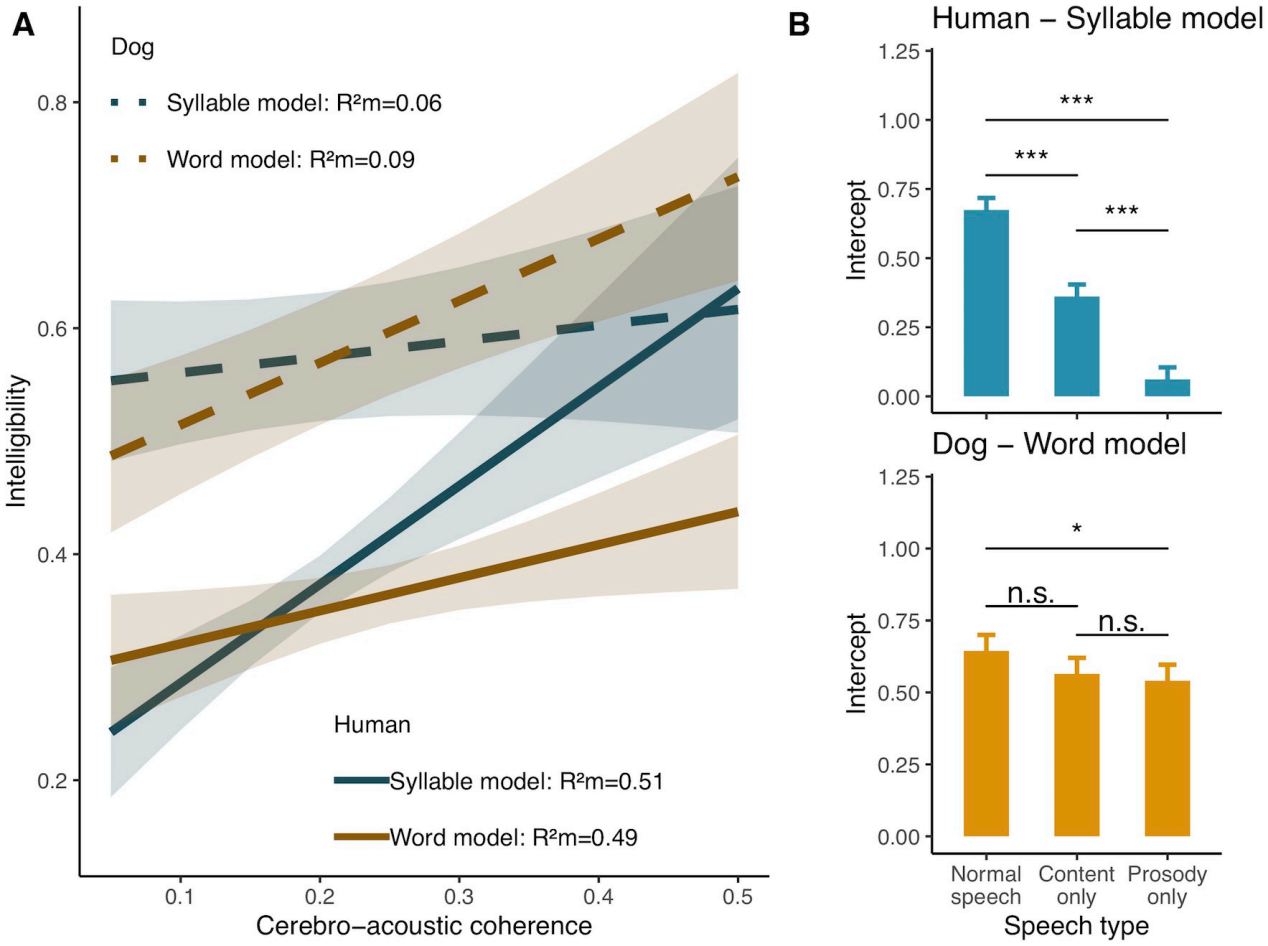
Stronger speech neural tracking (cerebro-acoustic coherence) is linked to increased comprehension. (A) Model slopes and 95% CI for the syllable and word cerebro-acoustic coherence effect on intelligibility in dogs and humans. (B) Mean and SE intercepts for each speech type in humans and dogs, showing that beyond speech tracking, additional processes must be present to explain the differences (see also S2 Data for numerical data).

Remarkably however, the 2 species differed with regards to the granularity level at which tracking was most strongly related to behavioural outputs (Fig 5A). Specifically, in humans, word cerebro-acoustic coherence did not explain intelligibility (F_1,58.3_ = 1.3, *p* = 0.3) while stronger syllable cerebro-acoustic coherence led to increased intelligibility (F_1,89.5_ = 5.5, *p* = 0.02). Conversely in dogs, syllable cerebro-acoustic coherence had no impact on speech intelligibility (F_1,62_ = 0.21, *p* = 0.6), while intelligibility increased with stronger word cerebro-acoustic coherence (F_1,62_ = 4.69, *p* = 0.03). Interestingly, in both species, the speech type (normal, content-only, prosody-only) main effect remained (humans syllable model: F_2,85_ = 49.9, *p* < 0.001; dogs word model: F_2, 61_ = 3.2, *p* = 0.05, Fig 5B), with significant differences among the intercepts of all speech types in humans (all pairwise comparisons: *p* < 0.001) and higher intelligibility in the normal speech condition compared to the prosody-only condition (Norm. speech–Prosody-only est = 0.1, df = 69, t = 2.4, *p* = 0.05) in dogs (all other pairwise comparisons: *p* > 0.05). In other words, like humans, dogs’ comprehension of speech appears to involve more than stimulus-driven auditory processes [45,56,61].

## Discussion

While humans’ main speaking rate is centred around 4 ± 1.9 syllables/s (i.e., theta band) [34,62], we show here that dogs’ vocal rate is slower centred around 2 ± 1.1 vocalisations/s (i.e., delta range). This rate is conserved across vocalisation types, not influenced by body weight and shows only limited inter-individual differences. To ascertain this negative result was not caused by inadequate data, we used the same sample to probe the well-known individual-related differences in dominant frequency and its allometric relationship to body weight (Fig 1) [1,2,63]. These results suggest that despite variation among calls in length and repetition rate, which may convey context-related information, e.g., [64], dogs exhibit a generic ~2 Hz vocal rhythm. While this rhythm differs from the human dominant speech rhythm, these results are consistent with what is known from the speech production system, namely that despite wide linguistic and spectral variations, vocal rates show remarkable consistency [34,62]. It should be pointed, however, that while we tested several important factors known to influence signal variability other variables, such as subject sex or dog breed could not be explored but may yet be shown to also have an impact on vocal rate.

Interestingly, the theta vocal rhythm is also present in many primate species including both closely and more distantly related ones [65–67] leading to hypotheses of an exaptation from masticatory movements [68]. Yet, despite being masticators, dogs do not vocalise in that range and instead exhibit a lower rate, which suggests that the theta rhythm evolved sometime after the split between the Laurasiatheria and Euarchontoglires, and begs for a more thorough characterisation of the phylogeny of the theta rhythm and of the possible selective forces behind its emergence. Nonetheless, although further replication of these results on an independent sample would be welcome, dogs, like humans, appear to be subject to evolutionary factors that have kept temporal production constrained within their species-specific ranges (Fig 1). Such factors could include inherent differences in the function(s) of their respective communication systems [4] and/or, as the results of our perception experiment suggest, neural constraints [69].

We found that dogs exhibit cortical tracking of acoustic stimuli, confirming its nature as a basal processing mechanism [58,59,70]. However, they do not syllabify speech as humans do, but instead primarily track slow amplitude modulations via delta oscillations. Importantly, this slower, word-level tracking does not mean that dogs only process speech via prosodic cues, as we also show that eliciting successful responses requires the full integration of prosodic and content information (Figs 3 and 5). While delta oscillations are also important in human speech encoding and decoding [71], current models of speech perception place the theta-syllable rhythm as the centre of this processing machinery [45,72]. Thus, that dogs do not process speech via theta oscillations but instead primarily rely on delta oscillations, and that this neural rhythm matches their natural vocal rate, supports the hypothesis that dogs’ production/perception tuning is centred around the delta rhythm and has been exapted in the context of human–dog vocal communication. However, what remains to be demonstrated in order to test further this hypothesis is whether delta tracking does indeed support conspecific signal processing. Furthermore, one caveat for these results is that we used concatenated word streams for analytical purposes, and the unnaturalness of the stimulus could have had an impact on dogs’ neural responses. Yet from human research, we know that unnatural stimuli, such as backward speech, suppress tracking [73], such that had the “unnaturalness” of the stimulus strongly affected dogs’ neural responses we would have likely failed to detect cortical tracking.

Importantly, while cortical tracking plays a critical role in speech comprehension in humans [39,41,45,73], this study is the first to establish such a link between neural processes and behavioural outputs in another species. Indeed, while previous research in dog neuroscience has revealed interesting parallels between human and dog neural processes, they typically used speech stimuli that did not allow to objectively measure dogs’ behavioural responses and as such had to assume the functionally adaptive significance of their neural data, e.g., [30,49]. By electing to use commanding DDS (e.g., “sit,” “come”) rather than praising DDS (e.g., “good boy!”), we could objectively quantify stimulus intelligibility and provide conclusive evidence that cortical tracking is a prerequisite of successful acoustic communication not only within but also across species.

Our results additionally lend support to the hypothesis that humans have adjusted their vocal production to exploit the dogs’ neural (auditory) capacity. We find that DDS has a slower rhythm than ADS that brings it closer to the dog’s production rate. That signal design is tuned to exploit receivers’ neural capacities is well established both within species [3] and between species [74] and even professionals across cultures, spontaneously make use of existing sensory biases when training animals [75]. As such, and considering the evolutionary cooperative bond between humans and dogs [13,14], it is plausible that speech temporal tuning to dogs’ sensory systems would have led to more efficient communicative interactions and thus been selected for [76,77]. However, what remains to be established is whether this temporal tuning results from the happenstance of an overgeneralisation of infant-directed speech, possibly due to perceived neotonic traits or limited language abilities [20,78] or truly specific to dogs. Future research could aim to investigate interspecific production and perception tuning between humans and other species, to establish whether humans specifically adjust to the neural capacities of the listener or if it is a more general process. If this vocal adjustment is due to the specific human–dog working bond, we may predict that slow speech should be stable cross-culturally and exhibited even more so in vocal interactions that involve directing working dogs.

One possible limitation of this study is that in the production experiment, DDS consists mainly of sentences (e.g., “what a good boy!”, “wanna play outside?”) while the perceptual experiment used commanding DDS. One could thus argue that they may not have the same acoustic characteristics and thus not be directly comparable. However, when constructing acoustic stimuli, we ensured that the vocal rate would be within the natural DDS range (i.e., 3 ± 1.6 Hz) and complementary analyses revealed no differences in spectral characteristics between the 2 registers (S3 Fig). Furthermore, like previous studies that used praising DDS as stimuli [49,51], we found that eliciting successful responses required the full integration of prosodic and content information (Fig 3). This suggests that commanding and praising DDS may be similarly processed by dogs at least at the auditory level which we explored here. Nonetheless and while it was beyond the scope of this study, it would be greatly beneficial for future comparative work to do a formal acoustic analysis of the 2 types of speech to better understand their similarities and differences and what this may entail for dog perception.

Finally, and for both species, we found that differences in the intercepts among speech types remained even after accounting for the speech tracking effect (Fig 5), suggesting that other processes also influence intelligibility. In humans, cortical tracking is a part of a complex bottom-up and top-down hierarchy known to contribute to comprehension [45,79–81]. Most notably, the hierarchical phase-amplitude coupling of theta and gamma frequency bands allows for phoneme encoding [38,82] while top-down processes involving motor cortex activity [83] are causally related to perception [84]. Such processes have not yet been uncovered in dogs, but have been described in primates [85–88]. Additionally, a recent study in dogs mentioned differential activation in a cortical premotor region when comparing familiar versus unfamiliar language processing, hinting at a possible top-down process [61]. Thus, it will be greatly interesting for future research to investigate whether and to what extent hierarchical bottom-up and predictive processes initially linked to within-species acoustic processing can adapt to or constrain interspecific communication.

Overall, these results reveal that dogs’ auditory and vocal systems have aligned on a single temporal processing window that differs from that of humans, and which remains predominant even when dogs process and appropriately respond to human speech. In parallel, we show that humans who speak to their dogs adopt a speech rate that differs from adult-directed speech and more closely aligns with the dog’s neural delta oscillatory capacity. These data are consistent with the hypothesis that in the history of the dog-human relationship, the neural constraints of the dogs’ reception system may have limited this heterospecific communication to a temporal structure falling midway between the natural speech rate and a slower rate that would perfectly match the dog’s analysis capacity. However, future research on different animal-directed speech registers particularly as it pertains to their temporal characterisation is needed before we can fully establish the extent to which humans are sensitive to their target’s perceptual constraints and whether the dog–human relationship is as special as it may appear.

## Methods

### Ethics statement

All the dogs used in this study were pet dogs who lived with their caregivers. As the tests took place in France and involved noninvasive EEG recordings and behavioural tests, no ethical approval was required under the French law. The human participants all provided informed written consent prior to the experiments and the procedures were approved by the ethics commission of Geneva University CUREG.202011.18.

### Subjects

**Dogs**: Dog owners were recruited by contacting canine clubs located in France. After initial contact with potential participants, dogs were recruited if, based on their owners’ reports, they met the following inclusion criteria: being 1 year or older, having no hearing deficits, a good sociability level, high trainability, and a good level of education. This recruitment process resulted in a pool of 12 dogs (7 females) aged 1 to 13 years old being included, all being medium to large dog breeds, the smallest being Shetland sheepdogs and the biggest being the Beauceron. While all dogs had had basic obedience training, their primary canine sport varied. Half of the dogs were doing obedience training, 4 participated in dog dancing classes and the last 2 animals were training in mantrailing.

**Humans**: We also recruited the same number of human participants (6 women) from the clubs who served as paired controls. Inclusion criteria for human participants were: being aged 18 to 65 years old, having no self-reported hearing deficit, no psychiatric or motor disorders, and speaking French fluently.

### Procedure

#### Perception experiment

**Preexperiment dog training**: We developed a training protocol using positive reinforcement and behavioural shaping to condition dogs to wear the EEG equipment while remaining still. First, dogs were clicker-trained to lay down while resting their head. Once dogs could maintain this position for at least 15 s, they were habituated to wear a headband (happy hoodie, Zony Pets, United States of America) normally used during toileting, to which we made holes to let the ears out (Fig 2, photo inserts). Then, they were finally conditioned to maintain the position, while wearing the headband and listening to a variety of noises including music, environmental noises, and voices. Dogs were judged sufficiently experienced once they could maintain this position regardless of noise or other environmental disturbances for at least 15 s. Throughout the training, dogs were monitored for signs of stress, based on the well-known behavioural markers: yawning, excessive panting, and/or lip licking, as well as body and ear positions. Had a dog exhibited these signs, their training would have been stopped and they would have been excluded from the study.

**Acoustic stimuli**: Typically, comprehension is assessed in humans by asking participants to rate word sequences on an intelligibility scale. As this was not possible for dogs, we selected words that the dogs had learned to respond to, i.e., command words (e.g., “sit,” “come”), allowing us to use behavioural responses to these words as an index of “comprehension” in the sense of a successful stimulus–action relationship. For each dog, we recorded 5 command words spoken by their owners during a typical training session to obtain original, naturalistic DDS. The words were a mix of mono- and disyllabic words (Fig 2). Each dog listened (EEG task) and responded (behavioural task) to their specific set of command words. Their matched control human participant also listened to the same stimuli. Recordings were made with a Sennheiser ME64 microphone and a K6 module mounted onto a FOSTEX FR-2LE field recorder in 44.1 kHz—16 bit wav format. One exemplar of each command word was selected based on the sound quality and on whether that occurrence resulted in a clear, successful behavioural response. The selected command words were first high-pass filtered at 100 Hz, independently normalised at −2 dB and then concatenated into 1 word stream with 300 ± 50 ms silent intervals in between command words. The decision to concatenate command words was methodological, in the sense that to perform the coherence analysis (see the cerebro-acoustic coherence section below) long stimuli were required (at least 1 s). As a control analysis, we used a Kruskal–Wallis test to compare the mean F0 (H = 9.6, *n* = 8, df = 2, *p* = 0.008) and interquartile range (IQR) of F0 (H = 12.2, *n* = 8, df = 2, *p* = 0.002) of the command streams to French ADS and DDS registers. Post hoc Dunn tests with a Bonferroni correction confirmed that for both measurements, there was no significant difference between the command stream and DDS (*p* > 0.05), while ADS had significantly lower mean and IQR F0 than the other 2 speech registers (*p* < 0.05, S3 Fig).

We then used PRAAT and the VocalToolkit plug-in to construct the acoustic stimuli. In total, we constructed nine-word stream stimuli using a fully crossed design of the 3 levels of speech type and the 3 levels of speech rate (Fig 2). In the Content-only condition, we first changed the pitch median of the original dog-directed word sequence to match that of the owner’s adult-directed speech pitch, and to remove all pitch modulations. In a second step, we altered the intensity component of prosody, by reversing the natural intensity contour, while keeping the speech forward. To create the Prosody-only condition, we reversed each individual word rendering the speech unintelligible, while keeping their order in the word stream. Because this process also reversed pitch modulation and intensity, we then copied the pitch and intensity modulation patterns from the original speech word stream, effectively reinstating the original prosody. Finally, given that these procedures resulted in undesired contingent effects, such as slightly robotized voice effects, we also created a control Normal speech condition by first making the pitch monotone and recopying the original pitch contour from the original recording. This ensured that these contingent effects were also present in the Normal word stream and thus controlled for.

To accelerate the speech rate, we used the “change tempo” function in Audacity, https://audacityteam.org/, which accelerates the rate without impacting the pitch. Each stream was compressed by a factor of 2 (twice as fast) and a factor of 4 (4 times as fast). This resulted in a total of 9 word streams to which we affixed the dog’s name in its original form, as a way of capturing the dog’s attention throughout the experimental session. We then created experimental tracks that included all 9 word streams repeated 40 times each, presented in a random order and separated by an inter-stimulus silent interval of 1.5 ± 0.5 s. Experimental tracks lasted on average 23 min.

**Experimental location**: All tests took place at the owner’s home whenever possible, or at another place familiar to the dog (such as another participant’s house belonging to the same canine club). This avoided having to familiarise animals with new locations and gave us more flexibility during the COVID-19 situation. Typically, this involved using the living room area of the house, with the dog being either positioned on a bedding or a couch, depending on its usual place during the preexperiment training.

**EEG listening task**: On the day of the EEG test, dogs were fitted with 1 to 4 golden cup electrodes (at least Cz and if possible, C3, C4, and POz) using gel and a conductive paste, and connected to a g.Nautilus amplifier (g.tec medical engineering GmbH, Austria) secured on the back of the dog, which wirelessly transmitted data to a receiver connected to a recording DELL laptop. For 8 dogs (out of the 12), only 1 electrode could be positioned, such that we selected Cz as it is the most reliably located [89] and is known to show acoustic stimulus-locked responses [55], as our results confirmed. The reference electrode was placed at the nap of the neck (Fig 2, photo inserts). Electrodes were then secured by the headband to prevent any movement during the experiment. Electrode impedance was kept under 30 kΩ and data were recorded at a 500 Hz sampling rate. Dogs laid down facing a PREMIO 8 speaker (T.A.G Montpellier, France) placed 2 m away. The experimental track was then broadcast at 60 ± 5 dBC. The experiment was paused regularly to reward the dog for maintaining the position or when the dog became restless. On average the dog EEG listening task lasted 39.6 ± 15.8 min.

Human recordings were made as similar as possible, using the same recording device and the same set-up. The only differences being that we used 7 to 8 gel-based g.SCARABEO (g.tec medical engineering GmbH, Austria) active electrodes (FCz, AFz, CP3, CP4, CPz, FC3, FC4, and POz) inserted in a cap and ear-referenced, and that the participants were asked to sit in a chair and instructed to avoid movements and blinking during the stimulus presentation. No breaks were given during the presentation.

### Behavioural task

**Humans**. Participants were asked before the EEG listening task, to score the linguistic material. For that, they were equipped with headphones and listened to each stimulus and were prompted to score on a scale of 0 to 5 how many words they understood. The word streams were randomly ordered but only presented once to avoid learning effects.

**Dogs**. To obtain a comparable index for dogs, we used a playback experiment where dogs were made to listen to each command word separately (45 words in total) and scored on how well they responded to the command. To do so, we installed the speaker at the mouth level of the dog owner, who stood quietly next to it while wearing sunglasses and a face mask, holding their arms along their body or behind their back. This procedure ensured that the experiment was as realistic as possible while preventing dogs from using visual cues to answer the command. Prior to each command word, the dog was positioned in front of the speaker 1 to 2 m away in a position that allowed it to display the appropriate behavioural response (e.g., standing up if the next command was a “sit”). Each command word was played a maximum of 3 times with 10 s of silent interval in between. The first time the word command was played, it was preceded by the name of the dog, to grab her attention and replicate typical training settings. After the command word was played, we scored on a scale of 1 to 5 how accurately the dog responded to the command (Table 1). If the dog obtained a score of 4 or 5 (i.e., perfect response within the 10 s scoring interval), she was rewarded with her usual treat, the playback series for that command was stopped and we moved on to the next command word series, again mimicking a typical training session. Scoring was performed by the experimenter and the dog owner. If the two disagreed, the lower response score was given. If the dog became restless and/or inattentive, the experiment was interrupted by a play and/or walk session and then resumed. On average, the task lasted 53.5 +/− 20.3 min.

**Table 1:** Scoring scale for dogs’ behavioural responses to command words.

Score	Behavioural response
**5**	Complete response within 5 s
**4**	Complete response after 5 s and before 10 s
**3**	Incomplete response
**2**	Nonspecific or wrong response
**1**	No response within 10 s

### Production experiment

**Dogs**. We collected vocal sequences from YouTube videos using the freely available Audio Set database [90]. A total of 143 sequences (30 individuals) lasting >1.5 s were extracted, spanning the range of basic vocalisations in canids: barks (*n* = 54, 38%), growls (*n* = 18, 13%), whines (*n* = 21, 15%), snarls (*n* = 17, 12%), and howls (*n* = 33, 23%) [15]), with vocalisation classification being done by ear. If the sequences contained more than 1 vocal type, i.e., were “mixed sounds” (which concerns approximately a third of the recordings), we classified these according to the vocalisation that was most present in the sequence. Inter- and intra-observer reliability in vocal classification was assessed using the Kappa measure, based on a random sample of 50 recordings (approximately a third of all recordings). The values obtained, i.e., inter-observer (between 2 researchers, ED and TP) Kappa = 0.8 and intra-observer (with a 6-month interval between the 2 classifications) Kappa = 0.9 showed very high reliability in both cases.

We categorised the dogs according to their body size as either small (a terrier-like dog or below) or large and to their age class (adult versus juvenile). Whenever available, we recorded the breed of the dog and obtained the corresponding mean breed weight using the American Kennel Club website (https://www.akc.org/). For the Cane corso, data were unavailable on the AKC website, so we used the French equivalent, the Societé Centrale Canine website (https://www.centrale-canine.fr). Finally, for those 3 individuals whose breed was known and who were pups, we first estimated the age (in months) of the pup from the video and then used the weight curve of the corresponding weight category provided in [91] to obtain the mean weight (50% centile) at that age.

**Humans**. To keep data sets as comparable as possible, we extracted ADS and DDS sequences from YouTube videos (ADS: 106 sequences, 27 individuals, 10 women; DDS: 149 sequences, 22 individuals, 16 women). We selected speech sequences from 5 different languages: English, French, Italian, Japanese, and Vietnamese to cover the range of stress-, syllable-, and mora-timed speech patterns. DDS sentences included both typical praising and command utterances. For 12 individuals (9 women) that produced DDS sequences, we were able to match 1 DDS and 1 ADS exemplar (matched for duration), either extracted from the same video or by looking at other videos published by that user. For this analysis, we were not able to find matching ADS and DDS sequences in Vietnamese.

### Measurements

#### Perception experiment

**Intelligibility index**: For humans, the intelligibility score corresponded to the proportion of correctly comprehended words. To obtain a comparable index for dogs, we calculated the mean response score from the maximum behavioural score obtained in response to each command word of a given condition (Table 1), and then scaled this variable between 0 and 1.

**Audio signal**: We computed the speech envelope (from the onset of the first command word) using the Hilbert transform, low-pass filtered below 30 Hz using an eighth order Butterworth filter, to extract, for each participant, the word and syllable rate for each of the 9 word streams from the power spectrum of the envelope. These word and syllable rate variables were then z-scored and subsequently used as regressors in the statistical analyses investigating the relationships between neural, acoustic, and behavioural data.

**EEG data**: All EEG preprocessing steps were done in MATLAB using the fieldtrip toolbox [92] and custom-written scripts. EEG data were bandpass filtered between 1 and 70 Hz and a DFT filter was applied at 50, 100, and 150 Hz. Signals were then epoched from 1 s pre-stimulus onset to the end of the word sequence. Human data were re-referenced to average and an independent component analysis (ICA) was used to remove eye blink data. ICA was not used on dog data, as for most subjects (8 out of 12), we only had the Cz recording electrode. Artefact rejection (eye blinks, muscle, and jumps) was automatically done using fieldtrip functions, with species-specific cut-off z-values (more stringent for humans). A final visual inspection of all trials was used to remove any other trial that failed the rejection procedure. During these initial procedures, we had to exclude 4 dogs and 1 human participant due to poor signal quality, leaving 8 dogs and 11 humans for the analyses. On average 27.35 ± 5.48 trials were kept per condition in dogs and 35.64 ± 2.84 trials in humans (S2 Table and S4 and S5 Figs).

#### Electrode selection

For dogs, the EEG analyses had to be restricted to the Cz electrode, as it was the only one available for most of them. Thus, we decided to similarly restrict further data analyses to one electrode for humans. To select which electrode to keep, we used a decoding approach, using the mTRF model [93]. Briefly, mTRF models use regularised linear regression to find the latent relationships between the stimulus features (in our case the speech envelope) and the neural response. We ran mTRF models for each subject and each electrode separately, restricting the shifting lag from 100 ms pre-stimulus onset to 500 ms post-stimulus onset. We then calculated the correlation between the reconstructed and the actual stimulus and saved the mTRF *r* value obtained as our measure of how well each electrode responded to the task. A linear-mixed model with electrode as a fixed effect and subject ID as a random term, followed by post hoc analyses showed that FCz had a significantly higher correlation value compared to the other electrodes, and was thus selected for further analyses (S1 Fig).

#### Cerebro-acoustic coherence

To assess the extent of cortical phase-locking to the speech temporal structure, we used the cerebro-acoustic coherence index. Focusing on the control normal speech condition, we first obtained the cross-spectral density between neural signal and the speech envelope using a wavelet method between 1 and 20 Hz in 0.1 Hz frequency steps and 0.01 ms time steps, from 0.6 s post-stimulus onset to 1.3 s. This time window was selected to exclude ERP components resulting from the first word of the sentence, which was always the dog’s name, and to allow keeping trial length equal across subjects. We then used the coherence function in fieldtrip to compute the phase coherence between the speech envelope and neural signal. To evaluate how well subjects tracked the speech signal, we compared the actual coherence to random coherence values obtained from the pairings of neural data with randomised acoustic envelopes averaged over 100 runs. To further characterise neural tracking in the 2 most relevant auditory frequency bands, i.e., delta and theta bands, we extracted mean coherence values (delta: 1 to 3 Hz; theta: 4 to 7 Hz) in both real and random data sets and compared them using paired *t* tests. Then, to explore how tracking was influenced by speech type and rate and how it related to behavioural data, we calculated, for each subject in each condition, the mean word and syllable cerebro-acoustic coherence (time window: 0.6 to 1.3 s post-stimulus onset, time steps: 0.01 s, frequency steps: 0.5 Hz) value centred around the subject-specific stimulus word and syllable rate (+/− 0.5 Hz).

#### Production experiment

**Vocal rate and dominant acoustic frequency**. Acoustic analyses were performed using the seewave package in R [94]. To extract the peak vocal rate, i.e., the predominant rhythm at which vocalisations in a sequence are produced, we first bandpass filtered the sequence between 0.1 and 10 kHz and then computed the signal’s envelope using the Hilbert transform. This envelope was further low-pass filtered below 20 Hz using a fourth order Butterworth filter and a wavelet method was used to obtain the frequency decomposition of the signal and extract the frequency of the highest peak. This method was used rather than the more traditional approaches that are based on counting call units and/or on inter-call intervals, because it makes no assumption with regards to the underlying process that produces changes in the amplitude envelope and is thus more adequate when looking at acoustic signals that vary greatly in length and structure.

As a control analysis, we also extracted the dominant acoustic frequency of one vocalisation per sequence (selected based on its signal-to-noise ratio) for the dogs and the sentence’s mean fundamental frequency (F0) for humans. For the dogs, the vocal unit was first bandpass filtered between 50 Hz and 2 kHz, and the averaged frequency spectrum was then computed to extract the frequency of the peak amplitude. We focused on the dominant acoustic frequency rather than the fundamental frequency, because the latter is not always quantifiable, particularly in noisy and chaotic vocalisations such as barks. For humans, we used PRAAT (with standard settings) to extract the mean F0 in each sequence, as in human speech, F0 is both easy to compute and a better characterisation of pitch than dominant frequency. Pitch values were visually inspected on the spectrograms before extraction to ensure accurate measures.

**Potential for individual coding (PIC)**. Among the numerous selective pressures that can impact signal structure, one is the need for increased individual recognition [63]. To assess whether such a process could explain dog vocal rate variation, we calculated the potential for identity coding (PIC) index of each vocalisation for both the vocal rate and dominant frequency parameters. We calculated the within- and between-individual coefficients of variation (CVw and CVb, respectively) using the formula for small samples and obtained the feature’s PIC value, which is the CVb/meanCVw ratio, where meanCVw is the mean value of the CVw for all individuals [95].

### Statistical analyses

**Behavioural and EEG data**: To investigate how the experimental conditions influenced neural and behavioural responses, we used linear-mixed models (LMMs). Models always included participant ID as a random term. Fixed effects varied depending on the question being addressed and were always first specified as the full model, then interaction terms were dropped if they did not reach significance using the likelihood-ratio test which is suitable in the hypothesis testing framework [96]. For final models, statistical significance of fixed effects was assessed using F-tests and the Kenward–Roger method of degrees-of-freedom approximation, as it has been shown to be a reliable method when LMMs are balanced [97]. Post hoc pairwise comparisons were Tukey corrected. Visual inspection of plots showed that the normality and homoscedasticity of residuals and random effects assumptions were met in all cases. For full reporting of these models and all other statistical analyses, refer to the S6 Rmd and S7 models tables documents. Unpaired or paired *t* tests, with unequal variance (or their nonparametric equivalent when the normality assumption was not met) were used in comparisons when they were warranted.

**Vocal production data**: We tested for differences in vocal rates between dogs and humans using an LMM with species as a fixed effect and subjects within vocalisation/language types as random effects. We used LMMs to assess whether the acoustic measurements varied with vocalisation/language type adding weight class and subject ID as random terms in dogs, while in humans, the random effects were subject ID and sex. Finally, to assess whether acoustic parameters were allometrically related to body weight in dogs, we first log-transformed the variables and then used an LMM with vocal class and subject ID as random terms. A paired *t* test was used to compare DDS and ADS speech of the same speakers, with the normal distribution and homogeneity of variance assumptions having been met.

All statistical analyses were done in R version 4.2.3 [98] as well as most graphic outputs (except Figs 2, 3D and 4A) and involved the packages: scales [99], lme4 [100], lmerTest [101], Matrix [102], effectsize [103], emmeans [104], MuMIn [105], parameters [106], rstatix [107], sjPlot [108], cowplot [109], ggpubr [110], ggsignif [111], gridExtra [112], ggplot2 [113], ggridges [114], and tuneR [115].

## Supporting information

S1 Fig Mean + SE mTRF r values for each electrode, from the human neural data. The mTRF r values were obtained by first using a decoding model to reconstruct acoustic stimuli from neural data then by correlating this reconstructed acoustic data to the actual stimulus envelope. Thus, higher r values indicate that reconstructed data from that electrode better match the original stimulus. A linear-mixed model using electrodes as fixed effects and human ID as a random term, revealed significant differences among electrodes (F_7,67.1_ = 3.17, *p* = 0.006). Post hoc tests (FDR corrected) were done to compare the mean value of one electrode to the average value of all other electrodes. FCz was the only electrode that showed significantly higher mTRF r values compared to all others (see S4 Data for the corresponding data).
(TIFF)

S2 Fig Effect of speech rate on speech neural tracking (cerebro-acoustic coherence). (A) Slope estimate and 95% CI of syllable rate effect on syllabic coherence in humans for each speech type. (B) Slope estimate and 95% CI of word rate effect on word coherence in humans for each speech type. (C) Slope estimate and 95% CI of syllable rate effect on syllabic coherence in dogs for each speech type. (D) Slope estimate and 95% CI of word rate effect on word coherence in dogs for each speech type. The underlying data can be found in S2 Data.
(TIFF)

S3 Fig Comparison of acoustic characteristics of command, DDS and ADS registers. (A) mean F0 and (B) interquartile range of F0 across the 3 speech types. These analyses are based on 8 participants in each group (2 men and 6 women in all cases). Following a Kruskal–Wallis test, pairwise post hoc significance testing was done using a Dunn test and Bonferroni correction (see S5 Data for data).
(PNG)

S4 Fig Time-frequency plots of evoked power for each dog participant. Data are averaged across all conditions and baselined between −1 s to stimulus onset. Averaged (and SD) evoked power across all 8 dogs is shown in the last panel (see S8 Data).
(TIFF)

S5 Fig Time-frequency plots of evoked power for each human participant. Data are averaged across all conditions and baselined between −1 s to stimulus onset. Averaged (and SD) evoked power across all 11 humans is shown in the last panel (see S9 Data).
(TIFF)

S1 Table Summary statistics of vocal rate (VR) and dominant acoustic frequency (DF) in dog vocalisations. The potential for individual coding (PIC) is a measure that quantifies the ratio between the inter- and the intra-individual coefficients of variation, with values >1 indicating high individual distinctiveness. Typical vocal contexts are provided, although in some cases (e.g., barks, howls) vocalisations can be used in a range of situations spanning the affiliative-agonistic continuum.
(DOCX)

S2 Table Summary statistics of the number of trials remaining after preprocessing EEG data. Data are shown according to subjects and conditions.
(DOCX)

S1 Data Dominant acoustic frequency and speech rate of paired ADS and DDS sentences. (XLSX)

S2 Data Perception experiment data, including speech rates, cerebro-acoustic coherence, and behavioural variables for humans and dogs. (XLSX)

S3 Data Delta and theta cerebro-acoustic data for real and permutated acoustic-neural pairings for both humans and dogs. (XLSX)

S4 Data Results of the mtrf analysis for each electrode and each human subject used to establish which electrode best responded to the auditory stimulation. (XLSX)

S5 Data Mean and interquartile range pitch data for French speakers of adult-directed, dog-directed, and command speech type. (XLSX)

S6 Data Rmd file reporting the statistical analyses. (PDF)

S7 Data Full model results and post hoc analyses for the analyses reported in-text. (PDF)

S8 Data EEG data (time by frequency) for each dog subject. (XLSX)

S9 Data EEG data (time by frequency) for each human subject. (XLSX)

## Abbreviations

ADS: adult-directed speech
DDS: dog-directed speech
EEG: electroencephalography
ICA: independent component analysis
IQR: interquartile range
LMM: linear-mixed model
PIC: potential for individual coding
