# Correction: Dog–human vocal interactions match dogs’ sensory-motor tuning

**DOI:** 10.1371/journal.pbio.3002923

**Published:** 2024-11-19

**Authors:** Eloïse C. Déaux, Théophane Piette, Florence Gaunet, Thierry Legou, Luc Arnal, Anne-Lise Giraud

An additional affiliation is missing for the second and third author. Théophane Piette and Florence Gaunet are also affiliated with the Aix- Marseille Univ, ILCB, Aix-en-Provence, France.

The following information is missing from the Funding statement: This work, carried out within the Institute of Convergence ILCB (ANR-16-CONV-0002), has benefited from support from the French government (France 2030), managed by the French National Agency for Research (ANR) and the Excellence Initiative of Aix-Marseille University (A*MIDEX).
