# Supplementary figures and images for: Dog–human vocal interactions match dogs’ sensory-motor tuning
Source: PLoS Biol. 2024 Oct 1;22(10):e3002789. doi: 10.1371/journal.pbio.3002789 (PMC11444399; doi:10.1371/journal.pbio.3002789)

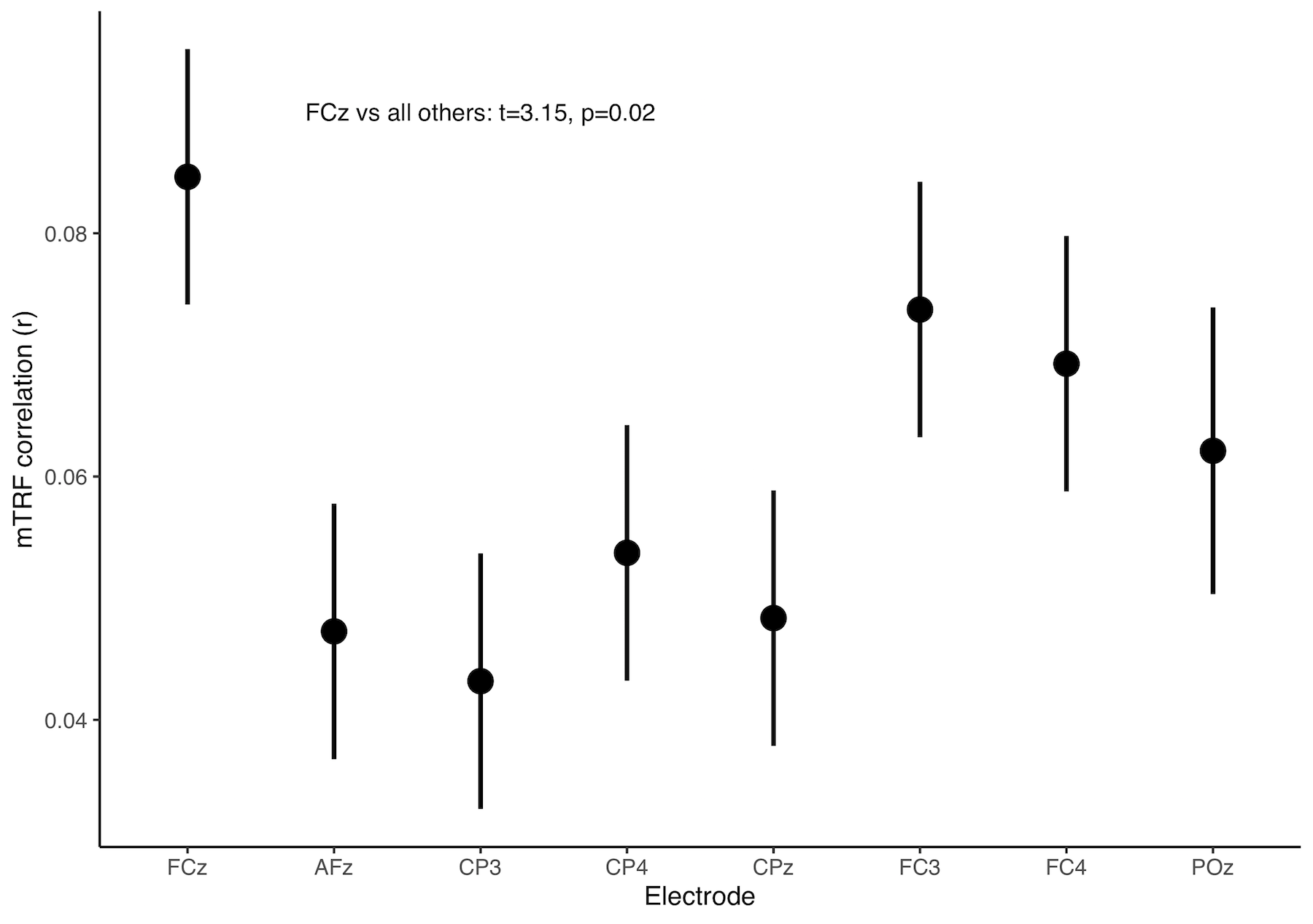

Supplement: S1 Fig — The mTRF r values were obtained by first using a decoding model to reconstruct acoustic stimuli from neural data then by correlating this reconstructed acoustic data to the actual stimulus envelope. Thus, higher r values indicate that reconstructed data from that electrode better match the original stimulus. A linear-mixed model using electrodes as fixed effects and human ID as a random term, revealed significant differences among electrodes (F7,67.1 = 3.17, p = 0.006). Post hoc tests (FDR corrected) were done to compare the mean value of one electrode to the average value of all other electrodes. FCz was the only electrode that showed significantly higher mTRF r values compared to all others (see S4 Data for the corresponding data). (TIFF) [file pbio.3002789.s001.tiff]

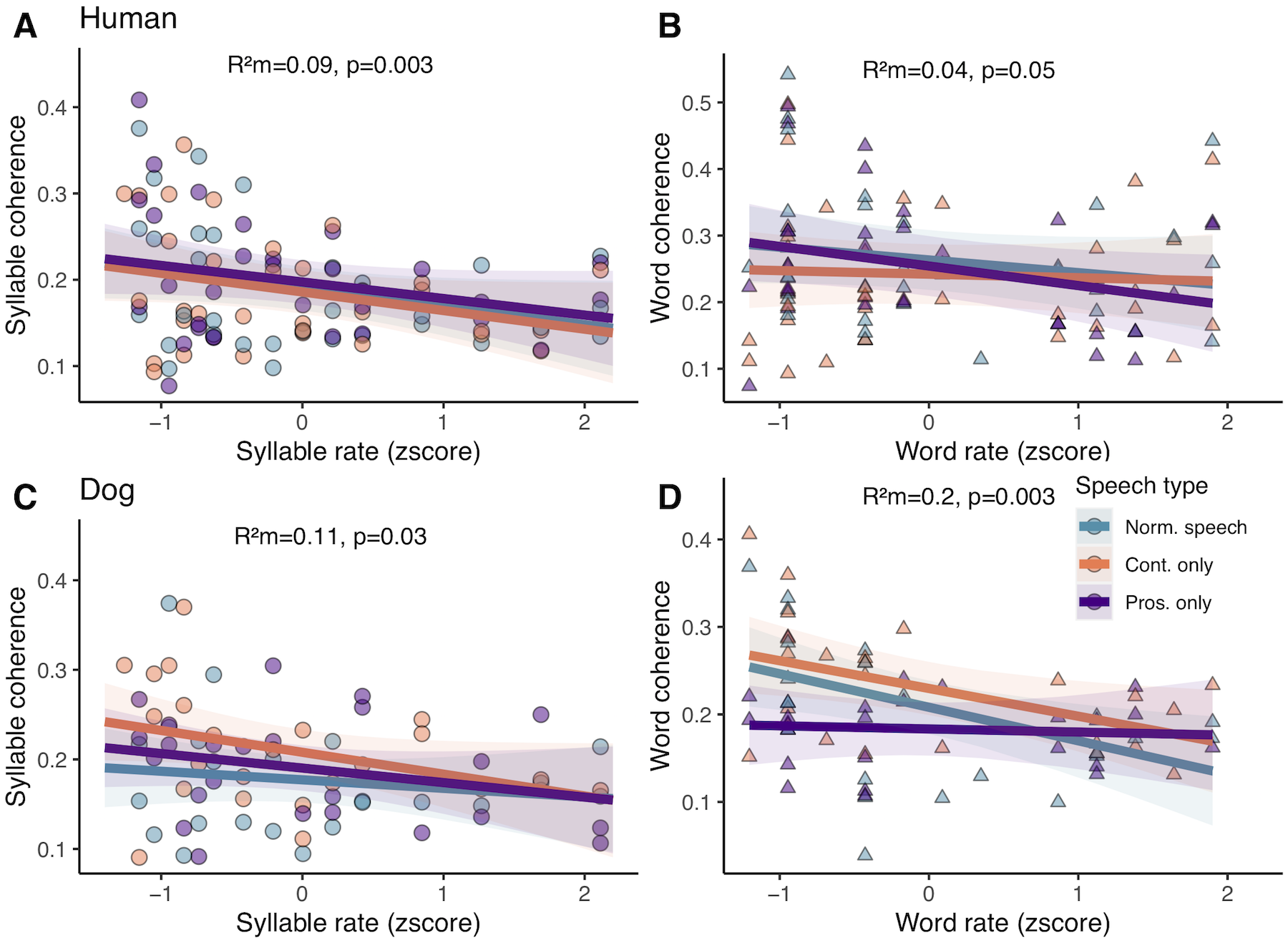

Supplement: S2 Fig — (A) Slope estimate and 95% CI of syllable rate effect on syllabic coherence in humans for each speech type. (B) Slope estimate and 95% CI of word rate effect on word coherence in humans for each speech type. (C) Slope estimate and 95% CI of syllable rate effect on syllabic coherence in dogs for each speech type. (D) Slope estimate and 95% CI of word rate effect on word coherence in dogs for each speech type. The underlying data can be found in S2 Data. (TIFF) [file pbio.3002789.s002.tiff]

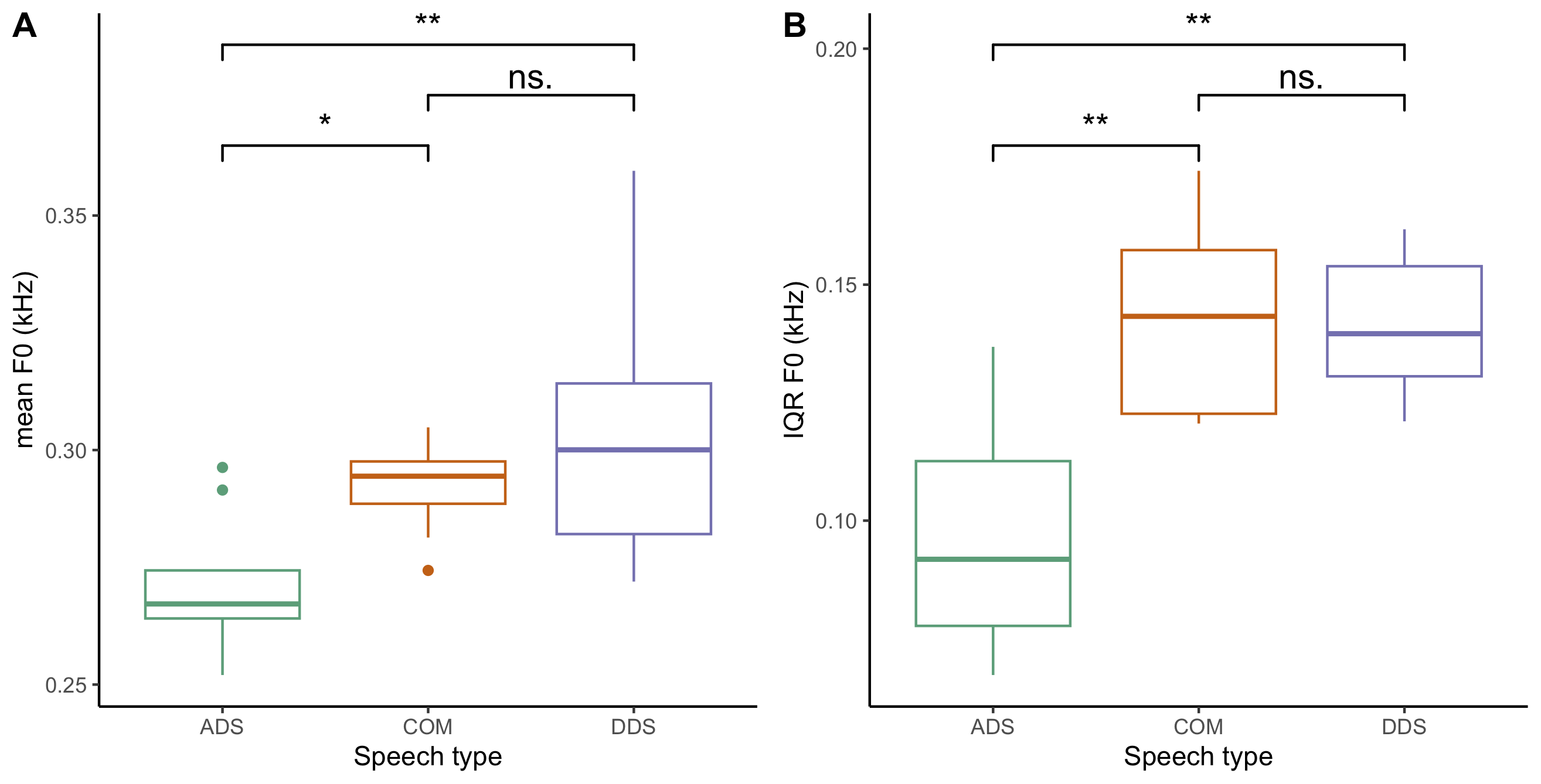

Supplement: S3 Fig — (A) mean F0 and (B) interquartile range of F0 across the 3 speech types. These analyses are based on 8 participants in each group (2 men and 6 women in all cases). Following a Kruskal–Wallis test, pairwise post hoc significance testing was done using a Dunn test and Bonferroni correction (see S5 Data for data). (PNG) [file pbio.3002789.s003.png]

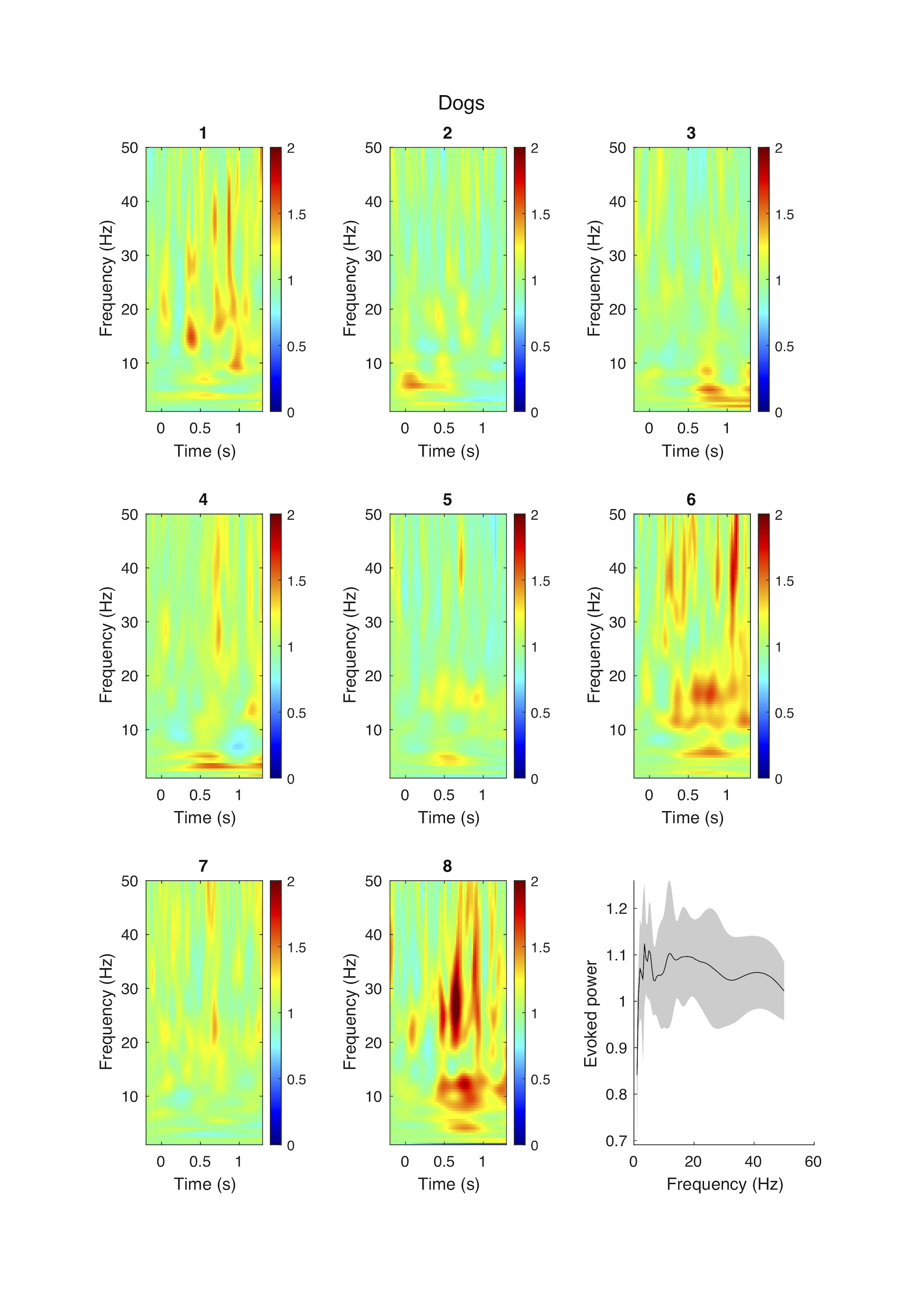

Supplement: S4 Fig — Data are averaged across all conditions and baselined between −1 s to stimulus onset. Averaged (and SD) evoked power across all 8 dogs is shown in the last panel (see S8 Data). (TIFF) [file pbio.3002789.s004.tiff]

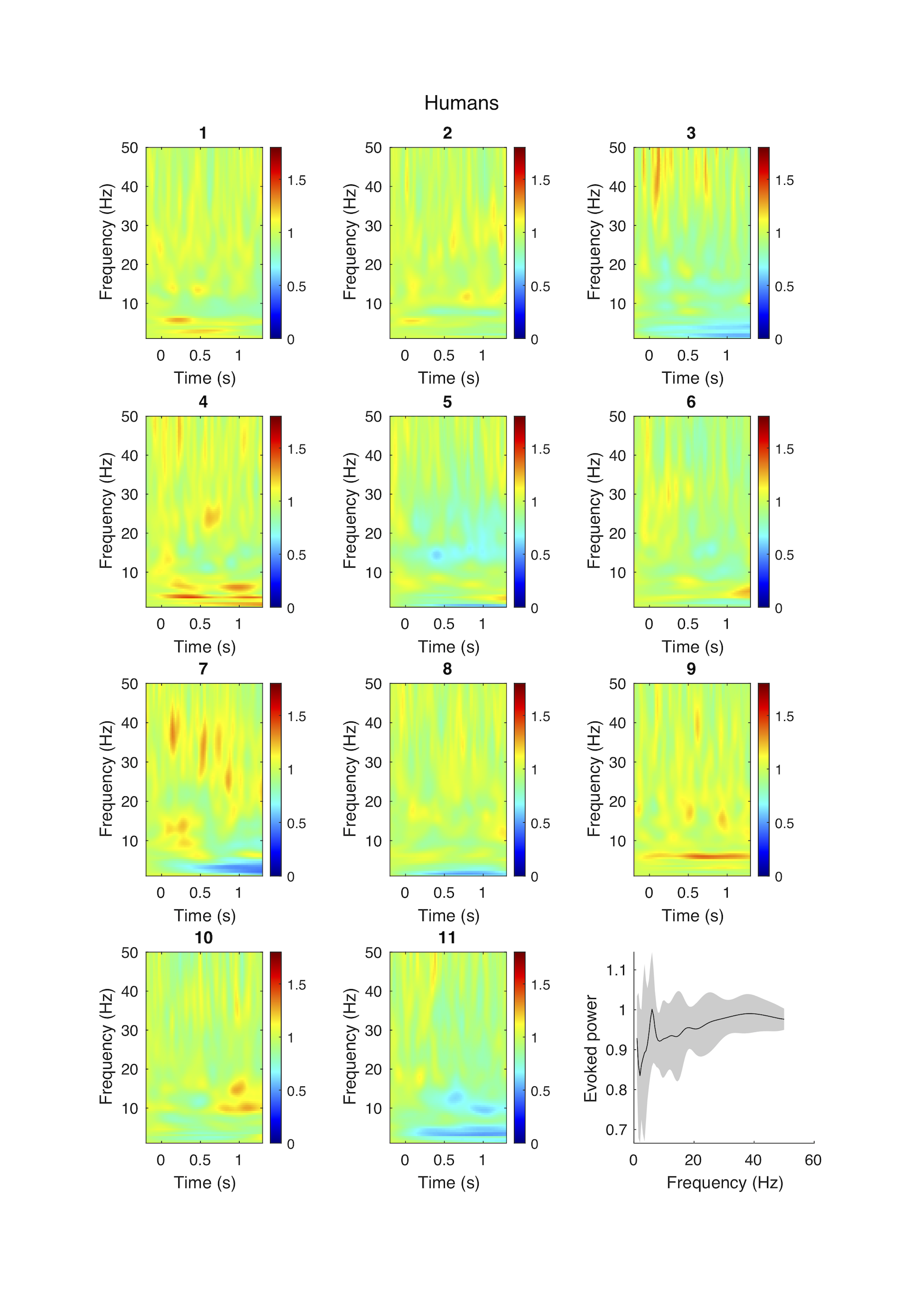

Supplement: S5 Fig — Data are averaged across all conditions and baselined between −1 s to stimulus onset. Averaged (and SD) evoked power across all 11 humans is shown in the last panel (see S9 Data). (TIFF) [file pbio.3002789.s005.tiff]
