# Supplementary material for: Dog–human vocal interactions match dogs’ sensory-motor tuning
Source: PLoS Biol. 2024 Oct 1;22(10):e3002789. doi: 10.1371/journal.pbio.3002789 (PMC11444399; doi:10.1371/journal.pbio.3002789)
Supplement: S1 Table — The potential for individual coding (PIC) is a measure that quantifies the ratio between the inter- and the intra-individual coefficients of variation, with values >1 indicating high individual distinctiveness. Typical vocal contexts are provided, although in some cases (e.g., barks, howls) vocalisations can be used in a range of situations spanning the affiliative-agonistic continuum. (DOCX) [file pbio.3002789.s006.docx]

**Table S1. Summary statistics of vocal rate (VR) and dominant acoustic frequency (DF) in dog vocalisations.** The potential for individual coding (PIC) is a measure that quantifies the ratio between the inter- and the intra-individual coefficients of variation, with values >1 indicating high individual distinctiveness. Typical vocal contexts are provided, although in some cases (e.g. barks, howls) vocalisations can be used in a range of situations spanning the affiliative-agonistic continuum.

| Vocal Class | Context | Mean (SD) VR (Hz) | Mean (SD) DF (Hz) | PIC VR | PIC DF | N |
| --- | --- | --- | --- | --- | --- | --- |
| Bark | Alarm | 2.07 (0.68) | 663 (228) | 1.32 | 2.14 | 54 |
| Growl | Agonistic | 1.62 (0.78) | 502 (288) | 1.07 | 1.48 | 18 |
| Howl | Ambivalent | 1.83 (1.16) | 459 (163) | 1.1 | 1.6 | 33 |
| Snarl | Agonistic | 2.97 (1.55) | 795 (476) | 0.98 | 1.39 | 17 |
| Whine | Affiliative | 1.96 (1.3) | 857 (272) | 1.94 | 1.2 | 21 |
