# Supplementary material for: Dog–human vocal interactions match dogs’ sensory-motor tuning
Source: PLoS Biol. 2024 Oct 1;22(10):e3002789. doi: 10.1371/journal.pbio.3002789 (PMC11444399; doi:10.1371/journal.pbio.3002789)
Supplement: S2 Table — Data are shown according to subjects and conditions. (DOCX) [file pbio.3002789.s007.docx]

**Table S2. Summary statistics of the number of trials remaining after pre-processing EEG data.** Data are shown according to subjects and conditions.

|  | Dogs | | | | | | | | | |
| --- | --- | --- | --- | --- | --- | --- | --- | --- | --- | --- |
| Condition | ID1 | ID2 | ID3 | ID4 | ID5 | ID6 | ID7 | ID8 | Mean | SD |
| Normal R1 | 24 | 20 | 26 | 21 | 30 | 24 | 19 | 29 | 24.13 | 4.05 |
| Normal R2 | 31 | 21 | 32 | 23 | 33 | 30 | 20 | 27 | 27.13 | 5.17 |
| Normal R4 | 35 | 23 | 32 | 29 | 35 | 33 | 27 | 27 | 30.13 | 4.32 |
| Content-only R1 | 27 | 15 | 31 | 23 | 33 | 23 | 12 | 29 | 24.13 | 7.47 |
| Content-only R2 | 33 | 23 | 30 | 23 | 33 | 24 | 20 | 26 | 26.50 | 4.93 |
| Content-only R4 | 33 | 24 | 36 | 27 | 37 | 27 | 25 | 32 | 30.13 | 5.03 |
| Prosody-only R1 | 26 | 23 | 29 | 25 | 30 | 28 | 14 | 33 | 26.00 | 5.76 |
| Prosody-only R2 | 29 | 16 | 31 | 28 | 32 | 31 | 21 | 31 | 27.38 | 5.78 |
| Prosody-only R4 | 30 | 29 | 32 | 30 | 37 | 28 | 25 | 34 | 30.63 | 3.70 |
| Total | 268 | 194 | 279 | 229 | 300 | 248 | 183 | 268 | 246.13 | 41.30 |

|  | Humans | | | | | | | | | | | | |
| --- | --- | --- | --- | --- | --- | --- | --- | --- | --- | --- | --- | --- | --- |
| Condition | ID1 | ID2 | ID3 | ID4 | ID5 | ID6 | ID7 | ID8 | ID9 | ID10 | ID11 | Mean | SD |
| Normal R1 | 37 | 34 | 34 | 36 | 37 | 30 | 33 | 32 | 36 | 37 | 36 | 34.73 | 2.33 |
| Normal R2 | 37 | 32 | 36 | 35 | 38 | 37 | 32 | 35 | 38 | 34 | 38 | 35.64 | 2.25 |
| Normal R4 | 38 | 40 | 33 | 37 | 39 | 37 | 35 | 36 | 37 | 38 | 38 | 37.09 | 1.92 |
| Content-only R1 | 37 | 38 | 35 | 33 | 36 | 38 | 31 | 34 | 34 | 37 | 39 | 35.64 | 2.46 |
| Content-only R2 | 39 | 33 | 32 | 36 | 38 | 40 | 30 | 31 | 35 | 37 | 40 | 35.55 | 3.62 |
| Content-only R4 | 39 | 36 | 36 | 36 | 38 | 37 | 31 | 39 | 36 | 38 | 40 | 36.91 | 2.43 |
| Prosody-only R1 | 38 | 34 | 33 | 36 | 37 | 30 | 28 | 34 | 35 | 35 | 37 | 34.27 | 3.04 |
| Prosody-only R2 | 37 | 37 | 37 | 37 | 38 | 37 | 33 | 29 | 25 | 34 | 38 | 34.73 | 4.22 |
| Prosody-only R4 | 38 | 33 | 35 | 36 | 37 | 38 | 35 | 34 | 34 | 39 | 39 | 36.18 | 2.14 |
| Total | 340 | 317 | 311 | 322 | 338 | 324 | 288 | 304 | 310 | 329 | 345 | 320.00 | 18.63 |
