# Supplementary material for: Dog–human vocal interactions match dogs’ sensory-motor tuning
Source: PLoS Biol. 2024 Oct 1;22(10):e3002789. doi: 10.1371/journal.pbio.3002789 (PMC11444399; doi:10.1371/journal.pbio.3002789)
Supplement: S6 Data — (PDF) [file pbio.3002789.s013.pdf]

Dog-human vocal interactions match dogs' sensory-motor tuning shapes - Reproducible analyses and outputs

E. Déaux

2024-07-25

Load all packages and datasets needed

```
## initialization
require(gridExtra);require(ggplot2);require(emmeans);require(jtools);
require(lme4); require(lmerTest);require(interactions);require(MuMIn);
require(MASS);require(sjPlot);require(ggfortify);library(plyr);require(dplyr)
;
require(reshape2);require(lemon);
require(cowplot);require(ggpubr);require(wesanderson);
library(gridExtra);library(grid);library(extrafont);require(ggeffects);
require(corrplot);require(GGally);require(effectsize); require(scales); requi
re(ggridges);
require(lmtest); require(merTools); require(rstatix); require(parameters); re
quire(ggsignif)
require(png);require(magick)
set.seed(123)

## load the perception dataset
xdata=read.csv('perception_data.csv', stringsAsFactors = T, sep=';')

## load the production dataset
pdata=read.csv('production_data.csv', stringsAsFactors = T, sep=';')

## load the paired ADS/DDS production dataset
padata=read.csv('paired_production_data.csv', stringsAsFactors = T, sep=';')

## load the cacoh real/random dataset
cdata=read.csv('cacoh_data.csv', stringsAsFactors = T, sep=';')
```

Make the necessary changes to the dataset

```
# Change the behavioural response to a proportion and scale covariates

d_bhv=scales::rescale(xdata[xdata$species=='dog',]$bhv_max)
h_bhv=scales::rescale(xdata[xdata$species=='human',]$bhv_max)
xdata$bhv_max_prop=c(d_bhv, h_bhv)

d_bhv=scales::rescale(xdata[xdata$species=='dog',]$bhv_max)
h_bhv=scales::rescale(xdata[xdata$species=='human',]$bhv_max)
xdata$bhv_max_prop=c(d_bhv, h_bhv)
```

```
xdata$syll_rhythm=xdata$syll_rate
xdata$word_rhythm=xdata$word_rate

xdata$syll_rate=scale(xdata$syll_rate)
xdata$word_rate=scale(xdata$word_rate)
```

Check the dataset

```
str(xdata)

## 'data.frame':    171 obs. of  17 variables:
##  $ ID           : Factor w/ 19 levels "D01","D03","D04",..: 1 1 1 1 1 1 1 1
1 2 ...
##  $ condition    : int  1 2 3 4 5 6 7 8 9 1 ...
##  $ dog_name     : Factor w/ 11 levels "bouddha","hakan",..: 1 1 1 1 1 1 1 1
1 3 ...
##  $ species      : Factor w/ 2 levels "dog","human": 1 1 1 1 1 1 1 1 1 1 ...
##  $ Pair         : Factor w/ 11 levels "HD01","HD02",..: 1 1 1 1 1 1 1 1 1 3
...
##  $ type         : Factor w/ 3 levels "CPC","NPC","PNC": 1 1 1 2 2 2 3 3 3 1
...
##  $ rate         : Factor w/ 3 levels "R1","R2","R4": 1 2 3 1 2 3 1 2 3 1 ..
.
##  $ word_rate    : num [1:171, 1] -0.945 -0.169 1.124 -0.945 -0.428 ...
##   ..- attr(*, "scaled:center")= num 3.25
##   ..- attr(*, "scaled:scale")= num 1.89
##  $ syll_rate    : num [1:171, 1] -0.945 -0.207 1.269 -1.05 -0.418 ...
##   ..- attr(*, "scaled:center")= num 7.8
##   ..- attr(*, "scaled:scale")= num 4.63
##  $ bhv_max      : num  4.4 3.8 3 3.8 2.6 2.4 2.4 2.8 2.6 3.8 ...
##  $ CacohS       : num  0.236 0.12 0.172 0.248 0.181 ...
##  $ CacohW       : num  0.32 0.221 0.153 0.316 0.155 ...
##  $ count_resp1  : int  4 3 2 3 1 1 1 2 1 3 ...
##  $ prop_resp1   : num  0.8 0.6 0.4 0.6 0.2 0.2 0.2 0.4 0.2 0.6 ...
##  $ bhv_max_prop: num  0.9 0.75 0.55 0.75 0.45 0.4 0.4 0.5 0.45 0.75 ...
##  $ syll_rhythm  : num  3.42 6.84 13.67 2.93 5.86 ...
##  $ word_rhythm  : num  1.46 2.93 5.37 1.46 2.44 ...

head(xdata)

##    ID condition dog_name species Pair type rate  word_rate  syll_rate bhv_
max
## 1 D01         1  bouddha     dog HD01  CPC   R1 -0.9451034 -0.9449919
4.4
## 2 D01         2  bouddha     dog HD01  CPC   R2 -0.1693329 -0.2071310
3.8
## 3 D01         3  bouddha     dog HD01  CPC   R4  1.1236181  1.2685908
3.0
## 4 D01         4  bouddha     dog HD01  NPC   R1 -0.9451034 -1.0504006
3.8
## 5 D01         5  bouddha     dog HD01  NPC   R2 -0.4279230 -0.4179484
```

```
2.6
## 6 D01         6   bouddha     dog HD01   NPC    R4   1.1236181   0.8469560
2.4
##       CacohS    CacohW count_resp1 prop_resp1 bhv_max_prop syll_rhythm
## 1 0.2358847 0.3196952           4        0.8         0.90    3.417969
## 2 0.1197775 0.2209788           3        0.6         0.75    6.835938
## 3 0.1724252 0.1525846           2        0.4         0.55   13.671875
## 4 0.2480067 0.3163699           3        0.6         0.75    2.929688
## 5 0.1807359 0.1545832           1        0.2         0.45    5.859375
## 6 0.2445663 0.1553423           1        0.2         0.40   11.718750
##   word_rhythm
## 1    1.464844
## 2    2.929688
## 3    5.371094
## 4    1.464844
## 5    2.441406
## 6    5.371094
```

Additional preparations

```
# prep colors for plots
blueposter=rgb(42, 144, 173, maxColorValue = 255)
pinkposter=rgb(194, 34, 73, maxColorValue = 255)
goldposter=rgb(225, 147, 14, maxColorValue = 255)
greenposter=rgb(51, 151, 116, maxColorValue = 255)
purpleposter=rgb(75,0,130, maxColorValue = 255)
orangeposter=rgb(255,127,80, maxColorValue = 255)

# ref level
xdata$species=relevel(xdata$species, 'dog')
xdata$type=relevel(xdata$type, 'CPC')
xdata$rate=relevel(xdata$rate, 'R1')

# labels (for plotting)
type.labs <- c("Norm. speech", "Cont. only", "Pros. only")
names(type.labs) <- c("CPC", "NPC", "PNC")
species.labs<-c('Dog', 'Human')
names(species.labs)=c('dog', 'human')
rate.labs <- c("Norm. rate", "Rate x2", "Rate x4")
names(rate.labs) <- c("R1", "R2", "R4")
```

Dogs’ natural vocal rate

```
str(pdata)

## 'data.frame':    398 obs. of  12 variables:
##  $ Species    : Factor w/ 2 levels "dog","human": 1 1 1 1 1 1 1 1 1 1 ...
##  $ file       : Factor w/ 398 levels "sel.01.adult_howl.wav",..: 171 174 54
262 167 215 306 117 7 281 ...
##  $ voc_type   : Factor w/ 3 levels "ADS","DDS","DV": 3 3 3 3 3 3 3 3 3 3 ..
.
##  $ breed      : Factor w/ 14 levels "akita","Boston Terrier",..: NA 1 NA NA
```

```
10 10 NA NA NA NA ...
##  $ size      : Factor w/ 2 levels "large","small": 2 2 1 2 1 1 2 2 2 NA ..
.
##  $ call      : Factor w/ 10 levels "bark","english",..: 8 1 10 10 1 1 8 8
8 1 ...
##  $ age       : Factor w/ 4 levels "adult","man",..: 1 3 1 1 1 1 NA 1 NA 1
...
##  $ ID        : Factor w/ 78 levels "D1","D10","D11",..: 14 23 19 22 8 8 29
13 29 1 ...
##  $ weight_akc: num  NA 12.5 NA NA 56.2 ...
##  $ peaks     : num  6.63 2.26 4.85 2.69 2.6 ...
##  $ domFreq   : num  0.172 0.603 0.689 0.861 0.345 ...
##  $ dur       : num  1.5 1.51 1.52 1.58 1.61 ...
```

summary statistics of overall dataset

```
pdata=pdata[pdata$dur>1.5,]

VRdog=mean(pdata[pdata$Species=='dog',]$peaks)
VRdog_sd=sd(pdata[pdata$Species=='dog',]$peaks)

VRhumanADS=mean(pdata[pdata$Species=='human' & pdata$voc_type=='ADS',]$peaks)
VRhumanADS_sd=sd(pdata[pdata$Species=='human' & pdata$voc_type=='ADS',]$peaks
)

VRhumanDDS=mean(pdata[pdata$Species=='human' & pdata$voc_type=='DDS',]$peaks)
VRhumanDDS_sd=sd(pdata[pdata$Species=='human' & pdata$voc_type=='DDS',]$peaks
)

print(c(VRdog, VRhumanADS, VRhumanDDS))

## [1] 2.050733 3.977622 3.151335

print(c(VRdog_sd, VRhumanADS_sd, VRhumanDDS_sd))

## [1] 1.093032 1.907069 1.558825
```

Differences dog - human

```
speech_diffVR.lm=lmer(data=pdata, peaks~voc_type+(1|call)+(1|ID))

anova(speech_diffVR.lm,ci_method = "kenward")

## Type III Analysis of Variance Table with Satterthwaite's method
##          Sum Sq Mean Sq NumDF  DenDF F value    Pr(>F)
## voc_type 97.803  48.901     2 9.6536  23.547 0.0001936 ***
## ---
## Signif. codes:  0 '***' 0.001 '**' 0.01 '*' 0.05 '.' 0.1 ' ' 1

m = model_parameters(speech_diffVR.lm, ci_method = "kenward")
print_md(m)
```

# Fixed Effects

Parameter	Coefficient	SE	95% CI	t	df	p
(Intercept)	3.97	0.20	(3.55, 4.40)	20.12	14.10	< .001
voc type [DDS]	-0.77	0.25	(-1.26, -0.27)	-3.11	61.21	0.003
voc type [DV]	-1.86	0.28	(-2.46, -1.26)	-6.77	12.16	< .001

# Random Effects

Parameter	Coefficient
SD (Intercept: ID)	0.48
SD (Intercept: call)	0.22
SD (Residual)	1.44

```
voc.contrast <- emmeans(speech_diffVR.lm, pairwise ~ voc_type)
print(voc.contrast)

## $emmeans
##  voc_type emmean    SE   df lower.CL upper.CL
##  ADS        3.97 0.197 14.1     3.55     4.40
##  DDS        3.21 0.201 12.6     2.77     3.64
##  DV         2.11 0.192 10.4     1.68     2.53
##
## Degrees-of-freedom method: kenward-roger
## Confidence level used: 0.95
##
## $contrasts
##  contrast  estimate    SE   df t.ratio p.value
##  ADS - DDS    0.766 0.246 61.2   3.110  0.0079
##  ADS - DV     1.864 0.275 12.2   6.768  0.0001
##  DDS - DV     1.098 0.278 11.5   3.947  0.0055
##
## Degrees-of-freedom method: kenward-roger
## P value adjustment: tukey method for comparing a family of 3 estimates

visu_plot=plot_model(speech_diffVR.lm, type='diag')
plot_grid(visu_plot[[1]],visu_plot[[2]]$ID, visu_plot[[3]], visu_plot[[4]])
```

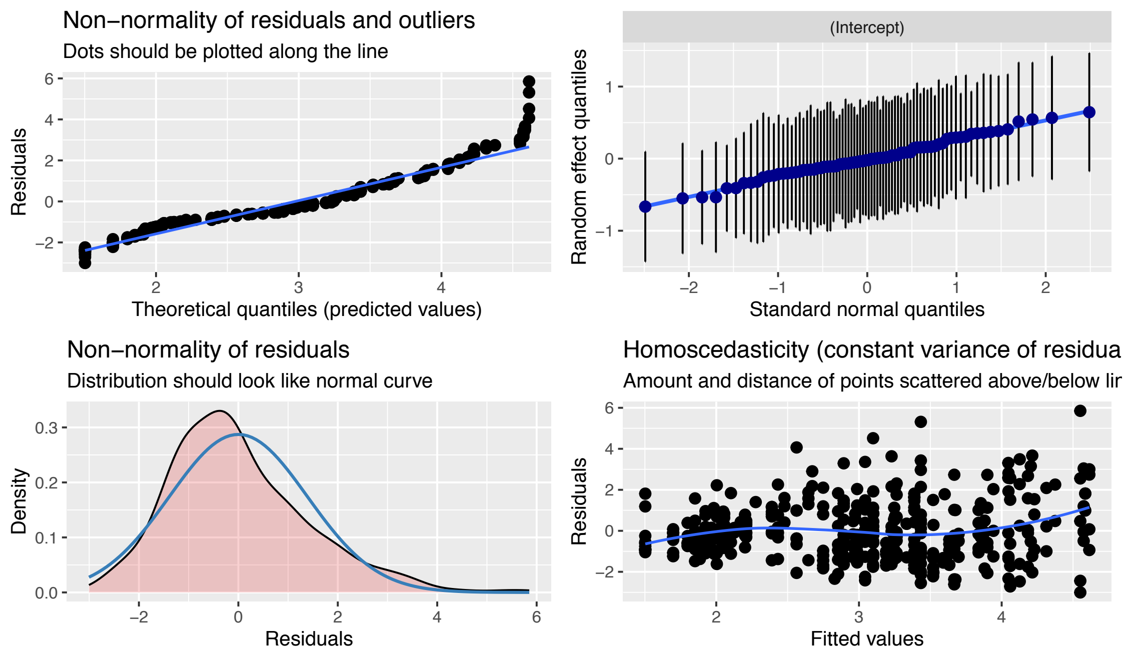


```
r.squaredGLMM(speech_diffVR.lm)

##            R2m       R2c
## [1,] 0.190504 0.2862879

#### paired test ADS - DDS
t.test(data=padata, peaks~voc_type, paired=T)

##
##  Paired t-test
##
## data:  peaks by voc_type
## t = 2.7279, df = 11, p-value = 0.01965
## alternative hypothesis: true mean difference is not equal to 0
## 95 percent confidence interval:
##  0.2015425 1.8852908
## sample estimates:
## mean difference
##        1.043417

cohens_d(data=padata, peaks~voc_type, paired=T)

## # A tibble: 1 × 7
##   .y.   group1 group2 effsize    n1    n2 magnitude
## * <chr> <chr>  <chr>    <dbl> <int> <int> <ord>
## 1 peaks ADS    DDS      0.787    12    12 moderate

t.test(data=padata, F0~voc_type, paired=T)

##
##  Paired t-test
##
```

```
## data:  F0 by voc_type
## t = -2.2049, df = 11, p-value = 0.04966
## alternative hypothesis: true mean difference is not equal to 0
## 95 percent confidence interval:
##  -79.59614649  -0.07052017
## sample estimates:
## mean difference
##       -39.83333
```

```
cohens_d(data=padata, F0~voc_type, paired=T)
```

```
## # A tibble: 1 × 7
##   .y.   group1 group2 effsize    n1    n2 magnitude
## * <chr> <chr>  <chr>    <dbl> <int> <int> <ord>
## 1 F0    ADS    DDS     -0.636    12    12 moderate
```

Call-level analysis

```
# vocal rate
call_peaks.lm = lmer(data = pdata[pdata$Species == 'dog', ], peaks ~ call +
                       (1 | size) + (1 | ID))
m = model_parameters(call_peaks.lm, ci_method = "kenward")
print_md(m)
```

# Fixed Effects

Parameter	Coefficient	SE	95% CI	t	df	p
(Intercept)	2.16	0.29	(1.34, 2.98)	7.45	3.81	0.002
call [growl]	-0.70	0.52	(-1.78, 0.38)	-1.35	18.53	0.192
call [howl]	-0.45	0.59	(-1.69, 0.79)	-0.76	19.04	0.455
call [snarl]	0.85	0.60	(-0.43, 2.13)	1.42	13.97	0.177
call [whine]	0.22	0.48	(-0.81, 1.25)	0.45	14.45	0.658

# Random Effects

Parameter	Coefficient
SD (Intercept: ID)	0.63
SD (Intercept: size)	0.00
SD (Residual)	0.95

```
call_peaks.aov=anova(call_peaks.lm, ddf = 'Kenward-Roger')

a = model_parameters(call_peaks.aov, ci_method = "kenward")
print_md(a)
```

Parameter	Sum_Squares	df	Mean_Square	F	p
call	5.12	4	1.28	1.40	0.279

Anova Table (Type 3 tests)

```
visu_plot = plot_model(call_peaks.lm, type = 'diag')

plot_grid(visu_plot[[1]], visu_plot[[2]]$ID, visu_plot[[3]], visu_plot[[4]])
```

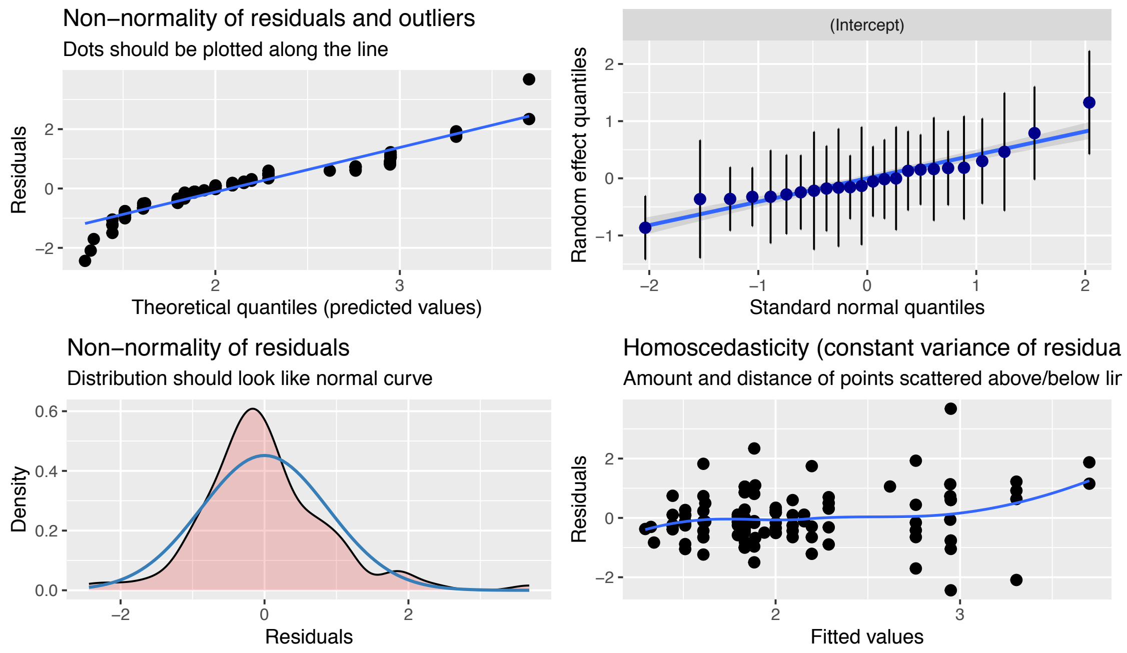


```
r.squaredGLMM(call_peaks.lm)

##             R2m       R2c
## [1,] 0.1420854 0.4034406

# vocal rate in humans as a function of langage
# note: the "age" variable actually refers to sex for humans.

call_peaksHDDS.lm = lmer(data = pdata[pdata$voc_type == 'DDS' | pdata$voc_typ
e == 'ADS' , ], peaks ~ voc_type*call+ (1|age) +(1|ID))

call_peaksHDDS2.lm = lmer(data = pdata[pdata$voc_type == 'DDS' | pdata$voc_ty
pe == 'ADS' , ], peaks ~ voc_type+call+ (1|age) +(1|ID))

anova(call_peaksHDDS.lm, call_peaksHDDS2.lm)

## Data: pdata[pdata$voc_type == "DDS" | pdata$voc_type == "ADS", ]
## Models:
## call_peaksHDDS2.lm: peaks ~ voc_type + call + (1 | age) + (1 | ID)
## call_peaksHDDS.lm: peaks ~ voc_type * call + (1 | age) + (1 | ID)
##                    npar    AIC    BIC  logLik deviance Chisq Df Pr(>Chisq)
## call_peaksHDDS2.lm    9 1006.9 1038.8 -494.46   988.92
## call_peaksHDDS.lm    13 1004.4 1050.4 -489.19   978.39 10.53  4    0.03239
*
## ---
## Signif. codes:  0 '***' 0.001 '**' 0.01 '*' 0.05 '.' 0.1 ' ' 1
```

```r
m = model_parameters(call_peaksHDDS.lm, ci_method = "kenward")
print_md(m)
```

# Fixed Effects

Parameter	Coefficient	SE	95% CI	t	df	p
(Intercept)	3.08	0.45	(1.95, 4.21)	6.86	5.43	< .001
voc type [DDS]	0.40	0.45	(-0.53, 1.33)	0.89	26.70	0.384
call [french]	1.20	0.49	(0.21, 2.19)	2.43	53.17	0.019
call [italian]	0.49	0.64	(-0.78, 1.77)	0.77	62.49	0.443
call [japanese]	0.78	0.50	(-0.22, 1.78)	1.56	58.50	0.123
call [vietnamese]	1.49	0.52	(0.46, 2.53)	2.89	59.88	0.005
voc type [DDS] × call [french]	-0.48	0.77	(-2.02, 1.05)	- 0.63	58.58	0.531
voc type [DDS] × call [italian]	-1.28	0.76	(-2.82, 0.26)	- 1.68	40.90	0.101
voc type [DDS] × call [japanese]	-0.99	0.63	(-2.27, 0.29)	- 1.58	28.64	0.124
voc type [DDS] × call [vietnamese]	-2.08	0.70	(-3.56, - 0.60)	- 2.96	17.16	0.009

# Random Effects

Parameter	Coefficient
SD (Intercept: ID)	0.17
SD (Intercept: age)	0.37
SD (Residual)	1.66

```r
anova(call_peaksHDDS.lm, ddf = 'Kenward-Roger')

## Type III Analysis of Variance Table with Kenward-Roger's method
##               Sum Sq Mean Sq NumDF  DenDF F value  Pr(>F)
## voc_type      11.257 11.2572     1 36.348  4.0671 0.05116 .
## call          22.900  5.7251     4 31.790  2.0607 0.10945
## voc_type:call 26.841  6.7102     4 31.090  2.4147 0.06986 .
## ---
## Signif. codes:  0 '***' 0.001 '**' 0.01 '*' 0.05 '.' 0.1 ' ' 1

visu_plot = plot_model(call_peaksHDDS.lm, type = 'diag')

plot_grid(visu_plot[[1]], visu_plot[[2]]$ID, visu_plot[[3]], visu_plot[[4]])
```

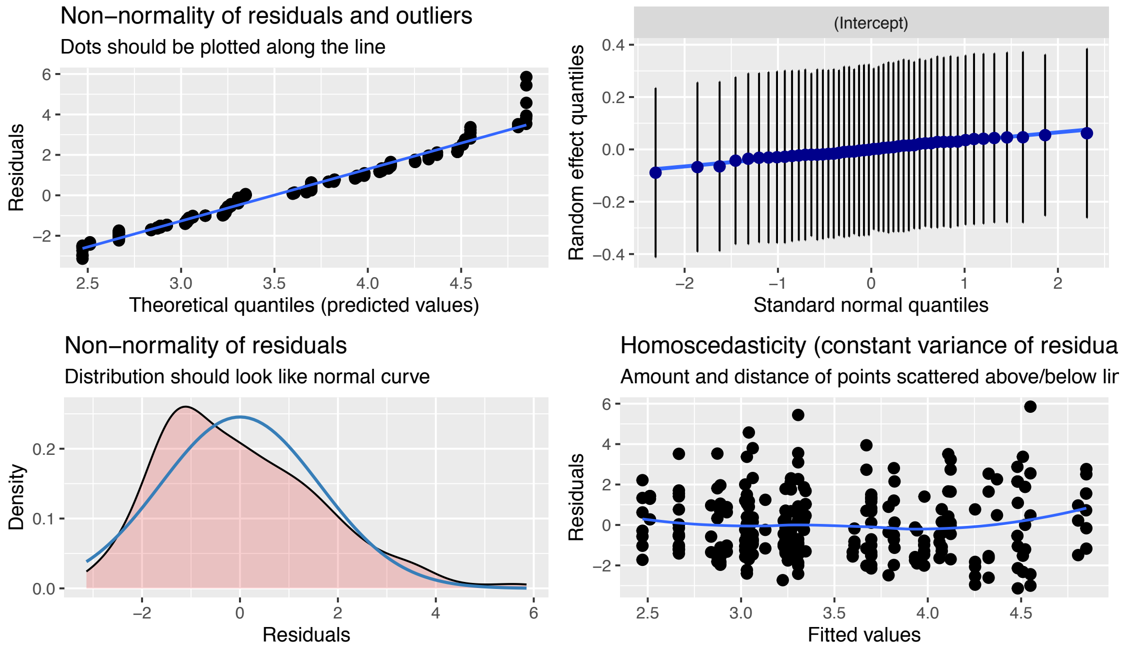


```
r.squaredGLMM(call_peaksHDDS.lm)

##              R2m       R2c
## [1,] 0.09351069 0.1442425
```

PIC analysis

```
## Vocal rate
CVw_mean=tapply(pdata$peaks, list(pdata$ID, pdata$call), mean)
CVw_sd=tapply(pdata$peaks, list(pdata$ID, pdata$call), sd)
CVw_n=tapply(pdata$peaks, list(pdata$ID, pdata$call), length)

CVw=(1+ (1/(4*CVw_n)))*(100*CVw_sd/CVw_mean)
mCVw=colMeans(CVw, na.rm = T)

CVb_mean=tapply(pdata$peaks, pdata$call, mean)
CVb_sd=tapply(pdata$peaks, pdata$call, sd)
CVb_n=tapply(pdata$peaks, pdata$call, length)

CVb=(1+ (1/(4*CVb_n)))*100*CVb_sd/CVb_mean

PICVR=CVb/mCVw

### Peak freq
CVw_mean=tapply(pdata$domFreq, list(pdata$ID, pdata$call), mean)
CVw_sd=tapply(pdata$domFreq, list(pdata$ID, pdata$call), sd)
CVw_n=tapply(pdata$domFreq, list(pdata$ID, pdata$call), length)

CVw=(1+ (1/(4*CVw_n)))*(100*CVw_sd/CVw_mean)
mCVw=colMeans(CVw, na.rm = T)
```

```
CVb_mean=tapply(pdata$domFreq, pdata$call, mean)
CVb_sd=tapply(pdata$domFreq, pdata$call, sd)
CVb_n=tapply(pdata$domFreq, pdata$call, length)

CVb=(1+ (1/(4*CVb_n)))*100*CVb_sd/CVb_mean

PICDF=CVb/mCVw

print(PICDF)

##        bark    english     french      growl       howl    italian   japane
se
##    1.921259         NA         NA   1.477974   1.605557         NA
NA
##       snarl vietnamese      whine
##    1.410404         NA   1.200940

print(PICVR)

##        bark    english     french      growl       howl    italian   japane
se
##   1.3166624  1.1500555  1.1579823  1.0687080  1.0970961  1.0278326  1.24932
82
##       snarl vietnamese      whine
##   0.9787601  1.3478024  1.9377882
```

body weight analysis

```
# log transform data
pdata$log_weight_akc = log(pdata$weight_akc)

pdata$log_peaks = log(pdata$peaks)
pdata$log_domFreq = log(pdata$domFreq)

# select subset with known weight
wdata = pdata[!is.na(pdata$weight_akc), ]


# vocal rate
weight_peaks.lm = lmer(data = pdata[pdata$Species == 'dog', ], log_peaks ~
                         log_weight_akc + (1 | ID))
m = model_parameters(weight_peaks.lm, ci_method = "kenward")
print_md(m)
```

# Fixed Effects

Parameter	Coefficient	SE	95% CI	t	df	p
(Intercept)	0.42	0.22	(-0.06, 0.90)	1.92	11.06	0.081
log weight akc	0.02	0.08	(-0.15, 0.18)	0.21	11.40	0.838

Random Effects

Parameter	Coefficient
SD (Intercept: ID)	0.09
SD (Residual)	0.45

```
anova(weight_peaks.lm, ddf = 'Kenward-Roger')

## Type III Analysis of Variance Table with Kenward-Roger's method
##                    Sum Sq   Mean Sq NumDF  DenDF F value Pr(>F)
## log_weight_akc 0.0089473 0.0089473     1 11.405  0.0439 0.8377

visu_plot = plot_model(weight_peaks.lm, type = 'diag')

plot_grid(visu_plot[[1]], visu_plot[[2]]$ID, visu_plot[[3]], visu_plot[[4]])
```

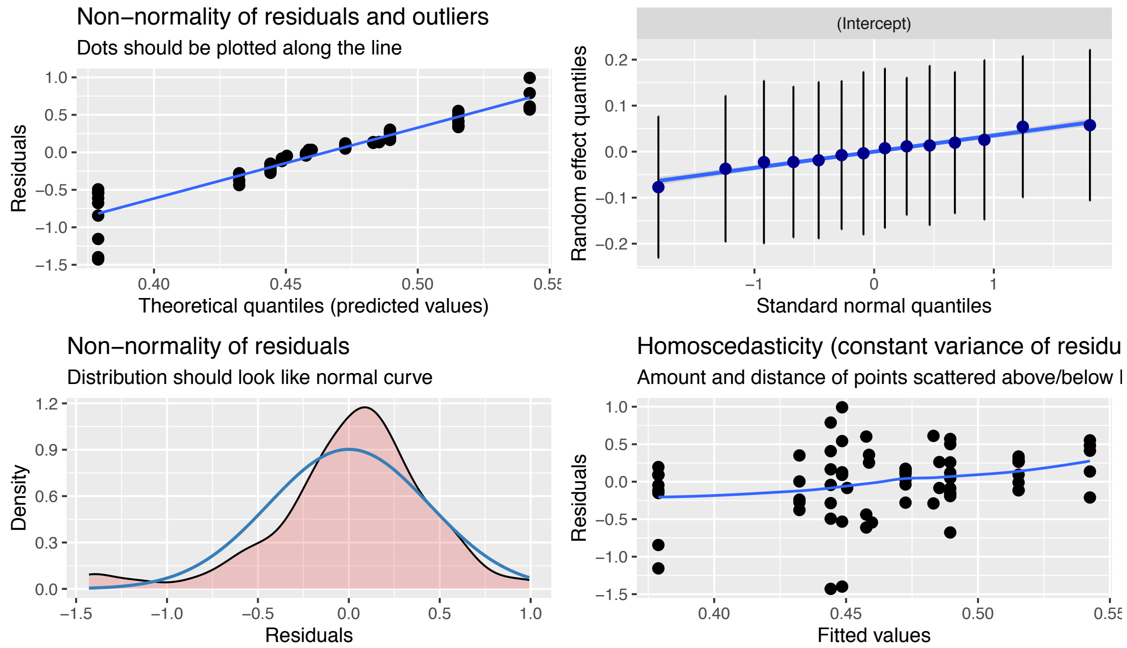


```
r.squaredGLMM(weight_peaks.lm)

##                R2m       R2c
## [1,] 0.0008019326 0.0409479

# dominant Freq
weight_domFreq.lm = lmer(data = pdata[pdata$Species == 'dog', ], log_domFreq
~
                           log_weight_akc + (1 | ID)+(1|call))
m = model_parameters(weight_domFreq.lm, ci_method = "kenward")
print_md(m)
```

Fixed Effects

Parameter	Coefficient	SE	95% CI	t	df	p

(Intercept)	0.32	0.37	(-0.48, 1.12)	0.87	12.35	0.400
log weight akc	-0.29	0.12	(-0.54, -0.03)	-2.46	12.07	0.030

# Random Effects

Parameter	Coefficient
SD (Intercept: ID)	0.33
SD (Intercept: call)	0.00
SD (Residual)	0.24

```
anova(weight_domFreq.lm, ddf = 'Kenward-Roger')

## Type III Analysis of Variance Table with Kenward-Roger's method
##                  Sum Sq Mean Sq NumDF  DenDF F value  Pr(>F)
## log_weight_akc 0.34038 0.34038     1 12.074  6.0337 0.03013 *
## ---
## Signif. codes:  0 '***' 0.001 '**' 0.01 '*' 0.05 '.' 0.1 ' ' 1

visu_plot = plot_model(weight_domFreq.lm, type = 'diag')

plot_grid(visu_plot[[1]], visu_plot[[2]]$ID, visu_plot[[3]], visu_plot[[4]])
```

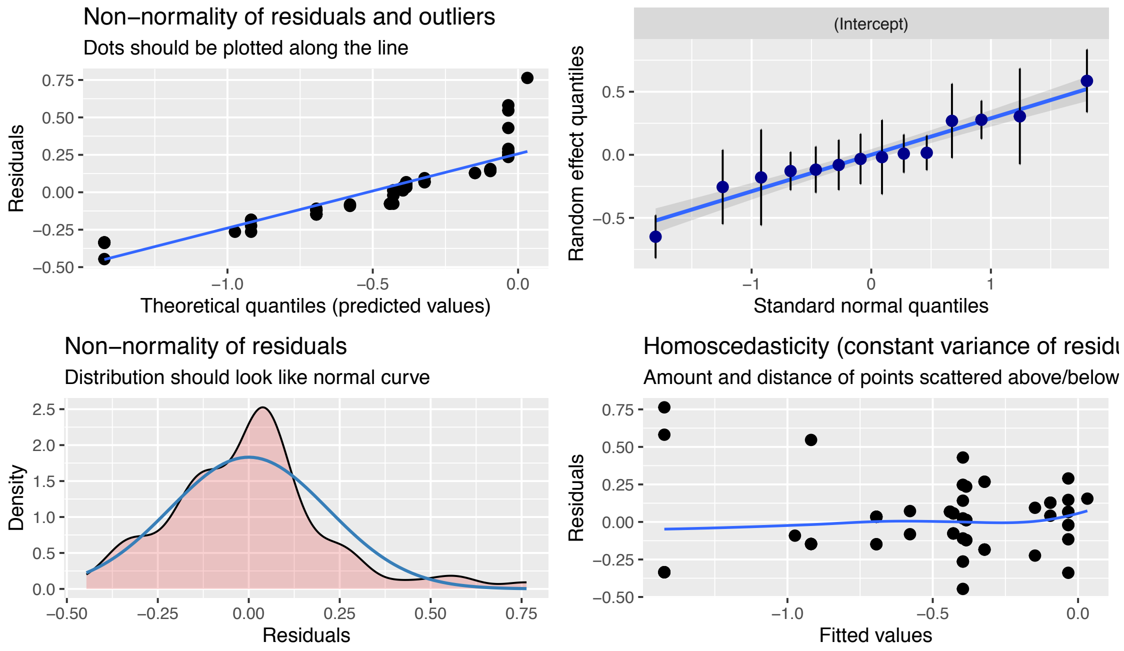


```
r.squaredGLMM(weight_domFreq.lm)

##            R2m       R2c
## [1,] 0.2523052 0.7478495
```

Cortical tracking and speech ‘intelligibility’

Accuracy in behavioural responses to speech stimuli

Human data

```
## human behaviour
human_beh.lm=lmer(bhv_max_prop~rate*type+(1|ID), xdata[xdata$species=='human'
,])
human_beh2.lm=lmer(bhv_max_prop~rate+type+(1|ID), xdata[xdata$species=='human
',])

anova(human_beh.lm, human_beh2.lm, type = 'LRT')

## Data: xdata[xdata$species == "human", ]
## Models:
## human_beh2.lm: bhv_max_prop ~ rate + type + (1 | ID)
## human_beh.lm: bhv_max_prop ~ rate * type + (1 | ID)
##               npar     AIC     BIC logLik deviance  Chisq Df Pr(>Chisq)
## human_beh2.lm    7 -29.466 -11.300 21.733  -43.466
## human_beh.lm    11 -58.527 -29.981 40.263  -80.527 37.061  4   1.75e-07 **
*
## ---
## Signif. codes:  0 '***' 0.001 '**' 0.01 '*' 0.05 '.' 0.1 ' ' 1

m=model_parameters(human_beh.lm,ci_method = "kenward")
print_md(m)
```

# Fixed Effects

Parameter	Coefficient	SE	95% CI	t	df	p
(Intercept)	0.93	0.05	(0.83, 1.03)	18.08	87.09	< .001
rate [R2]	-0.15	0.07	(-0.29, -5.86e-03)	-2.07	80.00	0.041
rate [R4]	-0.62	0.07	(-0.76, -0.48)	-8.81	80.00	< .001
type [NPC]	-0.31	0.07	(-0.45, -0.17)	-4.41	80.00	< .001
type [PNC]	-0.84	0.07	(-0.98, -0.70)	-11.92	80.00	< .001
rate [R2] × type [NPC]	-0.15	0.10	(-0.34, 0.05)	-1.47	80.00	0.146
rate [R4] × type [NPC]	0.13	0.10	(-0.07, 0.32)	1.28	80.00	0.203
rate [R2] × type [PNC]	0.13	0.10	(-0.07, 0.32)	1.28	80.00	0.203
rate [R4] × type [PNC]	0.56	0.10	(0.37, 0.76)	5.68	80.00	< .001

# Random Effects

Parameter	Coefficient
SD (Intercept: ID)	0.04
SD (Residual)	0.16

```
anova(human_beh.lm, ddf='Kenward-Roger')
```

```
## Type III Analysis of Variance Table with Kenward-Roger's method
##           Sum Sq Mean Sq NumDF DenDF F value    Pr(>F)
## rate      2.5220 1.26101     2    80  46.599 3.818e-14 ***
## type      6.0638 3.03192     2    80 112.042 < 2.2e-16 ***
## rate:type 1.1337 0.28343     4    80  10.474 7.124e-07 ***
## ---
## Signif. codes:  0 '***' 0.001 '**' 0.01 '*' 0.05 '.' 0.1 ' ' 1
```

```
visu_plot=plot_model(human_beh.lm, type='diag')

plot_grid(visu_plot[[1]],visu_plot[[2]]$ID, visu_plot[[3]], visu_plot[[4]])
```

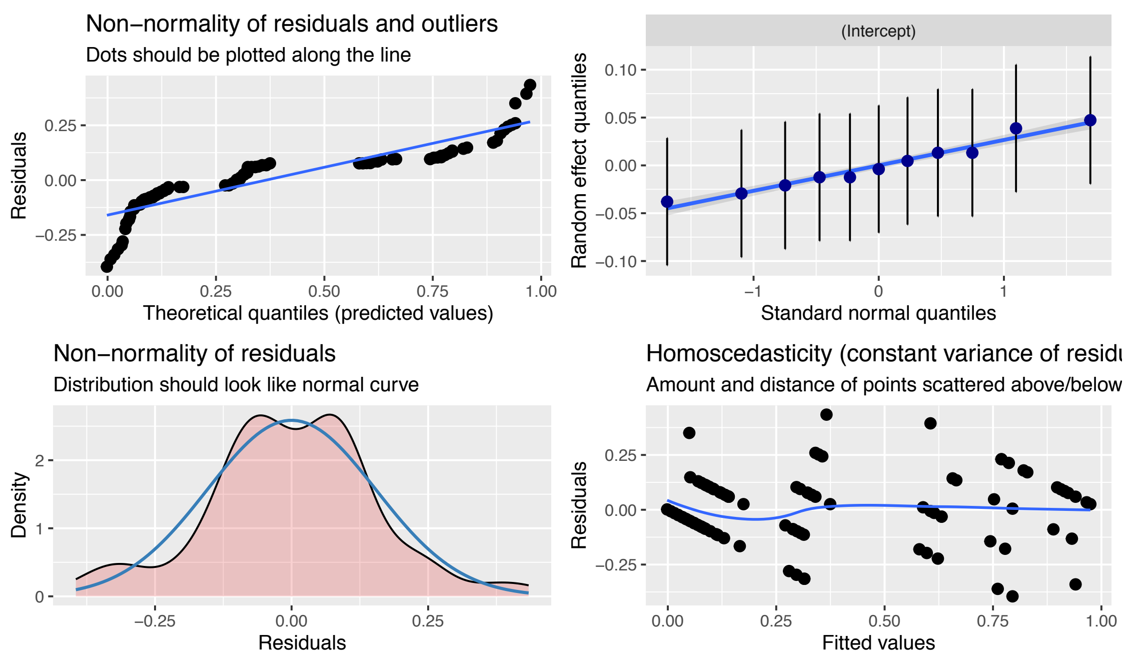


```
type.contrast <- emmeans(human_beh.lm, pairwise ~ type | rate)
print(type.contrast)
```

```
## $emmeans
## rate = R1:
##  type emmean     SE   df lower.CL upper.CL
##  CPC  0.9273 0.0513 87.1   0.8253    1.029
##  NPC  0.6182 0.0513 87.1   0.5163    0.720
##  PNC  0.0909 0.0513 87.1  -0.0110    0.193
##
## rate = R2:
##  type emmean     SE   df lower.CL upper.CL
##  CPC  0.7818 0.0513 87.1   0.6799    0.884
##  NPC  0.3273 0.0513 87.1   0.2253    0.429
##  PNC  0.0727 0.0513 87.1  -0.0292    0.175
##
## rate = R4:
##  type emmean     SE   df lower.CL upper.CL
##  CPC  0.3091 0.0513 87.1   0.2072    0.411
```

```
##   NPC  0.1273 0.0513 87.1   0.0253    0.229
##   PNC  0.0364 0.0513 87.1  -0.0656    0.138
##
## Degrees-of-freedom method: kenward-roger
## Confidence level used: 0.95
##
## $contrasts
## rate = R1:
##   contrast  estimate     SE df t.ratio p.value
##   CPC - NPC   0.3091 0.0701 80   4.407  0.0001
##   CPC - PNC   0.8364 0.0701 80  11.924  <.0001
##   NPC - PNC   0.5273 0.0701 80   7.517  <.0001
##
## rate = R2:
##   contrast  estimate     SE df t.ratio p.value
##   CPC - NPC   0.4545 0.0701 80   6.480  <.0001
##   CPC - PNC   0.7091 0.0701 80  10.109  <.0001
##   NPC - PNC   0.2545 0.0701 80   3.629  0.0014
##
## rate = R4:
##   contrast  estimate     SE df t.ratio p.value
##   CPC - NPC   0.1818 0.0701 80   2.592  0.0302
##   CPC - PNC   0.2727 0.0701 80   3.888  0.0006
##   NPC - PNC   0.0909 0.0701 80   1.296  0.4016
##
## Degrees-of-freedom method: kenward-roger
## P value adjustment: tukey method for comparing a family of 3 estimates
```

```
rate.contrast <- emmeans(human_beh.lm, pairwise ~ rate | type)
print(rate.contrast)
```

```
## $emmeans
## type = CPC:
##   rate emmean     SE   df lower.CL upper.CL
##   R1   0.9273 0.0513 87.1   0.8253    1.029
##   R2   0.7818 0.0513 87.1   0.6799    0.884
##   R4   0.3091 0.0513 87.1   0.2072    0.411
##
## type = NPC:
##   rate emmean     SE   df lower.CL upper.CL
##   R1   0.6182 0.0513 87.1   0.5163    0.720
##   R2   0.3273 0.0513 87.1   0.2253    0.429
##   R4   0.1273 0.0513 87.1   0.0253    0.229
##
## type = PNC:
##   rate emmean     SE   df lower.CL upper.CL
##   R1   0.0909 0.0513 87.1  -0.0110    0.193
##   R2   0.0727 0.0513 87.1  -0.0292    0.175
##   R4   0.0364 0.0513 87.1  -0.0656    0.138
##
```

```
## Degrees-of-freedom method: kenward-roger
## Confidence level used: 0.95
##
## $contrasts
## type = CPC:
##  contrast estimate     SE df t.ratio p.value
##  R1 - R2    0.1455 0.0701 80   2.074  0.1018
##  R1 - R4    0.6182 0.0701 80   8.813  <.0001
##  R2 - R4    0.4727 0.0701 80   6.739  <.0001
##
## type = NPC:
##  contrast estimate     SE df t.ratio p.value
##  R1 - R2    0.2909 0.0701 80   4.147  0.0002
##  R1 - R4    0.4909 0.0701 80   6.999  <.0001
##  R2 - R4    0.2000 0.0701 80   2.851  0.0151
##
## type = PNC:
##  contrast estimate     SE df t.ratio p.value
##  R1 - R2    0.0182 0.0701 80   0.259  0.9637
##  R1 - R4    0.0545 0.0701 80   0.778  0.7178
##  R2 - R4    0.0364 0.0701 80   0.518  0.8626
##
## Degrees-of-freedom method: kenward-roger
## P value adjustment: tukey method for comparing a family of 3 estimates
```

```
r.squaredGLMM(human_beh.lm)
```

```
##             R2m       R2c
## [1,] 0.7741819 0.7887686
```

Dog data

```
## dog behaviour
dog_beh.lm=lmer(bhv_max_prop~rate*type+(1|ID), xdata[xdata$species=='dog',])
dog_beh2.lm=lmer(bhv_max_prop~rate+type+(1|ID), xdata[xdata$species=='dog',])

anova(dog_beh.lm, dog_beh2.lm, type = 'LRT')
```

```
## Data: xdata[xdata$species == "dog", ]
## Models:
## dog_beh2.lm: bhv_max_prop ~ rate + type + (1 | ID)
## dog_beh.lm: bhv_max_prop ~ rate * type + (1 | ID)
##             npar     AIC     BIC logLik deviance  Chisq Df Pr(>Chisq)
## dog_beh2.lm    7 -49.633 -33.696 31.816  -63.633
## dog_beh.lm    11 -65.442 -40.398 43.721  -87.442 23.809  4  8.724e-05 ***
## ---
## Signif. codes:  0 '***' 0.001 '**' 0.01 '*' 0.05 '.' 0.1 ' ' 1
```

```
m=model_parameters(dog_beh.lm,ci_method = "kenward")
print_md(m)
```

Fixed Effects

Parameter	Coefficient	SE	95% CI	t	df	p
(Intercept)	0.79	0.07	(0.64, 0.93)	11.31	15.39	< .001
rate [R2]	-0.11	0.06	(-0.23, 0.01)	-1.84	56.00	0.072
rate [R4]	-0.34	0.06	(-0.46, -0.22)	-5.66	56.00	< .001
type [NPC]	-0.16	0.06	(-0.28, -0.04)	-2.66	56.00	0.010
type [PNC]	-0.32	0.06	(-0.44, -0.20)	-5.25	56.00	< .001
rate [R2] × type [NPC]	0.03	0.09	(-0.14, 0.20)	0.35	56.00	0.730
rate [R4] × type [NPC]	0.24	0.09	(0.07, 0.41)	2.83	56.00	0.006
rate [R2] × type [PNC]	0.20	0.09	(0.03, 0.37)	2.32	56.00	0.024
rate [R4] × type [PNC]	0.41	0.09	(0.24, 0.58)	4.81	56.00	< .001

Random Effects

Parameter	Coefficient
SD (Intercept: ID)	0.16
SD (Residual)	0.12

```
anova(dog_beh.lm, ddf='Kenward-Roger')

## Type III Analysis of Variance Table with Kenward-Roger's method
##             Sum Sq  Mean Sq NumDF DenDF F value    Pr(>F)
## rate      0.19790 0.098952     2    56  6.7687 0.0023288 **
## type      0.15895 0.079473     2    56  5.4363 0.0069555 **
## rate:type 0.36893 0.092233     4    56  6.3091 0.0002904 ***
## ---
## Signif. codes:  0 '***' 0.001 '**' 0.01 '*' 0.05 '.' 0.1 ' ' 1

visu_plot=plot_model(dog_beh.lm, type='diag')

plot_grid(visu_plot[[1]],visu_plot[[2]]$ID, visu_plot[[3]], visu_plot[[4]])
```

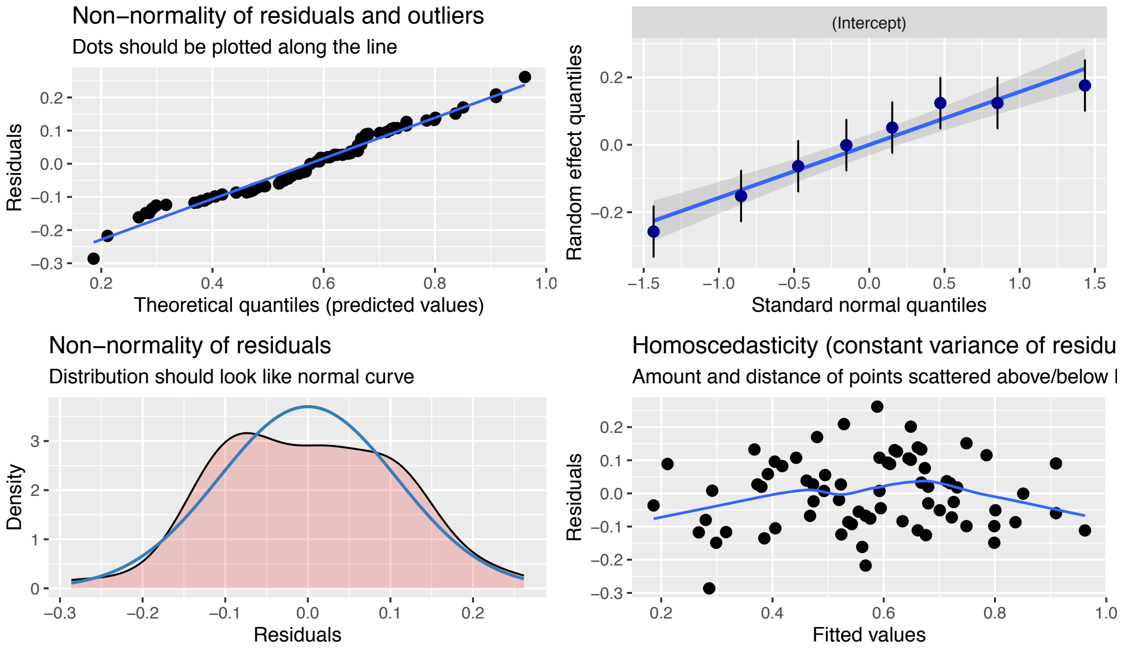


```
## post hoc
rate.contrast <- emmeans(dog_beh.lm,  pairwise~ rate | type)
print(rate.contrast)

## $emmeans
## type = CPC:
##  rate emmean     SE   df lower.CL upper.CL
##  R1    0.786 0.0695 15.4    0.638    0.934
##  R2    0.675 0.0695 15.4    0.527    0.823
##  R4    0.444 0.0695 15.4    0.296    0.592
##
## type = NPC:
##  rate emmean     SE   df lower.CL upper.CL
##  R1    0.625 0.0695 15.4    0.477    0.773
##  R2    0.544 0.0695 15.4    0.396    0.692
##  R4    0.525 0.0695 15.4    0.377    0.673
##
## type = PNC:
##  rate emmean     SE   df lower.CL upper.CL
##  R1    0.469 0.0695 15.4    0.321    0.617
##  R2    0.556 0.0695 15.4    0.408    0.704
##  R4    0.537 0.0695 15.4    0.390    0.685
##
## Degrees-of-freedom method: kenward-roger
## Confidence level used: 0.95
##
## $contrasts
## type = CPC:
##  contrast estimate     SE df t.ratio p.value
##  R1 - R2    0.1109 0.0605 56   1.835  0.1676
```

```
##   R1 - R4    0.3422 0.0605 56   5.660  <.0001
##   R2 - R4    0.2313 0.0605 56   3.825  0.0010
##
## type = NPC:
##   contrast estimate     SE df t.ratio p.value
##   R1 - R2    0.0813 0.0605 56   1.344  0.3772
##   R1 - R4    0.1000 0.0605 56   1.654  0.2319
##   R2 - R4    0.0187 0.0605 56   0.310  0.9484
##
## type = PNC:
##   contrast estimate     SE df t.ratio p.value
##   R1 - R2   -0.0875 0.0605 56  -1.447  0.3240
##   R1 - R4   -0.0688 0.0605 56  -1.137  0.4955
##   R2 - R4    0.0187 0.0605 56   0.310  0.9484
##
## Degrees-of-freedom method: kenward-roger
## P value adjustment: tukey method for comparing a family of 3 estimates
```

```
type.contrast <- emmeans(dog_beh.lm, pairwise ~ type | rate)
print(type.contrast)
```

```
## $emmeans
## rate = R1:
##   type emmean     SE   df lower.CL upper.CL
##   CPC   0.786 0.0695 15.4    0.638    0.934
##   NPC   0.625 0.0695 15.4    0.477    0.773
##   PNC   0.469 0.0695 15.4    0.321    0.617
##
## rate = R2:
##   type emmean     SE   df lower.CL upper.CL
##   CPC   0.675 0.0695 15.4    0.527    0.823
##   NPC   0.544 0.0695 15.4    0.396    0.692
##   PNC   0.556 0.0695 15.4    0.408    0.704
##
## rate = R4:
##   type emmean     SE   df lower.CL upper.CL
##   CPC   0.444 0.0695 15.4    0.296    0.592
##   NPC   0.525 0.0695 15.4    0.377    0.673
##   PNC   0.537 0.0695 15.4    0.390    0.685
##
## Degrees-of-freedom method: kenward-roger
## Confidence level used: 0.95
##
## $contrasts
## rate = R1:
##   contrast  estimate     SE df t.ratio p.value
##   CPC - NPC   0.1609 0.0605 56   2.662  0.0269
##   CPC - PNC   0.3172 0.0605 56   5.247  <.0001
##   NPC - PNC   0.1562 0.0605 56   2.585  0.0327
##
```

```
## rate = R2:
##  contrast  estimate     SE df t.ratio p.value
##  CPC - NPC   0.1313 0.0605 56   2.171  0.0851
##  CPC - PNC   0.1187 0.0605 56   1.964  0.1306
##  NPC - PNC  -0.0125 0.0605 56  -0.207  0.9767
##
## rate = R4:
##  contrast  estimate     SE df t.ratio p.value
##  CPC - NPC  -0.0813 0.0605 56  -1.344  0.3772
##  CPC - PNC  -0.0938 0.0605 56  -1.551  0.2755
##  NPC - PNC  -0.0125 0.0605 56  -0.207  0.9767
##
## Degrees-of-freedom method: kenward-roger
## P value adjustment: tukey method for comparing a family of 3 estimates
```

```
r.squaredGLMM(dog_beh.lm)
```

```
##            R2m       R2c
## [1,] 0.2091715 0.7008614
```

Dog - human differences in freq power

```
PFh=c(2.5,5.5,6.5,3,6.5,3,6,5.5,5.5,1,6.5)
PFd=c(3,2,2.5,2.5,3,5,1.5,3)

dat = data.frame(FreqPeak = c(PFd, PFh), Species = c(rep("Dog",8), rep('Human
', 11)))

# t.test(data = dat, FreqPeak~Species, var.equal = F)
# cohens_d(data = dat, FreqPeak~Species, var.equal = F)

wilcox_test(data=dat, FreqPeak~Species)
```

```
## # A tibble: 1 × 7
##   .y.      group1 group2    n1    n2 statistic      p
## * <chr>    <chr>  <chr>  <int> <int>     <dbl>  <dbl>
## 1 FreqPeak Dog    Human      8    11        18 0.0327
```

```
wilcox_effsize(data=dat, FreqPeak~Species)
```

```
## # A tibble: 1 × 7
##   .y.      group1 group2 effsize    n1    n2 magnitude
## * <chr>    <chr>  <chr>    <dbl> <int> <int> <ord>
## 1 FreqPeak Dog    Human    0.500     8    11 moderate
```

```
datmeans = data.frame(emmean = c(mean(PFd), mean(PFh)),SE= c(sd(PFd)/sqrt(8),
sd(PFh)/sqrt(11)),Species = c("Dog", 'Human'))
```

Cortical tracking under natural conditions

```
#dogs
t.test(data = cdata[cdata$species == 'dog', ],
       deltaC ~ data,
```

```
       paired = T,
       var.equal = F)

##
##  Paired t-test
##
## data:  deltaC by data
## t = -3.0239, df = 7, p-value = 0.01928
## alternative hypothesis: true mean difference is not equal to 0
## 95 percent confidence interval:
##  -0.1130825 -0.0138350
## sample estimates:
## mean difference
##     -0.06345875

cohens_d(data = cdata[cdata$species == 'dog', ],
         deltaC ~ data,
         paired = T,
         var.equal = F)

## # A tibble: 1 × 7
##   .y.    group1 group2 effsize    n1    n2 magnitude
## * <chr>  <chr>  <chr>    <dbl> <int> <int> <ord>
## 1 deltaC random real     -1.07     8     8 large

t.test(data = cdata[cdata$species == 'dog', ],
       thetaC ~ data,
       paired = T,
       var.equal = F)

##
##  Paired t-test
##
## data:  thetaC by data
## t = -0.63802, df = 7, p-value = 0.5438
## alternative hypothesis: true mean difference is not equal to 0
## 95 percent confidence interval:
##  -0.06865769  0.03948019
## sample estimates:
## mean difference
##     -0.01458875

cohens_d(data = cdata[cdata$species == 'dog', ],
         thetaC ~ data,
         paired = T,
         var.equal = F)

## # A tibble: 1 × 7
##   .y.    group1 group2 effsize    n1    n2 magnitude
## * <chr>  <chr>  <chr>    <dbl> <int> <int> <ord>
## 1 thetaC random real    -0.226     8     8 small
```

```
#humans
t.test(data = cdata[cdata$species == 'human', ],
       deltaC ~ data,
       paired = T,
       var.equal = F)

##
##  Paired t-test
##
## data:  deltaC by data
## t = -5.0439, df = 10, p-value = 0.0005037
## alternative hypothesis: true mean difference is not equal to 0
## 95 percent confidence interval:
##  -0.18534492 -0.07176599
## sample estimates:
## mean difference
##      -0.1285555

cohens_d(data = cdata[cdata$species == 'human', ],
         deltaC ~ data,
         paired = T,
         var.equal = F)

## # A tibble: 1 × 7
##   .y.    group1 group2 effsize    n1    n2 magnitude
## * <chr>  <chr>  <chr>    <dbl> <int> <int> <ord>
## 1 deltaC random real     -1.52    11    11 large

t.test(data = cdata[cdata$species == 'human', ],
       thetaC ~ data,
       paired = T,
       var.equal = F)

##
##  Paired t-test
##
## data:  thetaC by data
## t = -2.8704, df = 10, p-value = 0.01666
## alternative hypothesis: true mean difference is not equal to 0
## 95 percent confidence interval:
##  -0.069665806 -0.008776013
## sample estimates:
## mean difference
##     -0.03922091

cohens_d(data = cdata[cdata$species == 'human', ],
         thetaC ~ data,
         paired = T,
         var.equal = F)

## # A tibble: 1 × 7
##   .y.    group1 group2 effsize    n1    n2 magnitude
```

```
## * <chr>  <chr>  <chr>     <dbl> <int> <int> <ord>
## 1 thetaC random real     -0.865    11    11 large
```

Intelligibility and coherence

Human data

```
# human cacohS & bhv
human_beh_cacohS.lm=lmer(bhv_max_prop~CacohS+type+(1|ID), xdata[xdata$species
=='human',])
human_beh_cacohS2.lm=lmer(bhv_max_prop~CacohS*type+(1|ID), xdata[xdata$specie
s=='human',])

anova(human_beh_cacohS.lm, human_beh_cacohS2.lm, test = "LRT")

## Data: xdata[xdata$species == "human", ]
## Models:
## human_beh_cacohS.lm: bhv_max_prop ~ CacohS + type + (1 | ID)
## human_beh_cacohS2.lm: bhv_max_prop ~ CacohS * type + (1 | ID)
##                      npar    AIC    BIC   logLik deviance  Chisq Df Pr(>Ch
isq)
## human_beh_cacohS.lm     6 13.649 29.219 -0.82431  1.64862
## human_beh_cacohS2.lm    8 16.407 37.168 -0.20371  0.40742 1.2412  2     0.
5376

m=model_parameters(human_beh_cacohS.lm,ci_method = "kenward")
print_md(m)
```

# Fixed Effects

Parameter	Coefficient	SE	95% CI	t	df	p
(Intercept)	0.50	0.08	(0.34, 0.67)	6.04	82.25	< .001
CacohS	0.89	0.38	(0.14, 1.64)	2.35	89.46	0.021
type [NPC]	-0.31	0.06	(-0.43, -0.19)	-5.10	85.04	< .001
type [PNC]	-0.61	0.06	(-0.73, -0.49)	-9.98	85.09	< .001

# Random Effects

Parameter	Coefficient
SD (Intercept: ID)	0.00
SD (Residual)	0.25

```
anova(human_beh_cacohS.lm, ddf='Kenward-Roger')

## Type III Analysis of Variance Table with Kenward-Roger's method
##        Sum Sq Mean Sq NumDF  DenDF F value   Pr(>F)
## CacohS 0.3424 0.34243     1 89.462  5.5195  0.02101 *
## type   6.1857 3.09287     2 85.091 49.8531 4.68e-15 ***
## ---
## Signif. codes:  0 '***' 0.001 '**' 0.01 '*' 0.05 '.' 0.1 ' ' 1
```

```
visu_plot=plot_model(human_beh_cacohS.lm, type='diag')

pairs(emmeans(human_beh_cacohS.lm, specs = 'type'))

##  contrast  estimate     SE   df t.ratio p.value
##  CPC - NPC    0.313 0.0613 85.0   5.104  <.0001
##  CPC - PNC    0.613 0.0614 85.1   9.984  <.0001
##  NPC - PNC    0.300 0.0614 85.1   4.881  <.0001
##
## Degrees-of-freedom method: kenward-roger
## P value adjustment: tukey method for comparing a family of 3 estimates

plot_grid(visu_plot[[1]],visu_plot[[2]]$ID, visu_plot[[3]], visu_plot[[4]])
```

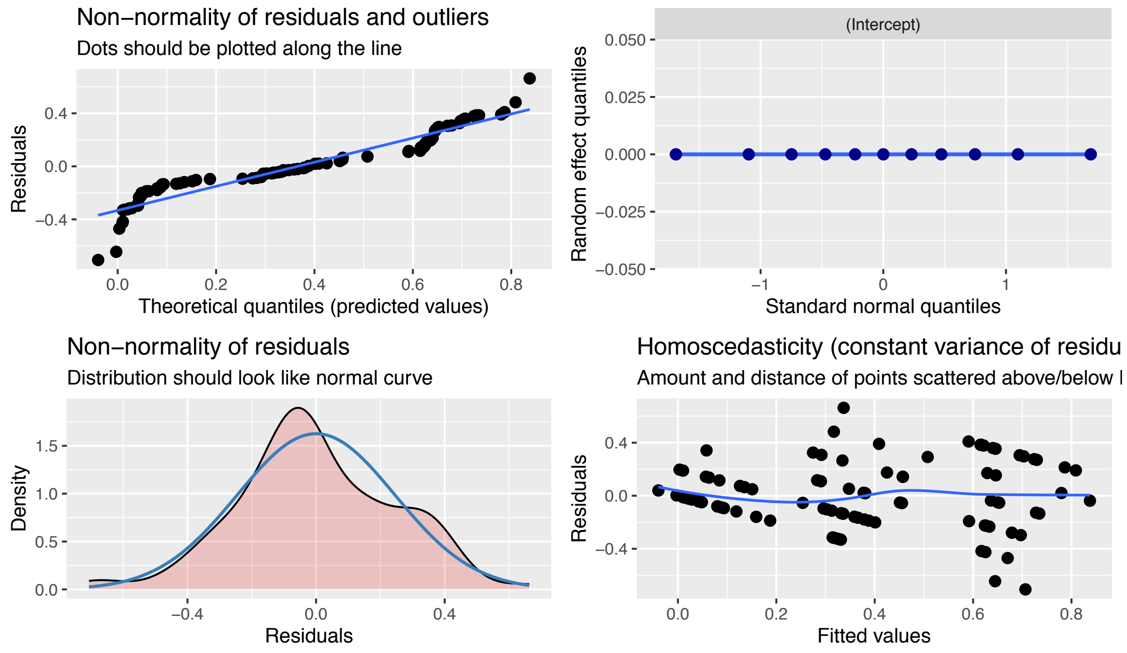


```
r.squaredGLMM(human_beh_cacohS.lm)

##            R2m       R2c
## [1,] 0.5139726 0.5139726

# human cacohW & bhv
human_beh_cacohW.lm=lmer(bhv_max_prop~CacohW+type+(1|ID), xdata[xdata$species
=='human',])
human_beh_cacohW2.lm=lmer(bhv_max_prop~CacohW*type+(1|ID), xdata[xdata$specie
s=='human',])

anova(human_beh_cacohW.lm, human_beh_cacohW2.lm, test = "LRT")

## Data: xdata[xdata$species == "human", ]
## Models:
## human_beh_cacohW.lm: bhv_max_prop ~ CacohW + type + (1 | ID)
## human_beh_cacohW2.lm: bhv_max_prop ~ CacohW * type + (1 | ID)
```

```
##                          npar    AIC    BIC  logLik deviance  Chisq Df Pr(>Chi
sq)
## human_beh_cacohW.lm      6 18.145 33.716 -3.0725   6.1450
## human_beh_cacohW2.lm     8 20.528 41.289 -2.2640   4.5279 1.6171  2     0.4
455

m=model_parameters(human_beh_cacohW.lm,ci_method = "kenward")
print_md(m)
```

# Fixed Effects

Parameter	Coefficient	SE	95% CI	t	df	p
(Intercept)	0.59	0.08	(0.43, 0.76)	7.10	58.61	< .001
CacohW	0.30	0.27	(-0.23, 0.84)	1.13	58.32	0.263
type [NPC]	-0.31	0.06	(-0.43, -0.18)	-4.90	85.55	< .001
type [PNC]	-0.60	0.06	(-0.73, -0.48)	-9.61	85.19	< .001

# Random Effects

Parameter	Coefficient
SD (Intercept: ID)	5.04e-11
SD (Residual)	0.25

```
anova(human_beh_cacohW.lm, ddf='Kenward-Roger')

## Type III Analysis of Variance Table with Kenward-Roger's method
##        Sum Sq Mean Sq NumDF  DenDF F value   Pr(>F)
## CacohW 0.0830 0.08298     1 58.324  1.2782   0.2629
## type   5.9975 2.99875     2 85.330 46.1897 2.55e-14 ***
## ---
## Signif. codes:  0 '***' 0.001 '**' 0.01 '*' 0.05 '.' 0.1 ' ' 1

visu_plot=plot_model(human_beh_cacohW.lm, type='diag')

plot_grid(visu_plot[[1]],visu_plot[[2]]$ID, visu_plot[[3]], visu_plot[[4]])
```

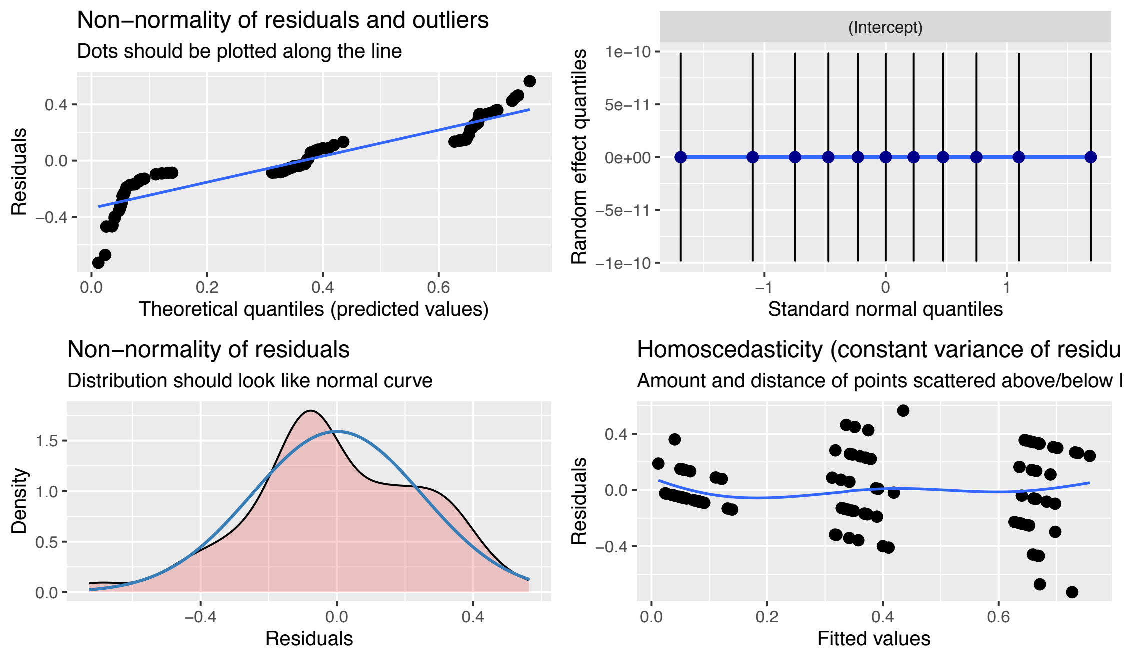


```
r.squaredGLMM(human_beh_cacohW.lm)

##            R2m       R2c
## [1,] 0.4917405 0.4917405
```

Dog data

```
# dog cacohS & bhv
dog_beh_cacohS.lm=lmer(bhv_max_prop~CacohS+type+(1|ID), xdata[xdata$species==
'dog',])
dog_beh_cacohS2.lm=lmer(bhv_max_prop~CacohS*type+(1|ID), xdata[xdata$species=
='dog',])

anova(dog_beh_cacohS.lm, dog_beh_cacohS2.lm, test = "LRT")

## Data: xdata[xdata$species == "dog", ]
## Models:
## dog_beh_cacohS.lm: bhv_max_prop ~ CacohS + type + (1 | ID)
## dog_beh_cacohS2.lm: bhv_max_prop ~ CacohS * type + (1 | ID)
##                    npar     AIC     BIC logLik deviance  Chisq Df Pr(>Chis
q)
## dog_beh_cacohS.lm     6 -41.995 -28.335 26.998  -53.995
## dog_beh_cacohS2.lm    8 -38.066 -19.852 27.033  -54.066 0.0704  2     0.96
54

m=model_parameters(dog_beh_cacohS.lm,ci_method = "kenward")
print_md(m)
```

# Fixed Effects

Parameter	Coefficient	SE	95% CI	t	df	p

(Intercept)	0.61	0.08	(0.44, 0.78)	7.44	26.43	< .001
CacohS	0.14	0.30	(-0.47, 0.75)	0.46	62.54	0.646
type [NPC]	-0.07	0.04	(-0.16, 0.01)	-1.68	61.08	0.098
type [PNC]	-0.12	0.04	(-0.20, -0.03)	-2.66	61.01	0.010

# Random Effects

Parameter	Coefficient
SD (Intercept: ID)	0.15
SD (Residual)	0.15

```
anova(dog_beh_cacohS.lm, ddf='Kenward-Roger')

## Type III Analysis of Variance Table with Kenward-Roger's method
##            Sum Sq  Mean Sq NumDF  DenDF F value  Pr(>F)
## CacohS 0.004809 0.004809     1 62.537  0.2124 0.64649
## type   0.163268 0.081634     2 61.039  3.6060 0.03309 *
## ---
## Signif. codes:  0 '***' 0.001 '**' 0.01 '*' 0.05 '.' 0.1 ' ' 1

visu_plot=plot_model(dog_beh_cacohS.lm, type='diag')

plot_grid(visu_plot[[1]],visu_plot[[2]]$ID, visu_plot[[3]], visu_plot[[4]])
```

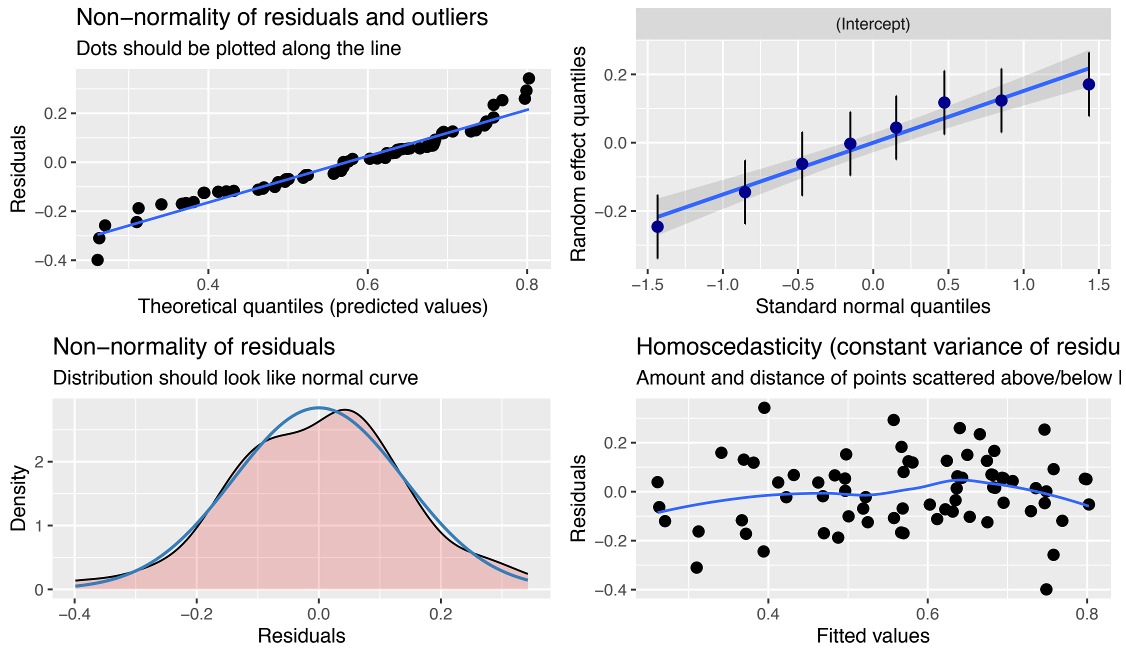


```
r.squaredGLMM(dog_beh_cacohS.lm)

##              R2m       R2c
## [1,] 0.04818749 0.5288089

# dog cacohW & bhv
dog_beh_cacohW.lm=lmer(bhv_max_prop~CacohW+type+(1|ID), xdata[xdata$species==
```

```
'dog',])
dog_beh_cacohW2.lm=lmer(bhv_max_prop~CacohW*type+(1|ID), xdata[xdata$species=
='dog',])

anova(dog_beh_cacohW.lm, dog_beh_cacohW2.lm, test = "LRT")

## Data: xdata[xdata$species == "dog", ]
## Models:
## dog_beh_cacohW.lm: bhv_max_prop ~ CacohW + type + (1 | ID)
## dog_beh_cacohW2.lm: bhv_max_prop ~ CacohW * type + (1 | ID)
##                    npar     AIC     BIC logLik deviance  Chisq Df Pr(>Chis
q)
## dog_beh_cacohW.lm     6 -46.608 -32.948 29.304  -58.608
## dog_beh_cacohW2.lm    8 -43.105 -24.892 29.553  -59.105 0.4969  2       0.
78

m=model_parameters(dog_beh_cacohW.lm,ci_method = "kenward")
print_md(m)
```

# Fixed Effects

Parameter	Coefficient	SE	95% CI	t	df	p
(Intercept)	0.52	0.08	(0.36, 0.68)	6.50	26.89	< .001
CacohW	0.55	0.25	(0.04, 1.06)	2.16	62.68	0.034
type [NPC]	-0.08	0.04	(-0.17, 2.93e-03)	-1.93	61.03	0.058
type [PNC]	-0.10	0.04	(-0.19, -0.02)	-2.35	61.04	0.022

# Random Effects

Parameter	Coefficient
SD (Intercept: ID)	0.15
SD (Residual)	0.15

```
anova(dog_beh_cacohW.lm, ddf='Kenward-Roger')

## Type III Analysis of Variance Table with Kenward-Roger's method
##          Sum Sq  Mean Sq NumDF  DenDF F value  Pr(>F)
## CacohW 0.099482 0.099482     1 62.682  4.6859 0.03423 *
## type   0.136016 0.068008     2 61.063  3.2034 0.04754 *
## ---
## Signif. codes:  0 '***' 0.001 '**' 0.01 '*' 0.05 '.' 0.1 ' ' 1

pairs(emmeans(dog_beh_cacohW.lm, specs = 'type'))

##  contrast  estimate     SE   df t.ratio p.value
##  CPC - NPC   0.0819 0.0424 61.0   1.931  0.1388
##  CPC - PNC   0.1001 0.0425 61.0   2.354  0.0560
##  NPC - PNC   0.0183 0.0437 61.1   0.419  0.9080
##
## Degrees-of-freedom method: kenward-roger
## P value adjustment: tukey method for comparing a family of 3 estimates
```

```
visu_plot=plot_model(dog_beh_cacohW.lm, type='diag')

plot_grid(visu_plot[[1]],visu_plot[[2]]$ID, visu_plot[[3]], visu_plot[[4]])
```

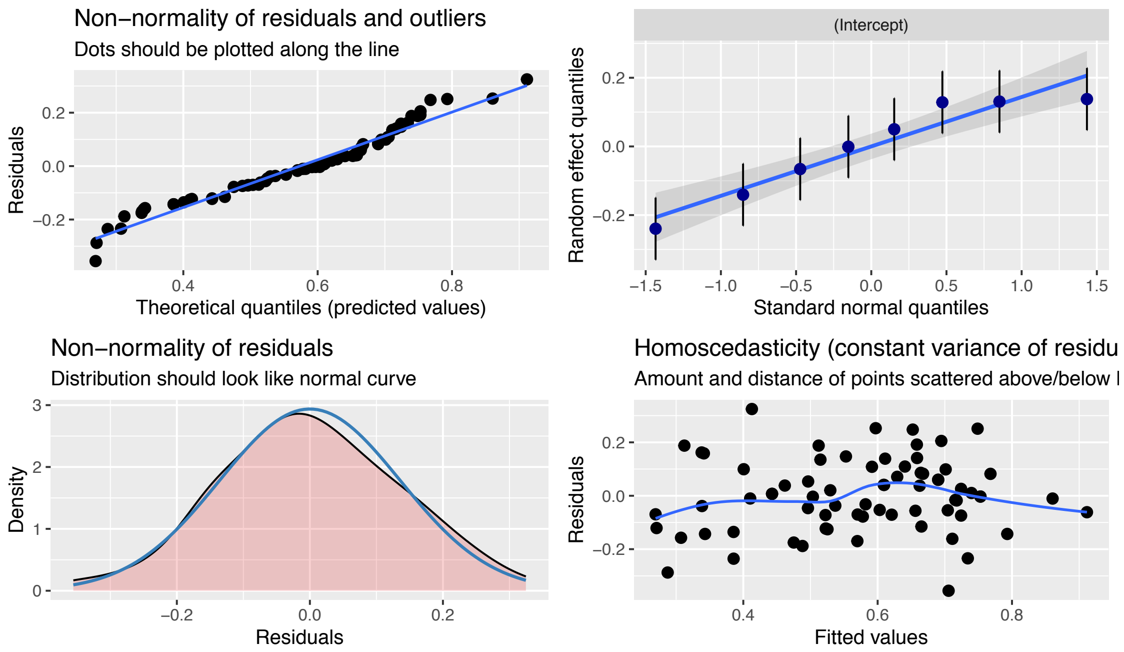


```
r.squaredGLMM(dog_beh_cacohW.lm)

##              R2m       R2c
## [1,] 0.08211683 0.5459609
```

Supplementary analyses

Electrode selection

```
edata=read.csv('electrodes_data.csv', stringsAsFactors = T, sep = ';')
edata$electrode=relevel(edata$electrode, 'FCz')

str(edata)

## 'data.frame':    99 obs. of  4 variables:
##  $ species  : Factor w/ 2 levels "Dog","Human": 1 1 1 1 1 1 1 1 1 1 ...
##  $ ID       : Factor w/ 20 levels "D1","D11","D2",..: 1 3 4 5 6 6 6 6 7 8
...
##  $ electrode: Factor w/ 11 levels "FCz","AFz","CP3",..: 6 6 6 6 1 10 9 6 6
6 ...
##  $ mtrfR    : num  0.024 0.027 0.068 0.041 0.016 0.007 0.005 0.012 0.076 0
.047 ...
```

electrode effect on mTRF

```r
human_elec.lm=lmer(mtrfR~electrode+(1|ID), data = edata[edata$species=='Human
',])
m=model_parameters(human_elec.lm,ci_method = "kenward")
print_md(m)
```

# Fixed Effects

Parameter	Coefficient	SE	95% CI	t	df	p
(Intercept)	0.08	0.01	(0.06, 0.11)	8.06	38.49	< .001
electrode [AFz]	-0.04	0.01	(-0.06, -0.01)	-3.21	67.00	0.002
electrode [CP3]	-0.04	0.01	(-0.06, -0.02)	-3.56	67.00	< .001
electrode [CP4]	-0.03	0.01	(-0.05, -7.68e-03)	-2.66	67.00	0.010
electrode [CPz]	-0.04	0.01	(-0.06, -0.01)	-3.12	67.00	0.003
electrode [FC3]	-0.01	0.01	(-0.03, 0.01)	-0.94	67.00	0.352
electrode [FC4]	-0.02	0.01	(-0.04, 7.86e-03)	-1.32	67.00	0.191
electrode [POz]	-0.02	0.01	(-0.05, 3.04e-03)	-1.76	67.54	0.083

# Random Effects

Parameter	Coefficient
SD (Intercept: ID)	0.02
SD (Residual)	0.03

```r
anova(human_elec.lm, ddf='Kenward-Roger')

## Type III Analysis of Variance Table with Kenward-Roger's method
##             Sum Sq  Mean Sq NumDF DenDF F value   Pr(>F)
## electrode 0.016541 0.002363     7 67.12  3.1732 0.005838 **
## ---
## Signif. codes:  0 '***' 0.001 '**' 0.01 '*' 0.05 '.' 0.1 ' ' 1

visu_plot=plot_model(human_elec.lm, type='diag')

plot_grid(visu_plot[[1]],visu_plot[[2]]$ID, visu_plot[[3]], visu_plot[[4]])
```

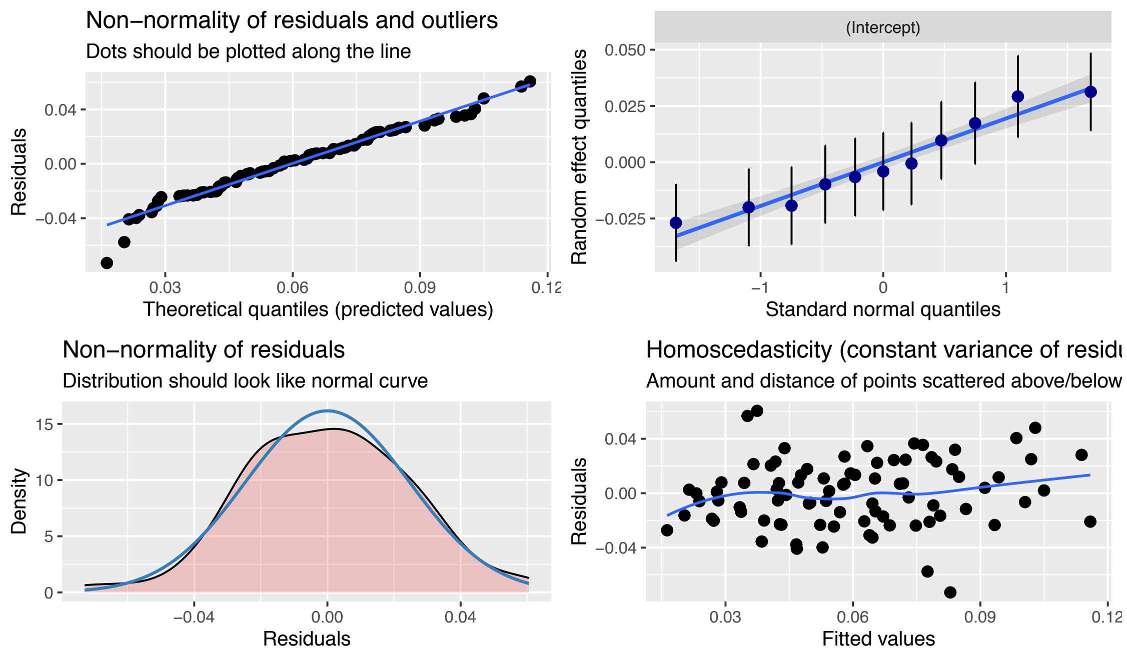


```
# post-hoc test: each electrode tested against the average of all other elect
rodes
emmeans(human_elec.lm, specs = eff ~ electrode)

## $emmeans
##  electrode emmean     SE   df lower.CL upper.CL
##  FCz       0.0846 0.0105 38.5   0.0634   0.1059
##  AFz       0.0473 0.0105 38.5   0.0260   0.0685
##  CP3       0.0432 0.0105 38.5   0.0219   0.0644
##  CP4       0.0537 0.0105 38.5   0.0325   0.0750
##  CPz       0.0484 0.0105 38.5   0.0271   0.0696
##  FC3       0.0737 0.0105 38.5   0.0525   0.0950
##  FC4       0.0693 0.0105 38.5   0.0480   0.0905
##  POz       0.0621 0.0118 50.2   0.0384   0.0858
##
## Degrees-of-freedom method: kenward-roger
## Confidence level used: 0.95
##
## $contrasts
##  contrast   estimate      SE   df t.ratio p.value
##  FCz effect  0.02435 0.00773 67.0   3.152  0.0194
##  AFz effect -0.01301 0.00773 67.0  -1.685  0.1934
##  CP3 effect -0.01711 0.00773 67.0  -2.214  0.1209
##  CP4 effect -0.00656 0.00773 67.0  -0.849  0.4558
##  CPz effect -0.01192 0.00773 67.0  -1.543  0.2039
##  FC3 effect  0.01344 0.00773 67.0   1.740  0.1934
##  FC4 effect  0.00899 0.00773 67.0   1.163  0.3319
##  POz effect  0.00183 0.00901 67.8   0.203  0.8398
##
```

```
## Degrees-of-freedom method: kenward-roger
## P value adjustment: fdr method for 8 tests
```

Effect of speech rate on cortical tracking

Human data

```
# human syll
human_cacohS.lm=lmer(CacohS~syll_rate*type+(1|ID), xdata[xdata$species=='huma
n',])
human_cacohS2.lm=lmer(CacohS~syll_rate+type+(1|ID), xdata[xdata$species=='hum
an',])

anova(human_cacohS.lm, human_cacohS2.lm, test = "LRT")

## Data: xdata[xdata$species == "human", ]
## Models:
## human_cacohS2.lm: CacohS ~ syll_rate + type + (1 | ID)
## human_cacohS.lm: CacohS ~ syll_rate * type + (1 | ID)
##                  npar     AIC     BIC logLik deviance  Chisq Df Pr(>Chisq)
## human_cacohS2.lm    6 -248.87 -233.29 130.43  -260.87
## human_cacohS.lm     8 -244.89 -224.13 130.44  -260.89 0.0229  2     0.9886

m=model_parameters(human_cacohS2.lm,ci_method = "kenward")
print_md(m)
```

# Fixed Effects

Parameter	Coefficient	SE	95% CI	t	df	p
(Intercept)	0.19	0.01	(0.16, 0.22)	15.06	37.87	< .001
syll rate	-0.02	6.69e-03	(-0.03, -7.22e-03)	-3.07	92.62	0.003
type [NPC]	-3.78e-03	0.02	(-0.04, 0.03)	-0.24	85.01	0.811
type [PNC]	7.74e-03	0.02	(-0.02, 0.04)	0.49	85.00	0.624

# Random Effects

Parameter	Coefficient
SD (Intercept: ID)	0.02
SD (Residual)	0.06

```
anova(human_cacohS2.lm, ddf='Kenward-Roger')

## Type III Analysis of Variance Table with Kenward-Roger's method
##             Sum Sq  Mean Sq NumDF  DenDF F value   Pr(>F)
## syll_rate 0.038423 0.038423     1 92.618  9.3961 0.002849 **
## type      0.002274 0.001137     2 85.008  0.2780 0.757959
## ---
## Signif. codes:  0 '***' 0.001 '**' 0.01 '*' 0.05 '.' 0.1 ' ' 1
```

```
visu_plot=plot_model(human_cacohS2.lm, type='diag')

plot_grid(visu_plot[[1]],visu_plot[[2]]$ID, visu_plot[[3]], visu_plot[[4]])
```

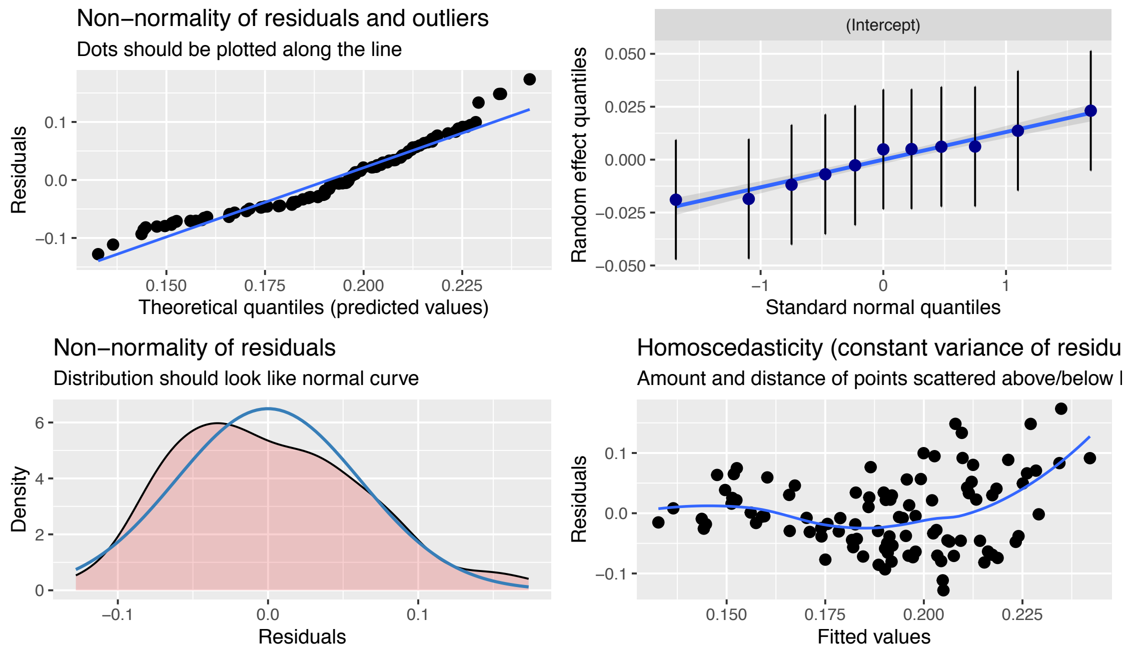


```
r.squaredGLMM(human_cacohS2.lm)

##               R2m       R2c
## [1,] 0.08941512 0.1674403

# human word
human_cacohW.lm=lmer(CacohW~word_rate*type+(1|ID), xdata[xdata$species=='huma
n',])
human_cacohW2.lm=lmer(CacohW~word_rate+type+(1|ID), xdata[xdata$species=='hum
an',])

anova(human_cacohW.lm, human_cacohW2.lm, test = "LRT")

## Data: xdata[xdata$species == "human", ]
## Models:
## human_cacohW2.lm: CacohW ~ word_rate + type + (1 | ID)
## human_cacohW.lm: CacohW ~ word_rate * type + (1 | ID)
##                  npar     AIC     BIC logLik deviance  Chisq Df Pr(>Chisq)
## human_cacohW2.lm    6 -180.29 -164.72 96.145  -192.29
## human_cacohW.lm     8 -177.68 -156.92 96.839  -193.68 1.3872  2     0.4998

m=model_parameters(human_cacohW2.lm,ci_method = "kenward")
print_md(m)
```

# Fixed Effects

Parameter	Coefficient	SE	95% CI	t	df	p

(Intercept)	0.26	0.02	(0.22, 0.31)	11.70	19.55	< .001
word rate	-0.02	8.70e-03	(-0.03, -2.35e-04)	-2.01	86.02	0.047
type [NPC]	-0.02	0.02	(-0.06, 0.02)	-1.00	85.00	0.321
type [PNC]	-9.17e-03	0.02	(-0.05, 0.03)	-0.43	85.00	0.665

# Random Effects

Parameter	Coefficient
SD (Intercept: ID)	0.06
SD (Residual)	0.09

```
anova(human_cacohW2.lm, ddf='Kenward-Roger')

## Type III Analysis of Variance Table with Kenward-Roger's method
##              Sum Sq   Mean Sq NumDF  DenDF F value  Pr(>F)
## word_rate 0.0298126 0.0298126     1 86.025  4.0602 0.04703 *
## type      0.0073494 0.0036747     2 85.000  0.5005 0.60803
## ---
## Signif. codes:  0 '***' 0.001 '**' 0.01 '*' 0.05 '.' 0.1 ' ' 1

visu_plot=plot_model(human_cacohW2.lm, type='diag')

plot_grid(visu_plot[[1]],visu_plot[[2]]$ID, visu_plot[[3]], visu_plot[[4]])
```

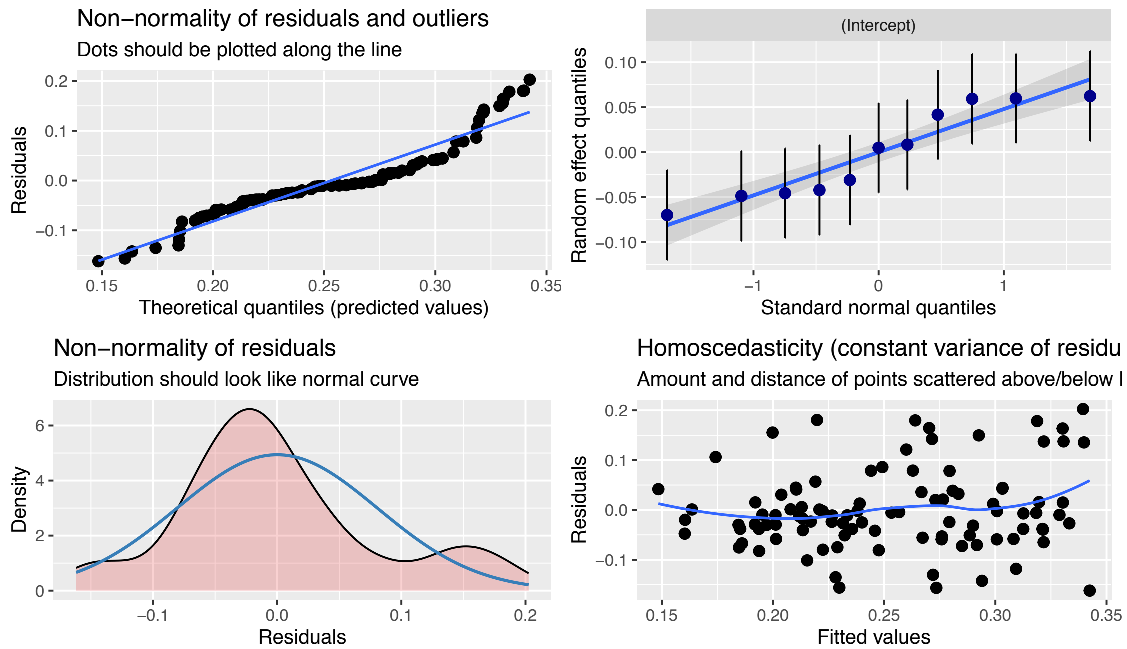


```
r.squaredGLMM(human_cacohW2.lm)

##             R2m       R2c
## [1,] 0.03583183 0.3243069
```

Dog data

```
# dog syll
dog_cacohS.lm=lmer(CacohS~syll_rate*type+(1|ID), xdata[xdata$species=='dog',]
)
dog_cacohS2.lm=lmer(CacohS~syll_rate+type+(1|ID), xdata[xdata$species=='dog',
])

anova(dog_cacohS.lm, dog_cacohS2.lm, test = "LRT")

## Data: xdata[xdata$species == "dog", ]
## Models:
## dog_cacohS2.lm: CacohS ~ syll_rate + type + (1 | ID)
## dog_cacohS.lm: CacohS ~ syll_rate * type + (1 | ID)
##                npar     AIC     BIC logLik deviance  Chisq Df Pr(>Chisq)
## dog_cacohS2.lm    6 -189.60 -175.94 100.80  -201.60
## dog_cacohS.lm     8 -186.33 -168.12 101.17  -202.33 0.7353  2     0.6923

m=model_parameters(dog_cacohS2.lm,ci_method = "kenward")
print_md(m)
```

# Fixed Effects

Parameter	Coefficient	SE	95% CI	t	df	p
(Intercept)	0.18	0.01	(0.15, 0.20)	14.16	42.68	< .001
syll rate	-0.02	7.41e-03	(-0.03, -1.74e-03)	-2.23	67.67	0.029
type [NPC]	0.03	0.02	(-4.62e-03, 0.07)	1.74	61.02	0.087
type [PNC]	0.01	0.02	(-0.02, 0.05)	0.72	61.01	0.471

# Random Effects

Parameter	Coefficient
SD (Intercept: ID)	0.00
SD (Residual)	0.06

```
anova(dog_cacohS2.lm, ddf='Kenward-Roger')

## Type III Analysis of Variance Table with Kenward-Roger's method
##              Sum Sq   Mean Sq NumDF  DenDF F value  Pr(>F)
## syll_rate 0.018760 0.0187604     1 67.667  4.9762 0.02902 *
## type      0.011508 0.0057541     2 61.015  1.5263 0.22552
## ---
## Signif. codes:  0 '***' 0.001 '**' 0.01 '*' 0.05 '.' 0.1 ' ' 1

visu_plot=plot_model(dog_cacohS.lm, type='diag')

plot_grid(visu_plot[[1]],visu_plot[[2]]$ID, visu_plot[[3]], visu_plot[[4]])
```

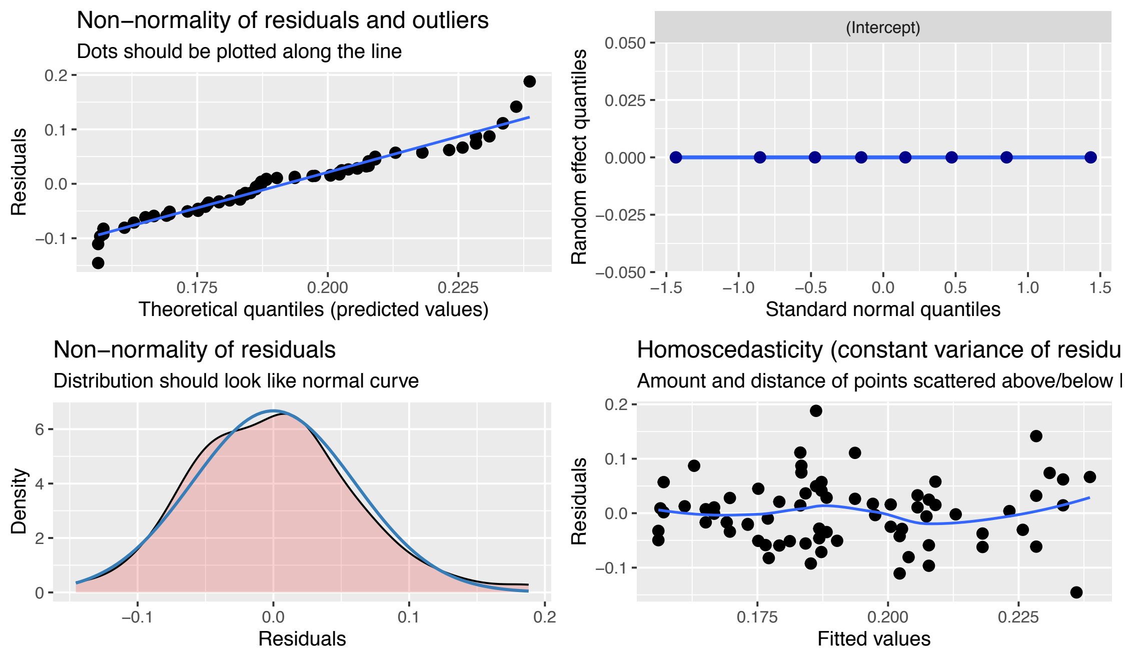


```
r.squaredGLMM(dog_cacohS.lm)

##             R2m       R2c
## [1,] 0.1137244 0.1137244

# dog word
dog_cacohW.lm=lmer(CacohW~word_rate*type+(1|ID), xdata[xdata$species=='dog',]
)
dog_cacohW2.lm=lmer(CacohW~word_rate+type+(1|ID), xdata[xdata$species=='dog',
])

anova(dog_cacohW2.lm, dog_cacohW2.lm, test = "LRT")

## Data: xdata[xdata$species == "dog", ]
## Models:
## MODEL1: CacohW ~ word_rate + type + (1 | ID)
## MODEL2: CacohW ~ word_rate + type + (1 | ID)
##        npar     AIC     BIC logLik deviance Chisq Df Pr(>Chisq)
## MODEL1    6 -170.99 -157.33 91.495  -182.99
## MODEL2    6 -170.99 -157.33 91.495  -182.99     0  0

m=model_parameters(dog_cacohW2.lm,ci_method = "kenward")
print_md(m)
```

# Fixed Effects

Parameter	Coefficient	SE	95% CI	t	df	p
(Intercept)	0.21	0.02	(0.18, 0.24)	13.69	29.97	< .001
word rate	-0.03	8.20e-03	(-0.04, -8.65e-03)	-3.05	62.96	0.003
type [NPC]	0.02	0.02	(-0.02, 0.06)	1.08	61.00	0.283

type [PNC]	-0.03	0.02	(-0.06, 0.01)	-1.30	61.00	0.197

# Random Effects

Parameter	Coefficient
SD (Intercept: ID)	0.02
SD (Residual)	0.07

```
anova(dog_cacohW2.lm, ddf='Kenward-Roger')

## Type III Analysis of Variance Table with Kenward-Roger's method
##              Sum Sq  Mean Sq NumDF  DenDF F value   Pr(>F)
## word_rate 0.043208 0.043208     1 62.957  9.3211 0.003317 **
## type      0.026476 0.013238     2 61.000  2.8558 0.065227 .
## ---
## Signif. codes:  0 '***' 0.001 '**' 0.01 '*' 0.05 '.' 0.1 ' ' 1

visu_plot=plot_model(dog_cacohW2.lm, type='diag')

plot_grid(visu_plot[[1]],visu_plot[[2]]$ID, visu_plot[[3]], visu_plot[[4]])
```

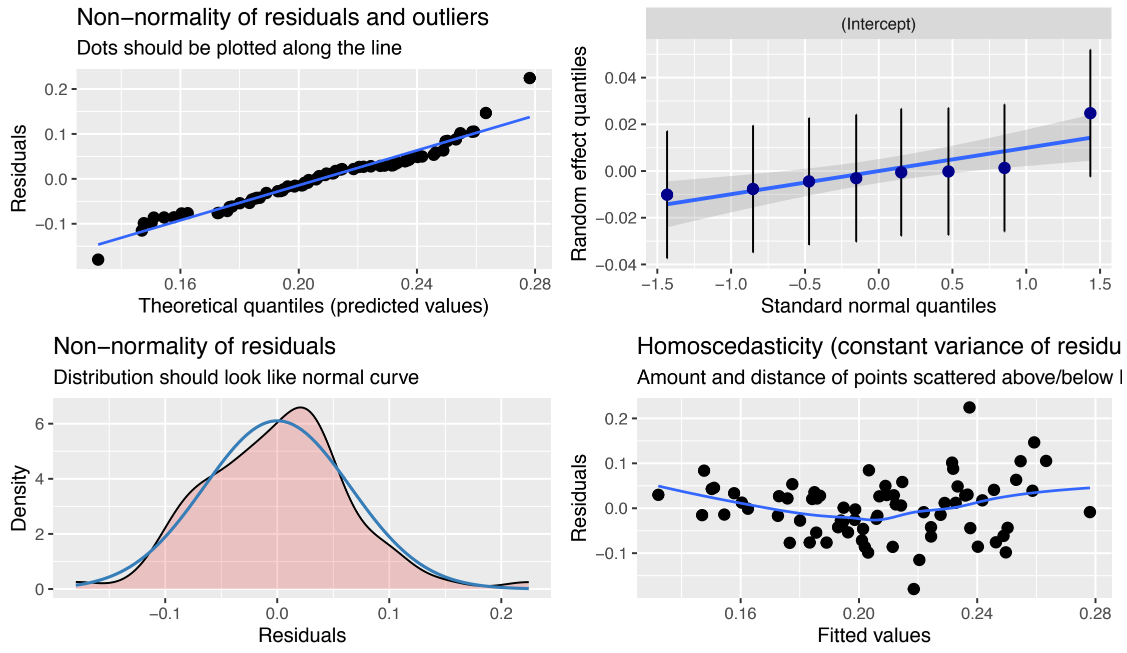


```
r.squaredGLMM(dog_cacohW.lm)

##            R2m       R2c
## [1,] 0.1982316 0.2526852
```

Production

Difference between ADS and DDS

```
#### controlling for sex (labelled 'age' in pdata)
speech_humans.lm=lmer(data=pdata[pdata$Species=='human',], peaks~voc_type+(1|
```

```
call)+(1|ID)+(1|age))
anova(speech_humans.lm,ci_method = "kenward")

## Type III Analysis of Variance Table with Satterthwaite's method
##          Sum Sq Mean Sq NumDF  DenDF F value  Pr(>F)
## voc_type 16.172  16.172     1 27.878  5.8513 0.02235 *
## ---
## Signif. codes:  0 '***' 0.001 '**' 0.01 '*' 0.05 '.' 0.1 ' ' 1

summary(speech_humans.lm)

## Linear mixed model fit by REML. t-tests use Satterthwaite's method [
## lmerModLmerTest]
## Formula: peaks ~ voc_type + (1 | call) + (1 | ID) + (1 | age)
##    Data: pdata[pdata$Species == "human", ]
##
## REML criterion at convergence: 997.7
##
## Scaled residuals:
##     Min      1Q  Median      3Q     Max
## -1.7484 -0.7354 -0.1710  0.5568  3.5785
##
## Random effects:
##  Groups   Name        Variance Std.Dev.
##  ID       (Intercept) 0.1300   0.3606
##  call     (Intercept) 0.0450   0.2121
##  age      (Intercept) 0.0862   0.2936
##  Residual             2.7638   1.6625
## Number of obs: 255, groups:  ID, 48; call, 5; age, 2
##
## Fixed effects:
##             Estimate Std. Error      df t value Pr(>|t|)
## (Intercept)   3.9199     0.2910  1.5678  13.471   0.0131 *
## voc_typeDDS  -0.6334     0.2619 27.8782  -2.419   0.0223 *
## ---
## Signif. codes:  0 '***' 0.001 '**' 0.01 '*' 0.05 '.' 0.1 ' ' 1
##
## Correlation of Fixed Effects:
##             (Intr)
## voc_typeDDS -0.453

visu_plot=plot_model(speech_humans.lm, type='diag')
plot_grid(visu_plot[[1]],visu_plot[[2]]$ID, visu_plot[[3]], visu_plot[[4]])
```

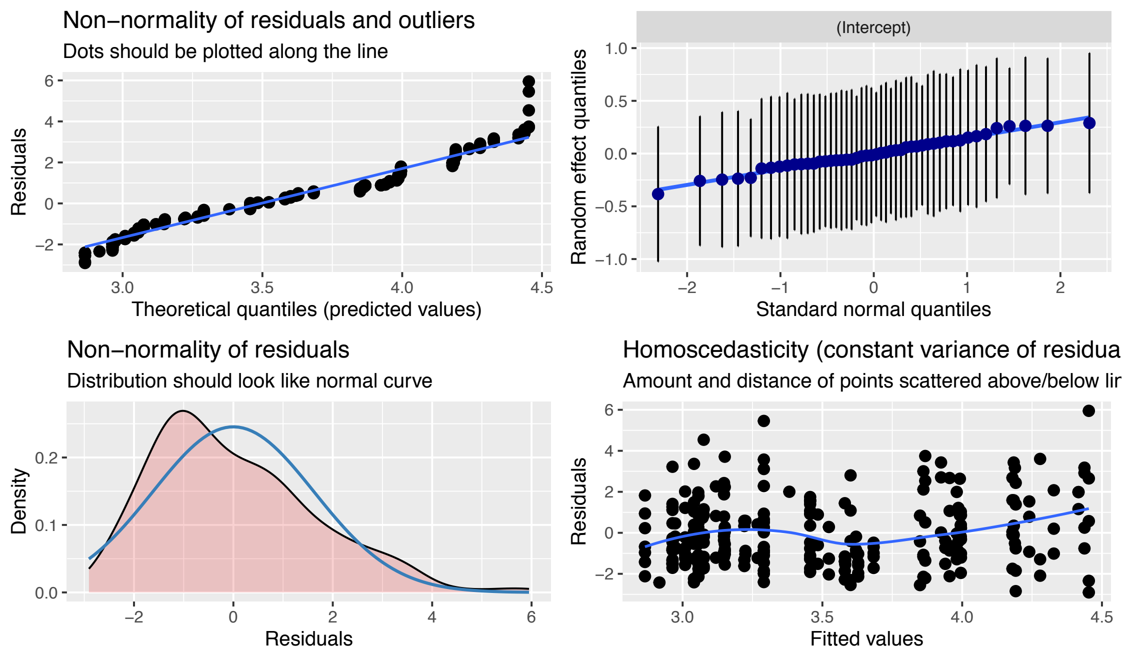


```
r.squaredGLMM(speech_humans.lm)

##              R2m       R2c
## [1,] 0.03132898 0.1149754
```
