# Supplementary material for: Dog–human vocal interactions match dogs’ sensory-motor tuning
Source: PLoS Biol. 2024 Oct 1;22(10):e3002789. doi: 10.1371/journal.pbio.3002789 (PMC11444399; doi:10.1371/journal.pbio.3002789)
Supplement: S7 Data — (PDF) [file pbio.3002789.s014.pdf]

Dog-human vocal interactions match dogs' sensory-motor tuning

Natural vocal rate in dogs and humans

1. **Differences in vocal rates between dog vocalisations, adult-directed speech (ADS) and dog-directed speech (DDS)**

ANOVA output

Parameter	Sum_Squares	df	Mean_Square	F	p
voc_type	97.80	2	48.90	23.55	< .001

Anova Table (Type 3 tests)

Model estimates

# Fixed Effects

Parameter	Coefficient	SE	95% CI	t	df	p
(Intercept)	3.97	0.20	(3.55, 4.40)	20.12	14.10	< .001
voc type [DDS]	-0.77	0.25	(-1.26, -0.27)	-3.11	61.21	0.003
voc type [DV]	-1.86	0.28	(-2.46, -1.26)	-6.77	12.16	< .001

# Random Effects

Parameter	Coefficient
SD (Intercept: ID)	0.48
SD (Intercept: call)	0.22
SD (Residual)	1.44

Post-hoc contrasts

# Estimated Marginal Means

Parameter	Marginal Means	SE	95% CI	t	df	p
ADS	3.97	0.20	(3.55, 4.40)	20.12	14.10	< .001
DDS	3.21	0.20	(2.77, 3.64)	15.94	12.56	< .001
DV	2.11	0.19	(1.68, 2.53)	10.99	10.45	< .001

# Contrasts

Parameter	Coefficient	SE	95% CI	t	df	p
ADS - DDS	0.77	0.25	(0.27, 1.26)	3.11	61.21	0.008
ADS - DV	1.86	0.28	(1.26, 2.46)	6.77	12.16	< .001
DDS - DV	1.10	0.28	(0.49, 1.71)	3.95	11.53	0.005

2. **Effect of vocal class on vocal rate in dogs**

ANOVA output

Parameter	Sum_Squares	df	Mean_Square	F	p
call	5.12	4	1.28	1.40	0.279

Anova Table (Type 3 tests)

Model estimates

Fixed Effects

Parameter	Coefficient	SE	95% CI	t	df	p
(Intercept)	2.16	0.29	(1.34, 2.98)	7.45	3.81	0.002
call [growl]	-0.70	0.52	(-1.78, 0.38)	-1.35	18.53	0.192
call [howl]	-0.45	0.59	(-1.69, 0.79)	-0.76	19.04	0.455
call [snarl]	0.85	0.60	(-0.43, 2.13)	1.42	13.97	0.177
call [whine]	0.22	0.48	(-0.81, 1.25)	0.45	14.45	0.658

Random Effects

Parameter	Coefficient
SD (Intercept: ID)	0.63
SD (Intercept: size)	0.00
SD (Residual)	0.95

3. **Effect of language on vocal rate in human ADS and DDS**

ANOVA output

Parameter	Sum_Squares	df	Mean_Square	F	p
voc_type	11.26	1	11.26	4.07	0.051
call	22.90	4	5.73	2.06	0.109
voc_type:call	26.84	4	6.71	2.41	0.070

Anova Table (Type 3 tests)

Model estimates

Fixed Effects

Parameter	Coefficient	SE	95% CI	t	df	p
(Intercept)	3.08	0.45	(1.95, 4.21)	6.86	5.43	< .001
voc type [DDS]	0.40	0.45	(-0.53, 1.33)	0.89	26.70	0.384
call [french]	1.20	0.49	(0.21, 2.19)	2.43	53.17	0.019
call [italian]	0.49	0.64	(-0.78, 1.77)	0.77	62.49	0.443
call [japanese]	0.78	0.50	(-0.22, 1.78)	1.56	58.50	0.123

call [vietnamese]	1.49	0.52	(0.46, 2.53)	2.89	59.88	0.005
voc type [DDS] × call [french]	-0.48	0.77	(-2.02, 1.05)	-0.63	58.58	0.531
voc type [DDS] × call [italian]	-1.28	0.76	(-2.82, 0.26)	-1.68	40.90	0.101
voc type [DDS] × call [japanese]	-0.99	0.63	(-2.27, 0.29)	-1.58	28.64	0.124
voc type [DDS] × call [vietnamese]	-2.08	0.70	(-3.56, -0.60)	-2.96	17.16	0.009

Random Effects

Parameter	Coefficient
SD (Intercept: ID)	0.17
SD (Intercept: age)	0.37
SD (Residual)	1.66

4. Effect of body weight on dog vocal rate

ANOVA output

Parameter	Sum_Squares	df	Mean_Square	F	p
log_weight_akc	8.95e-03	1	8.95e-03	0.04	0.838

Anova Table (Type 3 tests)

Model estimates

Fixed Effects

Parameter	Coefficient	SE	95% CI	t	df	p
(Intercept)	0.42	0.22	(-0.06, 0.90)	1.92	11.06	0.081
log weight akc	0.02	0.08	(-0.15, 0.18)	0.21	11.40	0.838

Random Effects

Parameter	Coefficient
SD (Intercept: ID)	0.09
SD (Residual)	0.45

5. Effect of body weight on dog dominant acoustic frequency

ANOVA output

Parameter	Sum_Squares	df	Mean_Square	F	p
log_weight_akc	0.34	1	0.34	6.03	0.030

Anova Table (Type 3 tests)

Model estimates

Fixed Effects

Parameter	Coefficient	SE	95% CI	t	df	p

(Intercept)	0.32	0.37	(-0.48, 1.12)	0.87	12.35	0.400
log weight akc	-0.29	0.12	(-0.54, -0.03)	-2.46	12.07	0.030

# Random Effects

Parameter	Coefficient
SD (Intercept: ID)	0.33
SD (Intercept: call)	0.00
SD (Residual)	0.24

Neural tracking and speech 'intelligibility'

6. Effect of speech type and rate on intelligibility in humans

ANOVA output

Parameter	Sum_Squares	df	Mean_Square	F	p
rate	2.52	2	1.26	46.60	< .001
type	6.06	2	3.03	112.04	< .001
rate:type	1.13	4	0.28	10.47	< .001

Anova Table (Type 3 tests)

Model estimates

# Fixed Effects

Parameter	Coefficient	SE	95% CI	t	df	p
(Intercept)	0.93	0.05	(0.83, 1.03)	18.08	87.09	< .001
rate [R2]	-0.15	0.07	(-0.29, -5.86e-03)	-2.07	80.00	0.041
rate [R4]	-0.62	0.07	(-0.76, -0.48)	-8.81	80.00	< .001
type [NPC]	-0.31	0.07	(-0.45, -0.17)	-4.41	80.00	< .001
type [PNC]	-0.84	0.07	(-0.98, -0.70)	-11.92	80.00	< .001
rate [R2] × type [NPC]	-0.15	0.10	(-0.34, 0.05)	-1.47	80.00	0.146
rate [R4] × type [NPC]	0.13	0.10	(-0.07, 0.32)	1.28	80.00	0.203
rate [R2] × type [PNC]	0.13	0.10	(-0.07, 0.32)	1.28	80.00	0.203
rate [R4] × type [PNC]	0.56	0.10	(0.37, 0.76)	5.68	80.00	< .001

# Random Effects

Parameter	Coefficient
SD (Intercept: ID)	0.04
SD (Residual)	0.16

Post-hoc contrasts

\# *Estimated Marginal Means*

Parameter	Marginal Means	SE	95% CI	t(87.09)	p
type [CPC], rate [R1]	0.93	0.05	(0.83, 1.03)	18.08	< .001
type [NPC], rate [R1]	0.62	0.05	(0.52, 0.72)	12.05	< .001
type [PNC], rate [R1]	0.09	0.05	(-0.01, 0.19)	1.77	0.080
type [CPC], rate [R2]	0.78	0.05	(0.68, 0.88)	15.25	< .001
type [NPC], rate [R2]	0.33	0.05	(0.23, 0.43)	6.38	< .001
type [PNC], rate [R2]	0.07	0.05	(-0.03, 0.17)	1.42	0.160
type [CPC], rate [R4]	0.31	0.05	(0.21, 0.41)	6.03	< .001
type [NPC], rate [R4]	0.13	0.05	(0.03, 0.23)	2.48	0.015
type [PNC], rate [R4]	0.04	0.05	(-0.07, 0.14)	0.71	0.480

\# *Contrasts*

Parameter	Coefficient	SE	95% CI	t(80.00)	p
contrast [CPC - NPC], rate [R1]	0.31	0.07	(0.17, 0.45)	4.41	< .001
contrast [CPC - PNC], rate [R1]	0.84	0.07	(0.70, 0.98)	11.92	< .001
contrast [NPC - PNC], rate [R1]	0.53	0.07	(0.39, 0.67)	7.52	< .001
contrast [CPC - NPC], rate [R2]	0.45	0.07	(0.31, 0.59)	6.48	< .001
contrast [CPC - PNC], rate [R2]	0.71	0.07	(0.57, 0.85)	10.11	< .001
contrast [NPC - PNC], rate [R2]	0.25	0.07	(0.11, 0.39)	3.63	0.001
contrast [CPC - NPC], rate [R4]	0.18	0.07	(0.04, 0.32)	2.59	0.030
contrast [CPC - PNC], rate [R4]	0.27	0.07	(0.13, 0.41)	3.89	< .001
contrast [NPC - PNC], rate [R4]	0.09	0.07	(-0.05, 0.23)	1.30	0.402

\# *Estimated Marginal Means*

Parameter	Marginal Means	SE	95% CI	t(87.09)	p
rate [R1], type [CPC]	0.93	0.05	(0.83, 1.03)	18.08	< .001
rate [R2], type [CPC]	0.78	0.05	(0.68, 0.88)	15.25	< .001
rate [R4], type [CPC]	0.31	0.05	(0.21, 0.41)	6.03	< .001
rate [R1], type [NPC]	0.62	0.05	(0.52, 0.72)	12.05	< .001
rate [R2], type [NPC]	0.33	0.05	(0.23, 0.43)	6.38	< .001
rate [R4], type [NPC]	0.13	0.05	(0.03, 0.23)	2.48	0.015
rate [R1], type [PNC]	0.09	0.05	(-0.01, 0.19)	1.77	0.080
rate [R2], type [PNC]	0.07	0.05	(-0.03, 0.17)	1.42	0.160
rate [R4], type [PNC]	0.04	0.05	(-0.07, 0.14)	0.71	0.480

\# *Contrasts*

Parameter	Coefficient	SE	95% CI	t(80.00)	p
contrast [R1 - R2], type [CPC]	0.15	0.07	(5.86e-03, 0.29)	2.07	0.102

contrast [R1 - R4], type [CPC]	0.62	0.07	(0.48, 0.76)	8.81	< .001
contrast [R2 - R4], type [CPC]	0.47	0.07	(0.33, 0.61)	6.74	< .001
contrast [R1 - R2], type [NPC]	0.29	0.07	(0.15, 0.43)	4.15	< .001
contrast [R1 - R4], type [NPC]	0.49	0.07	(0.35, 0.63)	7.00	< .001
contrast [R2 - R4], type [NPC]	0.20	0.07	(0.06, 0.34)	2.85	0.015
contrast [R1 - R2], type [PNC]	0.02	0.07	(-0.12, 0.16)	0.26	0.964
contrast [R1 - R4], type [PNC]	0.05	0.07	(-0.09, 0.19)	0.78	0.718
contrast [R2 - R4], type [PNC]	0.04	0.07	(-0.10, 0.18)	0.52	0.863

7. Effect of speech type and rate on intelligibility in dogs

ANOVA output

Parameter	Sum_Squares	df	Mean_Square	F	p
rate	0.20	2	0.10	6.77	0.002
type	0.16	2	0.08	5.44	0.007
rate:type	0.37	4	0.09	6.31	< .001

Anova Table (Type 3 tests)

Model estimates

# Fixed Effects

Parameter	Coefficient	SE	95% CI	t	df	p
(Intercept)	0.79	0.07	(0.64, 0.93)	11.31	15.39	< .001
rate [R2]	-0.11	0.06	(-0.23, 0.01)	-1.84	56.00	0.072
rate [R4]	-0.34	0.06	(-0.46, -0.22)	-5.66	56.00	< .001
type [NPC]	-0.16	0.06	(-0.28, -0.04)	-2.66	56.00	0.010
type [PNC]	-0.32	0.06	(-0.44, -0.20)	-5.25	56.00	< .001
rate [R2] × type [NPC]	0.03	0.09	(-0.14, 0.20)	0.35	56.00	0.730
rate [R4] × type [NPC]	0.24	0.09	(0.07, 0.41)	2.83	56.00	0.006
rate [R2] × type [PNC]	0.20	0.09	(0.03, 0.37)	2.32	56.00	0.024
rate [R4] × type [PNC]	0.41	0.09	(0.24, 0.58)	4.81	56.00	< .001

# Random Effects

Parameter	Coefficient
SD (Intercept: ID)	0.16
SD (Residual)	0.12

Post-hoc contrasts

\# *Estimated Marginal Means*

Parameter	Marginal Means	SE	95% CI	t(15.39)	p
type [CPC], rate [R1]	0.79	0.07	(0.64, 0.93)	11.31	< .001
type [NPC], rate [R1]	0.63	0.07	(0.48, 0.77)	8.99	< .001
type [PNC], rate [R1]	0.47	0.07	(0.32, 0.62)	6.74	< .001
type [CPC], rate [R2]	0.68	0.07	(0.53, 0.82)	9.71	< .001
type [NPC], rate [R2]	0.54	0.07	(0.40, 0.69)	7.82	< .001
type [PNC], rate [R2]	0.56	0.07	(0.41, 0.70)	8.00	< .001
type [CPC], rate [R4]	0.44	0.07	(0.30, 0.59)	6.38	< .001
type [NPC], rate [R4]	0.53	0.07	(0.38, 0.67)	7.55	< .001
type [PNC], rate [R4]	0.54	0.07	(0.39, 0.69)	7.73	< .001

\# *Contrasts*

Parameter	Coefficient	SE	95% CI	t(56.00)	p
contrast [CPC - NPC], rate [R1]	0.16	0.06	(0.04, 0.28)	2.66	0.027
contrast [CPC - PNC], rate [R1]	0.32	0.06	(0.20, 0.44)	5.25	< .001
contrast [NPC - PNC], rate [R1]	0.16	0.06	(0.04, 0.28)	2.58	0.033
contrast [CPC - NPC], rate [R2]	0.13	0.06	(0.01, 0.25)	2.17	0.085
contrast [CPC - PNC], rate [R2]	0.12	0.06	(-2.35e-03, 0.24)	1.96	0.131
contrast [NPC - PNC], rate [R2]	-0.01	0.06	(-0.13, 0.11)	-0.21	0.977
contrast [CPC - NPC], rate [R4]	-0.08	0.06	(-0.20, 0.04)	-1.34	0.377
contrast [CPC - PNC], rate [R4]	-0.09	0.06	(-0.21, 0.03)	-1.55	0.275
contrast [NPC - PNC], rate [R4]	-0.01	0.06	(-0.13, 0.11)	-0.21	0.977

\# *Estimated Marginal Means*

Parameter	Marginal Means	SE	95% CI	t(15.39)	p
rate [R1], type [CPC]	0.79	0.07	(0.64, 0.93)	11.31	< .001
rate [R2], type [CPC]	0.68	0.07	(0.53, 0.82)	9.71	< .001
rate [R4], type [CPC]	0.44	0.07	(0.30, 0.59)	6.38	< .001
rate [R1], type [NPC]	0.63	0.07	(0.48, 0.77)	8.99	< .001
rate [R2], type [NPC]	0.54	0.07	(0.40, 0.69)	7.82	< .001
rate [R4], type [NPC]	0.53	0.07	(0.38, 0.67)	7.55	< .001
rate [R1], type [PNC]	0.47	0.07	(0.32, 0.62)	6.74	< .001
rate [R2], type [PNC]	0.56	0.07	(0.41, 0.70)	8.00	< .001
rate [R4], type [PNC]	0.54	0.07	(0.39, 0.69)	7.73	< .001

\# *Contrasts*

Parameter	Coefficient	SE	95% CI	t(56.00)	p
contrast [R1 - R2], type [CPC]	0.11	0.06	(-0.01, 0.23)	1.84	0.168

contrast [R1 - R4], type [CPC]	0.34	0.06	(0.22, 0.46)	5.66	< .001
contrast [R2 - R4], type [CPC]	0.23	0.06	(0.11, 0.35)	3.83	< .001
contrast [R1 - R2], type [NPC]	0.08	0.06	(-0.04, 0.20)	1.34	0.377
contrast [R1 - R4], type [NPC]	0.10	0.06	(-0.02, 0.22)	1.65	0.232
contrast [R2 - R4], type [NPC]	0.02	0.06	(-0.10, 0.14)	0.31	0.948
contrast [R1 - R2], type [PNC]	-0.09	0.06	(-0.21, 0.03)	-1.45	0.324
contrast [R1 - R4], type [PNC]	-0.07	0.06	(-0.19, 0.05)	-1.14	0.495
contrast [R2 - R4], type [PNC]	0.02	0.06	(-0.10, 0.14)	0.31	0.948

8. Effect of speech rate on cortical tracking in humans

Syllable level - ANOVA output

Parameter	Sum_Squares	df	Mean_Square	F	p
syll_rate	0.04	1	0.04	9.62	0.003
type	2.27e-03	2	1.14e-03	0.28	0.758

Anova Table (Type 3 tests)

Syllable level - Model estimates

# Fixed Effects

Parameter	Coefficient	SE	95% CI	t	df	p
(Intercept)	0.19	0.01	(0.16, 0.22)	15.06	37.87	< .001
syll rate	-0.02	6.69e-03	(-0.03, -7.22e-03)	-3.07	92.62	0.003
type [NPC]	-3.78e-03	0.02	(-0.04, 0.03)	-0.24	85.01	0.811
type [PNC]	7.74e-03	0.02	(-0.02, 0.04)	0.49	85.00	0.624

# Random Effects

Parameter	Coefficient
SD (Intercept: ID)	0.02
SD (Residual)	0.06

Word level - ANOVA output

Parameter	Sum_Squares	df	Mean_Square	F	p
word_rate	0.03	1	0.03	4.07	0.047
type	7.35e-03	2	3.67e-03	0.50	0.608

Anova Table (Type 3 tests)

Word level - Model estimates

# Fixed Effects

Parameter	Coefficient	SE	95% CI	t	df	p

(Intercept)	0.26	0.02	(0.22, 0.31)	11.70	19.55	< .001
word rate	-0.02	8.70e-03	(-0.03, -2.35e-04)	-2.01	86.02	0.047
type [NPC]	-0.02	0.02	(-0.06, 0.02)	-1.00	85.00	0.321
type [PNC]	-9.17e-03	0.02	(-0.05, 0.03)	-0.43	85.00	0.665

Random Effects

Parameter	Coefficient
SD (Intercept: ID)	0.06
SD (Residual)	0.09

9. Effect of speech rate on cortical tracking in dogs

Syllable level - ANOVA output

Parameter	Sum_Squares	df	Mean_Square	F	p
syll_rate	0.02	1	0.02	5.22	0.026
type	0.01	2	5.75e-03	1.53	0.225

Anova Table (Type 3 tests)

Syllable level - Model estimates

Fixed Effects

Parameter	Coefficient	SE	95% CI	t	df	p
(Intercept)	0.18	0.01	(0.15, 0.20)	14.16	42.68	< .001
syll rate	-0.02	7.41e-03	(-0.03, -1.74e-03)	-2.23	67.67	0.029
type [NPC]	0.03	0.02	(-4.62e-03, 0.07)	1.74	61.02	0.087
type [PNC]	0.01	0.02	(-0.02, 0.05)	0.72	61.01	0.471

Random Effects

Parameter	Coefficient
SD (Intercept: ID)	0.00
SD (Residual)	0.06

Word level - ANOVA output

Parameter	Sum_Squares	df	Mean_Square	F	p
word_rate	0.04	1	0.04	9.42	0.003
type	0.03	2	0.01	2.86	0.065

Anova Table (Type 3 tests)

Word level - Model estimates

Fixed Effects

Parameter	Coefficient	SE	95% CI	t	df	p

(Intercept)	0.21	0.02	(0.18, 0.24)	13.69	29.97	< .001
word rate	-0.03	8.20e-03	(-0.04, -8.65e-03)	-3.05	62.96	0.003
type [NPC]	0.02	0.02	(-0.02, 0.06)	1.08	61.00	0.283
type [PNC]	-0.03	0.02	(-0.06, 0.01)	-1.30	61.00	0.197

# Random Effects

Parameter	Coefficient
SD (Intercept: ID)	0.02
SD (Residual)	0.07

10. Effect of cortical tracking on intelligibility in humans

Syllable level - ANOVA output

Parameter	Sum_Squares	df	Mean_Square	F	p
CacohS	0.37	1	0.37	5.89	0.017
type	6.19	2	3.09	49.86	< .001

Anova Table (Type 3 tests)

Syllable level - Model estimates

# Fixed Effects

Parameter	Coefficient	SE	95% CI	t	df	p
(Intercept)	0.50	0.08	(0.34, 0.67)	6.04	82.25	< .001
CacohS	0.89	0.38	(0.14, 1.64)	2.35	89.46	0.021
type [NPC]	-0.31	0.06	(-0.43, -0.19)	-5.10	85.04	< .001
type [PNC]	-0.61	0.06	(-0.73, -0.49)	-9.98	85.09	< .001

# Random Effects

Parameter	Coefficient
SD (Intercept: ID)	0.00
SD (Residual)	0.25

Post-hoc contrasts

# Estimated Marginal Means

Parameter	Marginal Means	SE	95% CI	t	df	p
CPC	0.67	0.04	(0.59, 0.76)	15.55	60.13	< .001
NPC	0.36	0.04	(0.27, 0.45)	8.33	60.16	< .001
PNC	0.06	0.04	(-0.03, 0.15)	1.41	60.20	0.163

# Contrasts

Parameter	Coefficient	SE	95% CI	t	df	p
CPC - NPC	0.31	0.06	(0.19, 0.43)	5.10	85.04	< .001

CPC - PNC	0.61	0.06	(0.49, 0.73)	9.98	85.09	< .001
NPC - PNC	0.30	0.06	(0.18, 0.42)	4.88	85.14	< .001

Word level - ANOVA output

Parameter	Sum_Squares	df	Mean_Square	F	p
CacohW	0.09	1	0.09	1.41	0.237
type	6.00	2	3.00	46.20	< .001

Anova Table (Type 3 tests)

Word level - Model estimates

# Fixed Effects

Parameter	Coefficient	SE	95% CI	t	df	p
(Intercept)	0.59	0.08	(0.43, 0.76)	7.10	58.61	< .001
CacohW	0.30	0.27	(-0.23, 0.84)	1.13	58.32	0.263
type [NPC]	-0.31	0.06	(-0.43, -0.18)	-4.90	85.55	< .001
type [PNC]	-0.60	0.06	(-0.73, -0.48)	-9.61	85.19	< .001

# Random Effects

Parameter	Coefficient
SD (Intercept: ID)	5.04e-11
SD (Residual)	0.25

11. Effect of cortical tracking on intelligibility in dogs

Syllable level - ANOVA output

Parameter	Sum_Squares	df	Mean_Square	F	p
CacohS	4.85e-03	1	4.85e-03	0.21	0.645
type	0.16	2	0.08	3.61	0.033

Anova Table (Type 3 tests)

Syllable level - Model estimates

# Fixed Effects

Parameter	Coefficient	SE	95% CI	t	df	p
(Intercept)	0.61	0.08	(0.44, 0.78)	7.44	26.43	< .001
CacohS	0.14	0.30	(-0.47, 0.75)	0.46	62.54	0.646
type [NPC]	-0.07	0.04	(-0.16, 0.01)	-1.68	61.08	0.098
type [PNC]	-0.12	0.04	(-0.20, -0.03)	-2.66	61.01	0.010

# Random Effects

Parameter	Coefficient

SD (Intercept: ID)	0.15
SD (Residual)	0.15

Word level - ANOVA output

Parameter	Sum_Squares	df	Mean_Square	F	p
CacohW	0.10	1	0.10	4.73	0.033
type	0.14	2	0.07	3.20	0.048

Anova Table (Type 3 tests)

Word level - Model estimates

Fixed Effects

Parameter	Coefficient	SE	95% CI	t	df	p
(Intercept)	0.52	0.08	(0.36, 0.68)	6.50	26.89	< .001
CacohW	0.55	0.25	(0.04, 1.06)	2.16	62.68	0.034
type [NPC]	-0.08	0.04	(-0.17, 2.93e-03)	-1.93	61.03	0.058
type [PNC]	-0.10	0.04	(-0.19, -0.02)	-2.35	61.04	0.022

Random Effects

Parameter	Coefficient
SD (Intercept: ID)	0.15
SD (Residual)	0.15

Post-hoc contrasts

Estimated Marginal Means

Parameter	Marginal Means	SE	95% CI	t	df	p
CPC	0.63	0.06	(0.50, 0.77)	10.57	9.93	< .001
NPC	0.55	0.06	(0.42, 0.69)	9.17	10.10	< .001
PNC	0.53	0.06	(0.40, 0.67)	8.86	10.13	< .001

Contrasts

Parameter	Coefficient	SE	95% CI	t	df	p
CPC - NPC	0.08	0.04	(-2.93e-03, 0.17)	1.93	61.03	0.139
CPC - PNC	0.10	0.04	(0.02, 0.19)	2.35	61.04	0.056
NPC - PNC	0.02	0.04	(-0.07, 0.11)	0.42	61.13	0.908
